# Mimetics of ADP-Ribosylated Histidine through Copper(I)-Catalyzed Click Chemistry

**DOI:** 10.1021/acs.orglett.2c01300

**Published:** 2022-05-19

**Authors:** Hugo Minnee, Johannes G. M. Rack, Gijsbert A. van der Marel, Herman S. Overkleeft, Jeroen D. C. Codée, Ivan Ahel, Dmitri V. Filippov

**Affiliations:** † Bio-Organic Synthesis, Leiden Institute of Chemistry, Leiden University, P.O. Box 9502, 2300 RA Leiden, The Netherlands; ‡ Sir William Dunn School of Pathology, University of Oxford, South Parks Road, Oxford OX1 3RE, United Kingdom

## Abstract

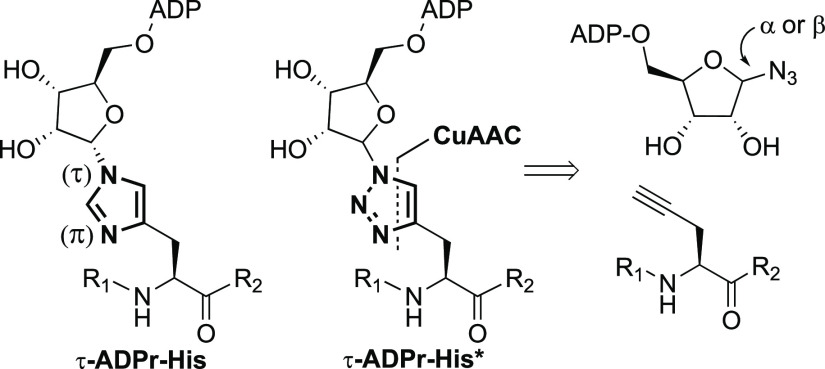

A convergent synthesis provided nearly perfect τ-ADP-ribosylated histidine isosteres (His*-τ-ADPr) via a copper(I)-catalyzed cycloaddition between an azido-ADP-ribosyl analogue and an oligopeptide carrying a propargyl glycine. Both α- and β-configured azido-ADP-ribosyl analogues have been synthesized. The former required participation of the C-2 ester functionality during glycosylation, while the latter was obtained in high stereoselectivity from an imidate donor with a nonparticipating *para*-methoxy benzyl ether. Four His*-τ-ADPr peptides were screened against a library of human ADP-ribosyl hydrolases.

A wide array of posttranslational modifications (PTMs), varying from small alterations (e.g., methylation) to the introduction of complete proteins (e.g., ubiquitination), are essential for regulatory control of protein activity. Although nicotinamide adenine dinucleotide (NAD^+^) is commonly known for its role as a redox cofactor, it also participates in a PTM called adenosine diphosphate-ribosylation (ADP-ribosylation). In this PTM, the adenosine diphosphate ribose (ADPr) moiety is covalently attached to specific amino acid side chains of the targeted protein by substitution of the nicotinamide residue. In humans, this process is primarily facilitated by ADP-ribosyl transferases termed PARPs,^1^ and the resulting modification can be either mono-ADP-ribosylation (MARylation) when a single ADPr molecule is introduced or poly-ADP-ribosylation (PARylation) when longer linear or branched ADPr polymers are formed. The latter process can be mediated by only a small subset of PARPs: PARP1, PARP2, PARP5a, and PARP5b. Ever since the first isolation of poly-ADPr chains^2^ and the identification of the transferase enzyme,^3^ this PTM has received ever increasing attention because of the many roles it plays in various biological events. However, progress has been slowed down due to the lack of appropriate tools to study this modification. Advances in mass spectrometry revealed the breadth of possible amino acid acceptors, including glutamate, aspartate, arginine,^4^ lysine,^5^ cysteine,^6^ histidine,^7,8^ tyrosine,^9,10^ and serine.^11^ A complex regulatory network of proteins “writing”, “reading”, and “erasing” the ADP-ribosylation^12,13^ is responsible for the huge structural diversity of the “ADP-ribosylome”. Due to such structural diversity, ADP-ribosylation allows for the spatiotemporal and context specific regulation of a wide variety of cellular processes including DNA damage response, replication, transcription, and cellular signaling (e.g., WNT signaling).^14−17^ Among the different acceptor residues, serine has emerged as the primary target in DNA damage-induced ADP-ribosylation.^18−20^ Recent proteomic studies have also drawn attention toward the occurrence of lower-frequency modifications at tyrosine and histidine sites.^7,8,21^ The identification of these new flavors of stress-induced ADP-ribosylation is suggestive of a specialized control mechanism for subprocesses within the DNA-damage response (DDR). For an identification and characterization of the responsible “writers”, “readers”, and “erasers” as well as an examination of their cellular function, well-defined molecular tools are indispensable.

To generate tools to study histidine ADP-ribosylation, we reasoned that click chemistry could be exploited to create a nearly perfect isostere of ADP-ribosylated histidine (Figure 1A). Although the exact structure of ADP-ribosylated histidine (His-ADPr) has not yet been determined, we hypothesize here that ADPr is introduced at the τ-position of the imidazole functionality, most likely via an α-configured linkage. This hypothesis is based on the isolation of ribosylated and ADP-ribosylated histidine metabolites^22,23^ combined with the known stereospecificity of PARP enzymes. The suspected 1,4-substitution pattern can be mimicked via a Cu(I)-catalyzed azide–alkyne cycloaddition (CuAAC), known for its high regioselectivity, with an azido-ADPr analogue and an oligopeptide carrying a propargyl click handle at a specific position. The use of CuAAC has been successfully implemented before in the synthesis of ADP-ribosylated oligopeptides and proteins.^24−26^ Here, the convergent syntheses of both α- and β-configured ADP-ribosylated histidine mimetics **1–4** (Figure 1B) are described.

**Figure 1:**
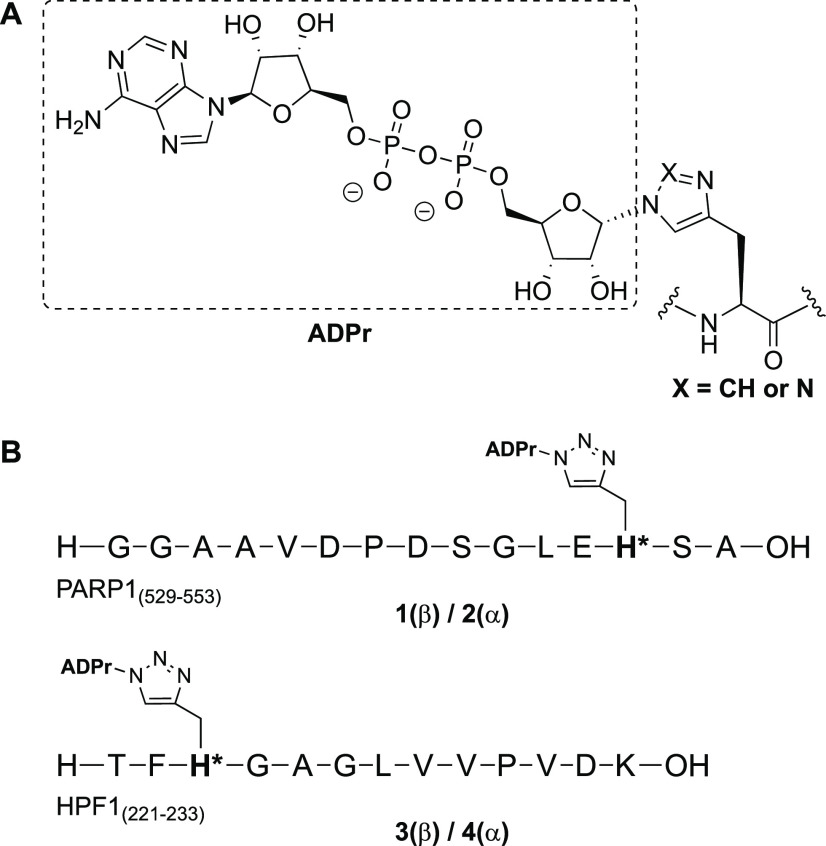
(A) Proposed structure of ADP-ribosylated histidine (X = CH) under physiological conditions. Based on histidine metabolites and the α-selective introduction of PARPs, it is suggested that ADPr is attached to the imidazole functionality at the τ-nitrogen through an α-configured glycosidic bond. An ADP-ribosylated histidine isostere (X = N) can be obtained through CuAAC, where the imidazole functionality is replaced by a triazole moiety. (B) Structures of the ADP-ribosylated histidine mimetics described in this paper.

The target oligopeptides **1**–**4** are based on two potential His-ADPr sites, located on histone PARylation factor 1 (HPF1) and PARP1, respectively, that have been identified in recent proteomic studies.^27^ We aimed to assemble the peptides by a late-stage click reaction between the fully deprotected propargyl-glycine containing peptides and the α- and β-ADP-ribosyl azides **22** and **25**, which in turn can be obtained from the two anomeric azido ribose 5-phosphates (**9** and **17**) and known adenosine phosphoramidite **20** using our P(III)–P(V) coupling method.^28^

Synthesis of the β-configured 5-phosphorylribofuranoside **9** started with commercially available ribofuranose tetraacetate (Scheme 1). Owing to neighboring group participation of the C-2-*O*-acetyl, the desired β-azide was acquired with excellent stereoselectivity.^29^ After a series of standard protecting group manipulations, the fully protected azidoribofuranoside **7** was obtained, of which the silyl ether was selectively removed under acidic conditions. Phosphitylation with bis(fluorenylmethyl)-*N*,*N*-diisopropyl phosphoramidite under influence of pyridine-1-ium chloride as an activator provided the corresponding phosphite which was oxidized with *t*-BuOOH to yield 1-β-azido-5-phosphorylribofuranoside **9**.

**Scheme 1:**
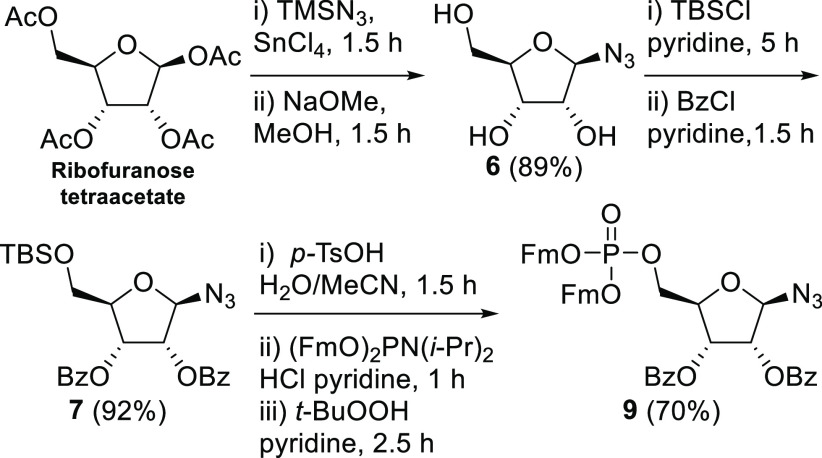
Synthesis of the β-Configured 1-Azido-5-phosphorylribofuranoside **9**

The assembly of the α-configured 1-azido-5-phosphorylribofuranoside requires a nonparticipating protection group at the C-2-hydroxyl to stereoselectively introduce the α-azido group. To this end, suitably protected imidate **14** was prepared from d-ribose in 5 steps (Scheme 2).^30^ Activation of the ribosyl donor **14** with trimethylsilyl triflate at −60 °C in the presence of trimethylsilyl azide provided ribosyl azide **15** with excellent stereoselectivity (α/β = 14:1, Scheme 1B). Removal of the PMB protecting groups proved difficult at this point using either oxidative or acidic conditions, which resulted in the formation of 2,3-*O*-*p*-methoxybenzylidene products or degradation of the compound, respectively. We therefore decided to postpone the removal of the PMB ethers to a later stage and first removed the C-5-*O*-silyl group in **15** with HF-pyridine as a fluorine source. Then, phosphitylation and subsequent oxidation, as described above for β-ribosyl azide **9**, provided α-azido-5-phosphorylribofuranoside **17**.

**Scheme 2:**
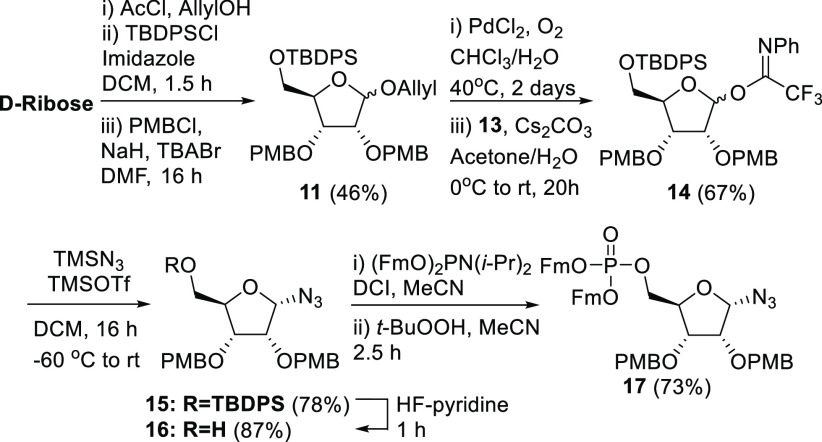
Synthesis of the α-Configured 1-Azido-5-phosphorylribofuranoside **17**

The pyrophosphate linkage in the target α- and β-azido ADP-ribose building blocks was installed using our P(III)–P(V) coupling method (Scheme 3).^28^ The required adenosine phosphoramidite **20** was synthesized from adenosine in 6 steps according to a previously reported method (Scheme S3).^31^ First the phosphates in **9** and **17** were liberated by removal of the Fm-groups with triethylamine. Next, the phosphates **21** and **23** were coupled with adenosine amidite **20** upon activation with dicyanoimidazole (DCI). Subsequent *t*-BuOOH mediated oxidation of the P(III)–P(V) intermediate provided the partially protected pyrophosphates. Deprotection of these building blocks started with the removal of the cyanoethyl groups with DBU, after which treatment with aqueous ammonia provided the fully deprotected β-azido-ADPr **22** and α-azido-ADPr **24**, carrying the two PMB ethers. β-Azido-ADPr **22** could be purified by size exclusion chromatography (SEC) and was isolated as the ammonium salt. On the contrary, due to hydrophobic interactions of the C-2- and C-3-*O*-PMB groups of α-azido-ADPr **25**, SEC was not efficient, and preparative reversed-phase high-performance liquid chromatography (RP-HPLC) was required to obtain the pure compound. Final removal of the PMB groups was executed using a catalytic amount of HCl in hexafluoro-2-propanol^32^ to yield **25** as triethylammonium salt after workup and lyophilization.

**Scheme 3:**
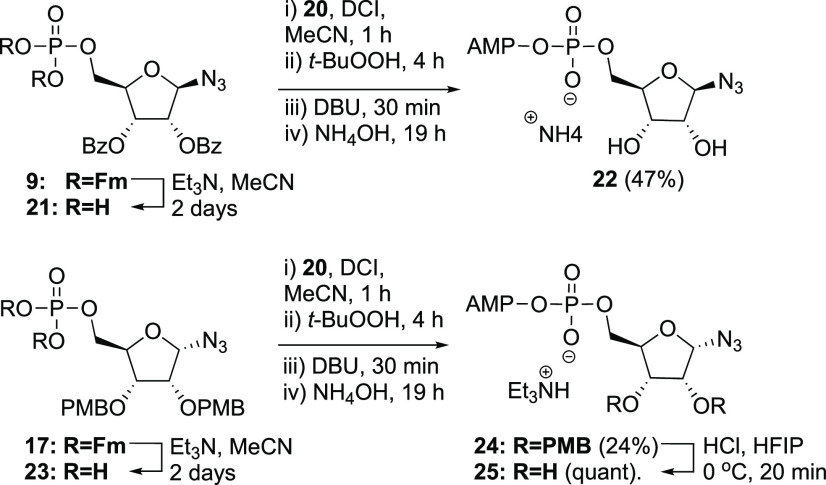
Synthesis of the 1′-Azide ADPr Analogues **22** and **25** Using Phosphoramidite Chemistry

The required peptides **26** and **27** were synthesized using Fmoc-based solid phase peptide synthesis (SPPS), incorporating propargyl glycine at the positions that are to carry the His-type ADPr modification. Both peptides were obtained in good yield and purity after RP-HPLC purification using an NH_4_OAC buffered eluent system.

For the final Cu(I)-catalyzed conjugation, a 1.5-fold molar excess of the azido-ADPr analogue (**22** or **25**) was added to an aqueous solution of the oligopeptide (**26** or **27**) after which the solution was degassed with argon (Scheme 4). In parallel, a fresh “click mixture” was prepared for every reaction by adding an aqueous solution of sodium ascorbate to CuSO_4_ directly followed by tris(3-hydroxypropyltriazolylmethyl)amine (THPTA). After addition of this mixture to the solution of the azide and the alkyne, the conversion of the oligopeptide was monitored with liquid chromatography–mass spectrometry (LC-MS). Upon complete conversion, the crude products were desalted by SEC and subsequently purified by preparative HPLC. Unfortunately, this tandem purification method provided the desired products in moderate yields. Direct preparative RP-HPLC proved to be more efficient and provided the desired products in high purity.

**Scheme 4:**
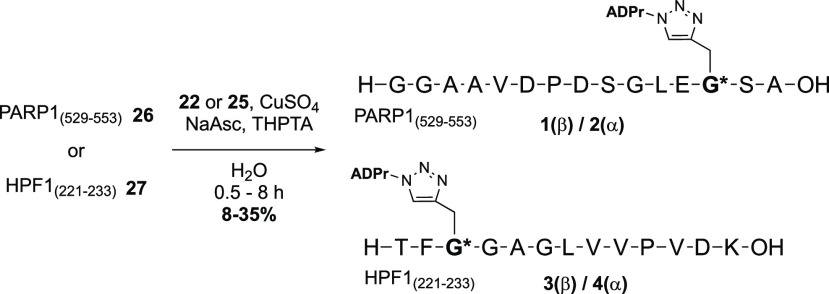
Synthesis of ADP-Ribosylated Histidine Mimetics via Copper(I) Click Chemistry Two oligopeptides originating from PARP1 (**26**) and HPF1 (**27**) have been selected and synthesized with a propargylglycine incorporated at the location of the histidine. Depending on the ADPr analogue used, an α- or β-configured conjugate is formed. Hence, a total of four ADPr conjugates **1–4** are obtained.

Having obtained the triazole mimetics of ADP-ribosylated histidine peptides, we set out to investigate the enzymatic turnover of this modification (Figure 2A). Peptides **1–4** were incubated in the presence of different human (ADP-ribosyl)hydrolases and nudix hydrolase 5 (NudT5) for 1 h at 30 °C. The former may catalyze the breakage of the *N*-glycosidic bond of the ribosyltriazole, while the latter converts the released ADPr into adenosine monophosphate (AMP), which was quantified using the AMP-Glo assay.^33^ As a positive control, the samples were incubated in the presence of NudT16, which in contrast to NudT5 can hydrolyze ADPr that is conjugated to a peptide.^34^ Although most human hydrolases were unable to remove ADPr from the peptides, we observed a consistent minor turnover (∼8.2%) for the HPF1-α peptide **4**, indicating that our developed isostere is indeed a functional mimic of ADP-ribosylated histidine. Interestingly, ARH3 appeared unable to convert PARP1-α (**2**), which could suggest that the removal of His-ADPr modifications is sequence-dependent.^35^ These findings were substantiated in a time-course experiment (Figure 2B), which showed the steady enzymatic conversion of **4** and the resistance of **2** toward enzymatic turnover.

**Figure 2:**
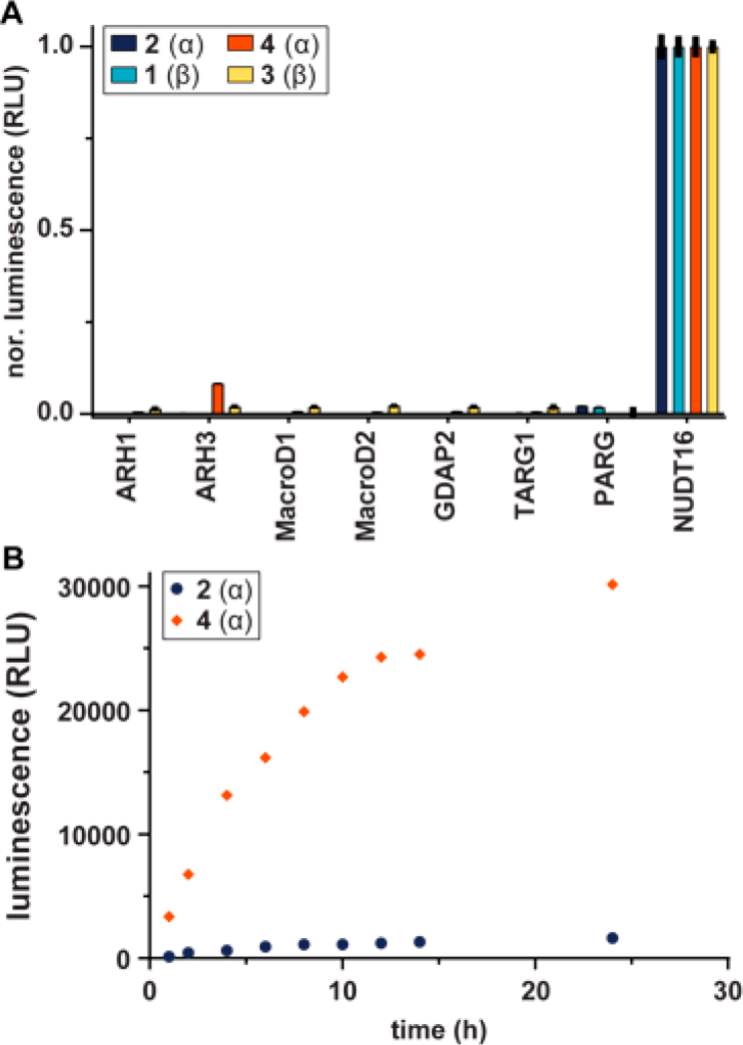
Enzymatic hydrolysis of interglycosidic linkages in ADP-ribosylated histidine mimetic peptides **2(α)**, **1(β)**, **4(α)**, and **3(β)**. (A) Hydrolase activity against the various peptides was assessed by monitoring AMP release directly (NudT16) or converting released ADPr via NudT5 to AMP. AMP was measured using the AMP-Glo assay (Promega). Samples are background corrected and normalized to NudT16 activity. (B) Time-course experiment of histidine isosteres **2(α)** and **4(α)** with ARH3. Time points taken up to 24 h. Data of both experiments represent mean values ± SD measured in triplicates.

In conclusion, we have described the synthesis of both α- and β-configured ADPr-azide analogues that were successfully used to prepare mimetics of ADP-ribosylated histidine using CuAAC. Initial screening of the peptides against a collection of human ADPr hydrolases revealed that ARH3 is able to hydrolyze the N-glycosidic triazole-ribose linkage of HPF1-α **4**, while PARP1-α **2** remained unscathed. Not only do these results suggest that ARH3 is likely able to remove the ADPr modification from histidine residues in the right sequential context,^35^ but it also demonstrates that the peptides presented here provide useful tools for the further study of the interactions of the His-ADPr modification with either binders or hydrolases. Our current efforts in the synthesis of peptides with native ADP-ribosylated histidine will hopefully further elucidate the process of His-ADPr demodification in the near future.

## Supporting Information Available

Syntheses of β-configured ribose 5-phosphate **9**, α-configured ribose 5-phosphate **17**, and phosphoramidite **20**; procedures for pyrophosphate construction, final CuAAC conjugation, solid-phase peptide synthesis of **26** and **27**, plasmid expression, protein purification, and (ADP-ribosyl)hydrolase activity screening; general experimental procedures; and copies of IR and NMR spectra (PDF)
