# Supplementary material for: Mimetics of ADP-Ribosylated Histidine through Copper(I)-Catalyzed Click Chemistry
Source: Org Lett. 2022 May 19;24(21):3776–80. doi: 10.1021/acs.orglett.2c01300 (PMC9171823; doi:10.1021/acs.orglett.2c01300)
Supplement: Supplementary file 1 — ol2c01300_si_001.pdf [file ol2c01300_si_001.pdf]

Supporting information

of

Mimetics of ADP-ribosylated histidine through copper(I)-catalysed click chemistry

Hugo Minnee[1], Johannes G. M. Rack[2], Gijsbert A. van der Marel[1], Herman S. Overkleeft[1], Jeroen D.C. Codée[1], Ivan Ahel[2] and Dmitri V. Filippov[1]*

[1]Bio-Organic Synthesis, Leiden Institute of Chemistry,
Leiden University, P.O. Box 9502, 2300 RA Leiden, The Netherlands
[2]Sir William Dunn School of Pathology,
University of Oxford, South Parks Road, Oxford, OX1 3RE, UK

E-mail: filippov@chem.leidenuniv.nl

Experimental Procedures

Expression plasmids and protein purification

The construction of the expression plasmids and the purification procedures were described earlier.[1–4] Briefly, expression plasmids were transferred into Rossetta (DE3) cells and grown at 37 °C to an OD_{600} of 0.6 in LB medium supplemented with 1% (w/v) D-glucose and appropriate antibiotics. For (ADP-ribosyl)hydrolases (ARH1 and ARH3) the medium was further enriched by addition of 2 mM $MgSO_4$. Expression was induced with 0.4 mM isopropyl β-D-1- thioglactopyranoside (IPTG) and cultured were allowed to grow overnight at 17 °C. Cultures were harvested by centrifugation, pellets resuspended in lysis buffer (50 mM TrisHCl [pH 8], 500 mM NaCl and 25 mM imidazole) and stored at -20 °C until use. Proteins were purified by Ni^{2+}-NTA chromatography (Jena Bioscience) according to the manufacturer's protocol using the following buffers: all buffers contained 50 mM TrisHCl (pH 8) and 500 mM NaCl; additionally, the lysis buffer contained 25 mM, the washing buffer 40 mM, and the elution buffer 500 mM imidazole. Proteins were dialyzed overnight against 50 mM TrisHCl (pH 8), 200 mM NaCl, 1 mM dithiothreitol and 5% (v/v) glycerol and stored at -80 °C. For the purification of ARH1 and ARH3 all purification buffers were additionally supplemented with 10 mM $MgCl_2$.

(ADP-ribosyl)hydrolase activity assay

The peptide demodification assay was described earlier.[5,6] Briefly, peptide concentrations for the assay were estimated using absorbance at λ_{260nm} using the molar extinction coefficient of ADP-ribose (15,400 M^{-1} cm^{-1}). 10 µM indicated peptide were demodified by incubation with 0.5 µM hydrolase for 60 min at 30 °C in assay buffer (50 mM TrisHCl [pH 8], 200 mM NaCl, 10 mM MgCl2, 1 mM dithiothreitol and 0.2 µM human NUDT5)[5]. Reactions were stopped and analysed by performing the AMP-Glo™ assay (Promega) according to the manufacturer's protocol. Luminescence was recorded on a SpectraMax M5 plate reader (Molecular Devices) and data analysed with GraphPad Prism 7. Control reactions were carried out in absence of peptide.

Chemical Synthesis:

All chemicals were used as received unless stated otherwise. $SnCl_4$ (1 M in DCM) and *t*-BuOOH (5.5 M in nonane) were purchased at Sigma Aldrich. Dowex 50WX8 hydrogen form (100-200 mesh) was purchased at Sigma Aldrich and washed with H_2SO_4 (5 M, 3x) and MeOH (3x) prior to use. Molecular sieves were flamedried (3x) *in vacuo* before use. Solvents were dried over activated 4Å molsieves for 24 h except for MeCN and MeOH which were dried over 3Å molsieves. A solution of HCl (0.2 M in HFIP) was freshly prepared every time by dissolving HCl (37%, 0.1 ml) to HFIP (5.9 ml). Reactions were performed under N_2 atmosphere unless stated otherwise. A Julabo FT902 cryostat was used for low temperature glycosylation reactions. Reaction mixtures were concentrated under reduced pressure using rotary evaporators at 40-45 ˚C unless state otherwise. Reactions were monitored by thin layer chromatography (TLC) analysis using silica gel 60 F254 coated aluminum sheets from Merck. TLC plates were visualized with ultraviolet light (254 nm) or sprayed with H_2SO_4 (20% v/v in MeOH), potassium permanganate (1 g $KMnO_4$, 5 g K_2CO_3, in 200 ml H_2O) or ceric ammonium molybdate (1 g $Ce(NH_4)_4(SO_4)_4 \bullet 2H_2O$, 2.5 g $(NH_4)_6Mo_7O_{24} \bullet 4H_2O$, 10 ml H_2SO_4 in 90 ml H_2O). Infrared (IR) values are reported in cm^{-1}. ^1H NMR, ^{13}C NMR and ^{31}P NMR spectra were recorded on Bruker AV-300 (300 mHz), AV-400 (400 mHz) or AV-500 (500 mHz) spectrometer. ^{13}C NMR spectra are acquired via the attached proton test (APT) experiment and are presented with even signals

(C_q and CH_2) pointing upwards and odd signals (CH and CH_3) pointing downwards. The chemical shifts are noted as δ-values in parts per million (ppm) relative to the tetramethylsilane signal (δ = 0 ppm) or solvent signal of D_2O (δ = 4.79 ppm) for ^1H NMR and relative to the solvent signal of $CDCl_3$ (δ = 77.16 ppm) for ^{13}C NMR. Phosphorylation reactions were monitored with ^{31}P NMR using an acetone-D_6 insert for a locking signal and the resulting spectra were indirectly calibrated with H_3PO_4. HRMS samples were prepared in either MeOH, MeCN or milliQ grade water with an approximate concentration of 1 mM and measured on a Thermo Scientific LTQ Orbitrap XL.

Solid phase peptide synthesis:

Fmoc-L-propargyl glycine (cat# 05138) was purchased at Chem-Impex International. Fmoc-Asp(OEpe)-OH, Fmoc-Thr(*t*Bu)-OH, Fmoc-Val-OH, Fmoc-Pro-OH, Fmoc-Leu-OH, Fmoc-Gly-OH, Fmoc-Ala-OH and Fmoc-Phe-OH were all obtained from Merck Novabiochem. Lysine(Boc) was purchased pre-loaded on tentagel S AC resin from RAPP Polymere GmbH. Alanine was loaded manually on tentagel S AC resin from the same manufacturer.

Oligopeptides were prepared using a Liberty Blue peptide synthesizer using 9-fluorenylmethoxycarbonyl (Fmoc) based solid phase peptide chemistry at a 100 µmol scale. A 5 fold excess of the amino acids, with exception of 4 equivalents for Fmoc-L-propargyl glycine, relative to the resin loaded amino acid was added in each prolongation step. A total of 5 equivalents of the additives Diisopropylcarbodiimide (DIC) and OxymaPure were added simultaneously. The coupling was established in the microwave reaction chamber at 90 °C for 2.5 minutes. After each coupling the peptide was subjected three consecutive times to a 20 v/v% piperidine solution in DMF at 90 °C for 1 minute to remove the Fmoc protection group. The oligopeptide was deprotected and simultaneously cleaved by treating it with a TFA/TIS/H_2O (95:2.5:2.5, 7 ml) mixture in a reaction syringe for 2 h at rt. Precipitation in 8 volumes of ice-cold Et_2O followed by centrifugation (5 min) yielded the crude residue that was purified by preparative HPLC.

Silica 60 M (0.04-0.063 mm) from Macherey-Nagel GmbH was used in combination with solvents of technical grade from Sigma Aldrich for silica gel column chromatography. Size exclusion chromatography (SEC) was performed by constant elution (1 ml/min) with an aqueous NH_4OAc (0.15 M) + 10% MeCN buffer system over an HW-40-S resin (16x 600 mm) from TOYOPEARL. Purification by preparative high pressure liquid chromatography (HPLC) was carried out on a Gilson-preparative-system equipped with a Phenomenex-Gemini-NX C18 column (5µm, 10x250 mm) using Buffer A (25 mM NH_4OAc in water) and Buffer B (MeCN). Yields for the ADPr analogues after size exclusion chromatography were calculated assuming its obtained as NH_4 salt and corrected for residual salts like NH_4OBz and NH_4OAc using NMR analysis.

Figure S1. Synthesis of 1-β-azido-5-phosphorylribofuranoside **9**.

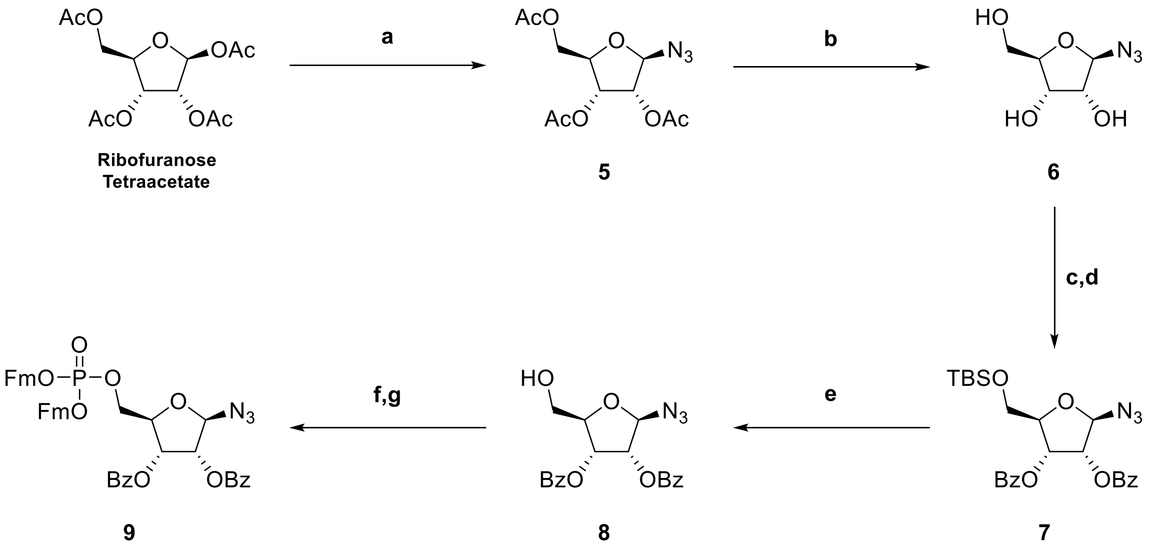

Reagents and conditions: **a)** $TMSN_3$, $SnCl_4$, DCM, rt, 1.5 h (97%). **b)** NaOMe, MeOH, rt, 1.5 h (92%). **c)** TBSCl, pyridine, rt, 5 h. **d)** BzCl, pyridine, rt, 1.5 h (92% over 2 steps). **e)** *p*-TsOH•H_2O, H_2O/MeCN (1:1), rt, 1.5 h (92%). **f)** $(FmO)_2PN(i\text{-}Pr)_2$, pyridine-1-ium chloride, pyridine, rt, 1 h. **g**) *t*-BuOOH, pyridine, rt, 2.5 h (76% over 2 steps).

1-β-Azido-2,3,5-tris-*O*-acetyl-D-ribofuranoside (5).

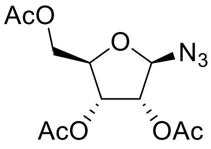

β-D-Ribofuranose 1,2,3,5-tetraacetate (2.0 g, 2.7 mmol) and azidotrimethylsilane (1.08 ml, 8.17 mmol) were dissolved in anhydrous DCM (20 ml) and a solution of $SnCl_4$ (50 mM, 6 ml, 0.3 mmol) in anhydrous DCM was added dropwise to the reaction mixture. After stirring for 1.5 h at rt, $NaHCO_3$ (sat., 10 ml) was added and the biphasic system was vigorously stirred for 10 min. The layers were separated and the water fraction was extracted with DCM (2x 15 ml). The combined organic fractions were dried over $MgSO_4$, filtered and concentrated under reduced pressure. Purification of the crude residue by silica gel column chromatography (Pentane/EtOAc = 80:20 → 70:30) yielded title compound **5** (1.84 g, 6.28 mmol, 97%) as clear oil. $\mathbf{R_f}$ = 0.2 (pentane/EtOAc = 80:20). The obtained spectra were in full accordance with literature experimental data.[7]

1-β-Azido-D-ribofuranoside (6).

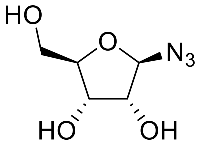

Compound **5** (1.61 g, 5.34 mmol) was co-evaporated with anhydrous dioxane (3x), dissolved in anhydrous MeOH (10 ml) and cooled to 0 ˚C. NaOMe (1.16 g, 21.4 mmol) was added in one go and the reaction mixture was warmed to room temperature after 10 min. The suspension was vigorously stirred at rt for 1 h and quenched with Amberlite (H+) resin. After filtration the crude residue was washed with additional MeOH and the filtrate was concentrated under reduced pressure. Purification of the crude residue by silica gel column chromatography (DCM/MeOH = 95:5 → 90:10) provided title compound **6** (886 mg, 5.34 mmol, 95%) as a clear oil. $\mathbf{R_f}$ = 0.4 (DCM/MeOH = 90:10). The obtained spectra were in full accordance with literature experimental data.[8]

1-β-Azido-2,3-bis-*O*-benzoyl-5-*O*-*tert*-butyldimethylsilyl-D-ribofuranoside (7).

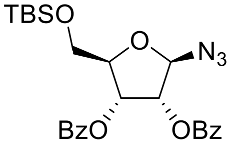

Compound **6** (748 mg, 4.27 mmol) was co-evaporated with anhydrous pyridine (3x) before dissolving in anhydrous pyridine (21 ml). TBSCl (2.4 ml, 6.8 mmol) was added and the reaction was stirred for 1.5 h at rt. BzCl (1.7 ml, 15 mmol) was added and the resulting suspension was quenched with H_2O (0.5 ml) after 40 min. The solution was concentrated under reduced pressure and subsequent purification of the crude residue by silica gel column chromatography (pentane/Et_2O = 97.5:2.5 → 95:5) resulted in title compound **7** (1.99 g, 4.27 mmol, 94%) as a clear oil. $\mathbf{R_f}$ = 0.5 (pentane/Et_2O = 90:10). **^{1}H NMR** (400 MHz, $CDCl_3$): δ 8.02 – 7.90 (m, 4H), 7.60 – 7.49 (m, 2H), 7.44 – 7.32 (m, 4H), 5.76 (t, *J* = 5.1 Hz, 1H), 5.60 (d, *J* = 3.2 Hz, 1H), 5.53 (dd, *J* = 5.1, 3.2 Hz, 1H), 4.49 (dt, *J* = 5.2, 3.4 Hz, 1H), 4.00 – 3.88 (m, 2H), 0.95 (s, 9H), 0.15 (d, *J* = 1.0 Hz, 6H). **^{13}C NMR** (101 MHz, $CDCl_3$): δ -5.5, -5.4, 18.4, 26.0, 63.2, 72.0, 75.5, 83.6, 93.0, 128.5, 128.5, 129.1, 129.2, 129.8, 129.9, 133.5, 133.6, 165.2, 165.4. **IR**: 2126, 1737, 1269 cm^{-1}. **HRMS** (ESI) m/z: $[M + Na]^+$ calculated for $C_{25}H_{31}N_3O_6SiNa$ 520.1874; Found 520.1877. $[M + NH_4]^+$ calculated for $C_{25}H_{31}N_3O_6SiNH_4$ 515.2320; Found, 515.2324.

1-β-Azido-2,3-bis-*O*-benzoyl-D-ribofuranoside (8).

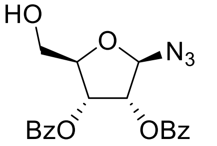

To a solution of compound **7** (1.8 g, 3.6 mmol) in MeCN/H_2O (7:1, 21 ml) was added *p*-TsOH•H_2O (1.0 g, 5.4 mmol). After stirring for 1 h at rt, the reaction was quenched with $NaHCO_3$ (sat., 6 ml) and diluted with EtOAc (150 ml). The organic fraction was washed with H_2O (3x 30 ml) and subsequently dried, filtered and concentrated under reduced pressure. Purification of the crude residue by silica gel column chromatography (pentane/EtOAc = 90:10 → 80:20) yielded title compound **8** (1.27 g, 3.61 mmol, 92%) as a clear oil. $\mathbf{R_f}$ = 0.5 (pentane/EtOAc = 70:30). **^{1}H NMR** (400 MHz, $CDCl_3$): δ 8.05 – 7.97 (m, 2H), 7.95 – 7.87 (m, 2H), 7.62 – 7.49 (m, 2H), 7.46 – 7.30 (m, 4H), 5.71 (dd, J = 6.3, 5.1 Hz, 1H), 5.64 (d, J = 2.2 Hz, 1H), 5.54 (dd, J = 5.0, 2.2 Hz, 1H), 4.50 (ddd, J = 6.2, 4.0, 3.1 Hz, 1H), 4.02 (dd, J = 12.4, 3.2 Hz, 1H), 3.87 (dd, J = 12.4, 4.1 Hz, 1H), 2.45 (bs, 1H). **^{13}C NMR** (101 MHz, $CDCl_3$): δ 62.5, 71.3, 75.9, 83.3, 93.4, 128.6, 128.6, 128.9, 128.9, 129.8, 129.9, 133.7, 133.8, 165.2, 165.7. **IR**: 2113.5, 1717.1, 1269.3, 1093.2, 685.4 cm^{-1}. **HRMS** (ESI) m/z: $[M + Na]^+$ calculated for $C_{19}H_{17}N_3O_6Na$ 406.1010; Found 406.1012. $[M + NH_4]^+$ calculated for $C_{19}H_{17}N_3O_6NH_4$ 401.1456; Found 406.1459.

1-β-Azido-2,3-bis-*O*-benzoyl-5-*O*-(di(9H-fluoren-9-yl))-phosphoryl-D-ribofuranoside (9).

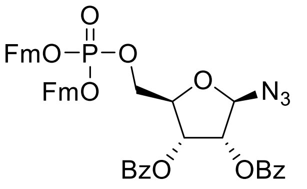

Compound **8** (200 mg, 0.52 mmol) and pyridine-1-ium chloride (241 mg, 2.09 mmol) were co-evaporated with anhydrous toluene (2x) and anhydrous pyridine (1x) before dissolving in anhydrous pyridine (4 ml). A solution of bis((9H-fluoren-9-yl)methyl)diisopropylphosphoramidite (408 mg, 0.783 mmol) in anhydrous pyridine (1.5 ml) was added dropwise to the reaction mixture. After stirring for 1 h at rt, *t*-BuOOH (5.5 M, 0.7 ml) was added and the reaction was monitored by ^{31}P NMR until no residual phosphotriester signal (~125 ppm) was observed. Upon completion the reaction was diluted with DCM (15 ml) and washed with $NaHCO_3$ (sat., 30 ml). The layers were separated and the H_2O fraction was extracted with DCM (2x 15 ml). The combined organic fractions were dried over $MgSO_4$, filtered and concentrated under reduced pressure. Purification of the crude residue by silica gel column chromatography (pentane/EtOAc = 70:30 → 60:40) provided title compound **9** (325 mg, 0.522 mmol, 76%) as a white foam. $\mathbf{R_f}$ = 0.6 (pentane/EtOAc = 50:50). **^{1}H NMR** (400 MHz, $CDCl_3$): δ 7.96 (dd, J = 8.4, 1.3 Hz, 2H), 7.86 (dd, J = 8.3, 1.4 Hz, 2H), 7.76 – 7.65 (m, 4H), 7.62 – 7.48

(m, 6H), 7.46 – 7.29 (m, 8H), 7.30 – 7.19 (m, 6H), 5.63 (dd, J = 6.7, 4.9 Hz, 1H), 5.60 (d, J = 1.9 Hz, 1H), 5.45 (dd, J = 5.0, 1.9 Hz, 1H), 4.38 – 4.28 (m, 4H), 4.25 – 4.08 (m, 4H). **^{13}C NMR** (101 MHz, $CDCl_3$): δ 48.0, 48.0, 48.1, 48.1, 66.7, 69.6, 69.6, 69.6, 69.7, 71.2, 75.3, 80.6, 80.7, 93.4, 120.1, 120.1, 125.3, 125.3, 127.3, 127.3, 128.0, 128.0, 128.6, 128.7, 128.8, 128.9, 129.9, 130.0, 133.8, 133.9, 141.5, 141.5, 141.5, 143.2, 143.2, 143.2, 165.1, 165.2. **^{31}P NMR** (162 MHz, CDCl3): δ -2.52. **IR**: 2115, 1729, 1264, 1243, 989, 734. **HRMS** (ESI) m/z: $[M + Na]^+$ calculated for $C_{47}H_{38}N_3O_9PNa$ 842.2238; Found 837.2238. $[M + NH_4]^+$ calculated for $C_{47}H_{38}N_3O_9P$ NH_4 837.2684; Found 837.2682.

Figure S2. Synthesis of α-azido-5-phosphorylribofuranoside **17**.

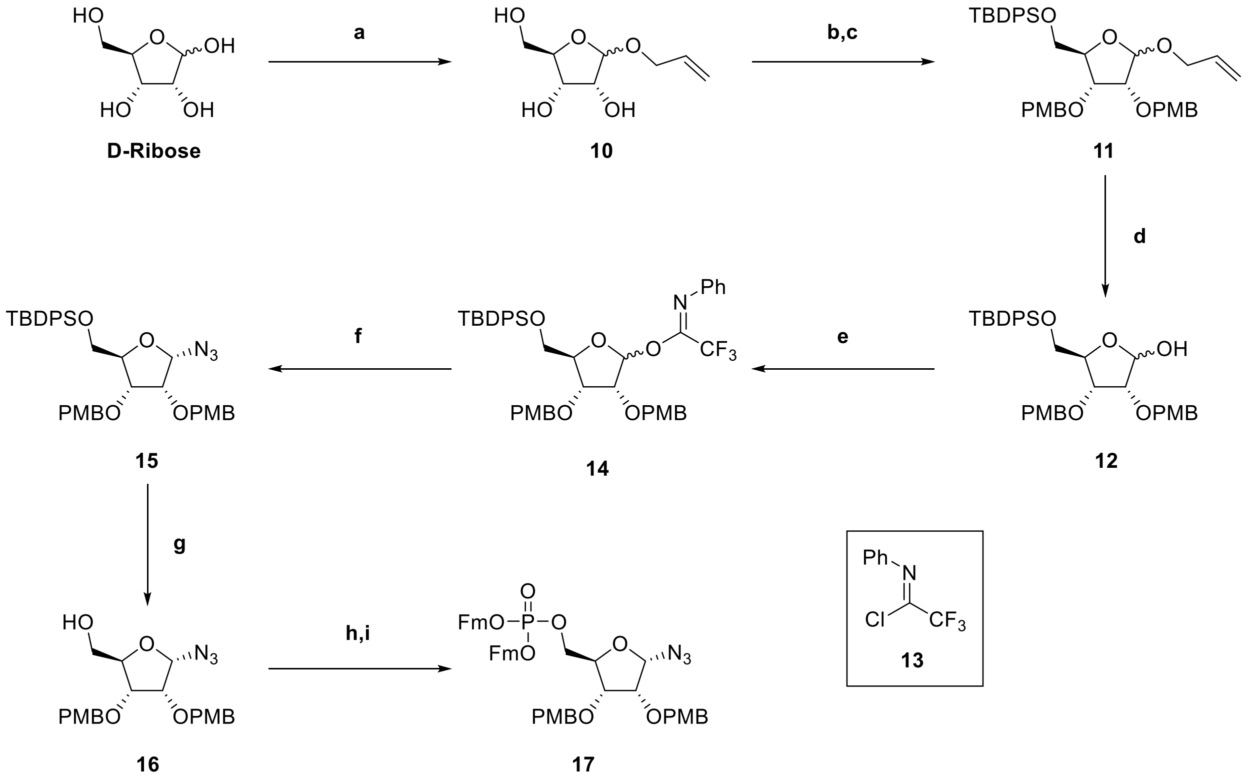

Reagents and conditions: **a)** AcCl, allyl alcohol, rt, 1 h (91%, α:β = 3:1). **b)** TBDPSCl, imidazole, DCM, rt, 1.5 h. **c)** PMBCl, NaH, TBABr, DMF, rt, 16 h (49% over 2 steps). **d)** $PdCl_2$, O_2, $CHCl_3/H_2O$ (3:1), 40 ˚C, 2 days (73%). **e) 13**, Cs_2CO_3, acetone/H_2O (30:1), 0 ˚C to rt, 20 h (92%) **f)** $TMSN_3$, TMSOTf, DCM, -60 ˚C to rt, 16 h (78%, α:β = 14:1). **g)** HF-pyridine, pyridine, rt, 1 h (87%). **h)** $(FmO)_2PN(i\text{-}Pr)_2$, DCI, MeCN, rt, 2 h. **i)** *t*-BuOOH, MeCN, rt, 1.5 h (73% over 2 steps).

1-α,β-*O*-Allyl-D-ribofuranoside (10).

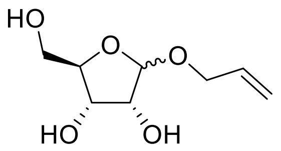

To a vigorously stirring suspension of D-ribose (7.88 g, 52.5 mmol) in allyl alcohol (130 ml) was added AcCl (2.61 ml, 36.7 mmol). After 1 h, the reaction was quenched with pyridine (7 ml) and concentrated under reduced pressure. Purification by silica gel column chromatography (DCM/acetone = 50:50) yielded an anomeric mixture of title compound **10** (8.96 g, 47.2 mmol, 90%, α:β =1:3) as a clear oil. **R_f** = 0.6 (DCM/MeOH = 80:20). The obtained spectra were in full accordance with literature experimental data.[9]

1-α,β-Allyl-2,3-bis-*O*-(4-methoxybenzyl)-5-*O*-*tert*-butyldiphenylsilane-D-ribofuranoside (11).

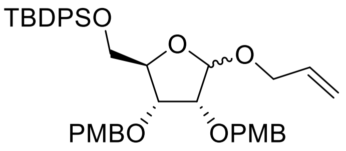

Compound **10** (8.96 g, 47.2 mmol) was co-evaporated with anhydrous toluene (2x) before dissolving in anhydrous DCM (160 ml). Imidazole (4.9 g, 72 mmol) and TBDPSCl (17.7 ml, 68.2 mmol) were added consecutively and the resulting white mixture was stirred at rt for 1.5 h. The reaction was diluted with H_2O (150 ml) and brine (50 ml) and extracted with DCM (150 ml). The combined organic fractions were dried over $MgSO_4$, filtered and concentrated under reduced pressure. The crude residue was co-evaporated with anhydrous toluene (3x) before dissolving in anhydrous DMF (140 ml). PMBCl (16.2 ml, 120 mmol) and a catalytic amount of TBABr were added followed by portionwise addition of NaH (60 wt%, 4.80 g, 120 mmol) 0 ˚C. The reaction mixture was stirred overnight while slowly warming to rt. The resulting yellow suspension was carefully quenched at 0 ˚C with H_2O (3 ml). The solution was diluted with H_2O (300 ml) and extracted with Et_2O (2x 150 ml). The combined organic fractions were dried over $MgSO_4$, filtered and concentrated under reduced pressure. Purification of the crude residue by silica gel column chromatography (pentane/EtOAc = 95:5 → 80:20, 5% steps) provided an anomeric mixture of title compound **11** (15.4 g, 23.0 mmol, 49%, α:β =1:3) as a clear oil. **R_f** = 0.3 for α-anomer, 0.5 for β-anomer (pentane/EtOAc = 85:15). The obtained spectra were in full accordance with literature experimental data.[10]

Bis-2,3-*O*-(4-methoxybenzyl)-5-*O*-*tert*-butyldiphenylsilane-D-ribofuranoside (12).

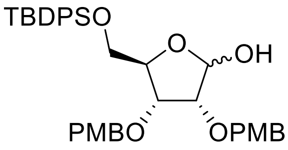

To a vigorously stirring solution of compound **11** (15.4 g, 23.0 mmol) in $CHCl_3/H_2O$ (3:2, 116 ml) was added $PdCl_2$ (816 mg, 4.60 mmol) before heating to 40 ˚C using an oil bath. After bubbling the black mixture with O_2 for 2 days, the suspension was filtered over celite and washed with EtOAc (50 ml). The filtrate was concentrated under reduced pressure and the crude residue was re-dissolved in EtOAc (100 ml). $NaHCO_3$ (sat., 100 ml) and I_2 (6.1 g, 24 mmol) were added in order to cleave the allyl isomer. The organic fraction was washed with NaS_2O_5 (sat., 100 ml). The combined H_2O fractions were back-extracted with EtOAc (100 ml). The combined organic fractions were dried over $MgSO_4$, filtered and concentrated under reduced pressure. Purification of the crude residue by silica gel column chromatography (pentane/EtoAc = 85:15 → 60:40) resulted in an anomeric mixture of title compound **12** (10.6 g, 23 mmol, 73%, α:β = N.D. due to overlapping signals) as a clear oil. **R_f** = 0.2 (pentane/EtOAc = 85:15). The obtained spectra were in full accordance with literature experimental data.[10]

1-*O*-((*N*-Phenyl)-2,2,2-trifluoroacetimido)-2,3-bis-*O*-(4-methoxybenzyl)-5-*O*-*tert*-butyldiphenylsilane-D-ribofuranoside (14).

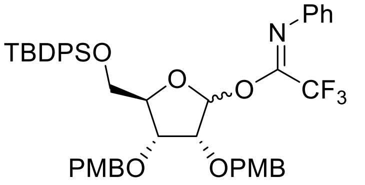

To a solution of Compound **12** (10.6 g, 16.9 mmol) in acetone/H_2O (50:1, 107 ml) at 0 ˚C was added $CsCO_3$ (8.24 g, 25.3 mmol). Acetimidoyl **13** (4.02 g, 19.4 mmol) was added dropwise and the suspension was stirred vigorously for at 0 ˚C. The reaction mixture was filtered and the filtrate was concentrated under reduced pressure. Purification of the crude residue by silica gel column chromatography (pentane/EtOAc + 1v/v% Et_3N = 95:5 → 90:10) yielded an anomeric mixture of title compound **14** (12.4 g, 15.5 mmol, 92%, α:β =1:3) as a yellowish oil. **R_f** = 0.5 (pentane/EtOAc = 80:20). The obtained spectra were in full accordance with literature experimental data.[10]

1-α-Azido-2,3-bis-*O*-(4-methoxybenzyl)-5-*O*-*tert*-butyldiphenylsilane-D-ribofuranoside (15).

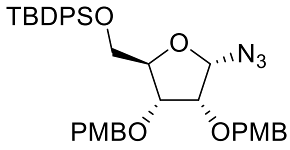

Compound **14** (1.02 g, 1.28 mmol) was co-evaporated with anhydrous toluene (3x) before transferring to a flame dried flask carrying activated 3Å using anhydrous DCM (40 ml). $TMSN_3$ (0.65 ml, 4.9 mmol) was added and the reaction mixture was stirred for 1.5 h at rt before cooling to -60 ˚C. TMSOTf (54 µl, 0.30 mmol) was added in one go and after 20 h, the resulting yellow solution was quenched with Et_3N (0.3 ml). The reaction mixture was concentrated under reduced pressure and the crude residue was subjected to silica gel column chromatography (pentane/Et_2O = 95:5 → 80:20) to provide an anomeric mixture of title compound **15** (654 mg, 1.00 mmol, 78%, α:β =14:1) as a clear oil. $\mathbf{R_f}$ = 0.5 (pentane/EtOAc = 85:15). **^{1}H NMR** (400 MHz, $CDCl_3$): δ 7.63 – 7.56 (m, 4H), 7.49 – 7.36 (m, 6H), 7.32 – 7.23 (m, 4H), 6.91 – 6.83 (m, 4H), 5.23 (d, J = 4.4 Hz, 1H), 4.66 – 4.58 (m, 3H), 4.51 (d, J = 12.1 Hz, 1H), 4.28 (q, J = 2.9 Hz, 1H), 4.05 – 3.98 (m, 2H), 3.81 (s, 6H), 3.68 (dd, 11.4, 3.2 Hz, 1H), 3.55 (dd, 11.3, 2.8 Hz, 1H), 0.98 (s, 9H). **^{13}C NMR** (101 MHz, CDCl3): δ 159.4, 135.7, 135.6, 133.1, 132.9, 130.0, 139.0, 129.8, 129.7, 129.5, 127.9, 127.9, 127.8, 127.8, 114.0, 113.9, 113.9, 90.7, 84.9, 78.3, 75.2, 72.7, 72.3, 63.8, 55.3, 26.9, 26.8, 19.3. **IR**: 2931, 2858, 2836, 2109, 1611, 1511, 1243 cm^{-1}. **HRMS** (ESI) m/z: $[M + Na]^+$ calculated for $C_{37}H_{43}N_3O_6SiNa$ 676.2813; Found 676.2811. $[M + NH_4]^+$ calculated for $C_{37}H_{43}N_3O_6SiNH_4$ 671.3259; Found 671.3256.

1-α-Azido-2,3-bis-*O*-(4-methoxybenzyl)-D-ribofuranoside (16).

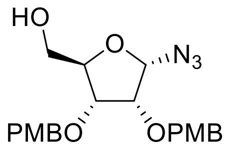

Compound **15** (1.45 g, 2.22 mmol) was co-evaporated with anhydrous toluene (2x) before dissolving in anhydrous pyridine (35 ml). HF-pyridine (3.20 ml, 24.9 mmol) was added and the solution was stirred at rt for 3.5 h. $NaHCO_3$ (sat., 70 ml) was added and the H_2O fraction was extracted with DCM (2x 75 ml). The combined organic fractions were dried over $MgSO_4$, filtered and concentrated under reduced pressure. Purification of the crude residue by silica gel column chromatography (pentane/EtOAc = 90:10 → 60:40) yielded title compound **16** (805 mg, 1.94 mmol, 87%) as a clear oil. $\mathbf{R_f}$ = 0.3 (pentane/EtOAc = 60:40). **^{1}H NMR** (400 MHz, $CDCl_3$): δ 7.34 – 7.21 (m, 4H), 6.88 (dd, *J* = 8.5, 6.3 Hz, 4H), 5.14 (dd, J = 3.2, 1.6 Hz, 1H), 4.66 – 4.56 (m, 3H), 4.46 (d, J = 11.9 Hz, 1H), 4.26 (m, 1H), 3.89 (dd, *J* = 3.1, 1.4 Hz, 2H), 3.80 (d, *J* = 1.8 Hz, 6H), 3.71 (dt, J = 12.3, 3.3 Hz, 1H), 3.50 – 3.40 (bs, 1H). **^{13}C NMR** (101 MHz, $CDCl_3$): δ 55.4, 62.2, 72.5, 72.9, 75.1, 78.1, 84.1, 90.7, 114.0, 114.0, 129.5, 129.8, 129.8, 159.5, 159.6. **IR:** 2109, 1611, 1513, 1243, 1027, 816 cm^{-1}. **HRMS** (ESI) m/z: $[M + Na]^+$ calculated for $C_{21}H_{25}N_3O_6Na$ 438.1636; Found 438.1636. $[M + NH_4]^+$ calculated for $C_{21}H_{25}N_3O_6NH_4$ 433.2082; Found 433.2083.

1-α-Azido-2,3-bis-*O*-(4-methoxybenzyl)-5-(di(9H-fluoren-9-yl))-phosphoryl-D-ribofuranoside (17).

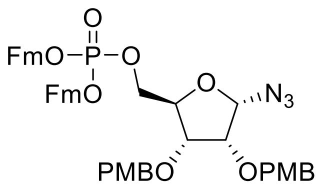

Compound **16** (270 mg, 0.647 mmol) and DCI (229 mg, 1.94 mmol) were co-evaporated with anhydrous toluene (3x) and dissolved in anhydrous MeCN (4.5 ml). A solution of $(FmO)_2PN(iPr)_2$ (507 mg, 0.971 mmol) in anhydrous MeCN (3 ml) was added dropwise and the suspension was stirred at rt under argon atmosphere. After 1.5 h, *t*-BuOOH (5.5 M, 0.59 ml, 3.2 mmol) was added and the reaction was monitored by ^{31}P NMR until no residual phosphotriester signal (~125 ppm) was observed. Upon completion, the reaction was diluted H_2O (75ml) and extracted with DCM (2x 75 ml). The combined organic fractions were dried over $MgSO_4$, filtered and concentrated under reduced pressure. Purification of the crude residue by silica gel column chromatography (pentane/EtOAc = 80:20 → 50:50, 10% steps) resulted in title compound **17** (402 mg,

0.647 mmol, 73%) as a white foam. **^{1}H NMR** (400 MHz, $CDCl_3$): δ 7.77 – 7.67 (m, 4H), 7.55 – 7.14 (m, 14H), 7.11 (d, J = 8.7 Hz, 2H), 6.79 (dd, J = 20.2, 8.6 Hz, 3H), 4.97 (d, J = 4.0 Hz, 1H), 4.52 – 4.17 (m, 9H), 4.11 (m, 2H), 3.92 – 3.79 (m, 1H), 3.79 – 3.61 (m, 9H). **^{13}C NMR** (101 MHz, $CDCl_3$): δ 47.9, 48.0, 55.4, 55.4, 66.8, 66.9, 69.5, 69.6, 72.7, 73.2, 75.3, 77.7, 81.6, 81.7, 90.5, 114.0, 114.1, 120.2, 120.3, 125.0, 125.1, 127.3, 127.3, 128.2, 128.2, 128.9, 129.1, 129.8, 130.0, 141.5, 142.9, 143.0, 159.8. **^{31}P NMR** (122 MHz, CDCl3): δ -2.33. **IR:** 2111, 1513, 1246, 1012, 757 cm^{-1}. **HRMS** (ESI) m/z: $[M + Na]^+$ calculated for $C_{49}H_{46}N_3O_9PNa$ 874.2864; Found 874.2863. $[M + NH_4]^+$ calculated for $C_{49}H_{46}N_3O_9PNH_4$ 869.331; Found 869.3305.

Figure S3. Synthesis of phosphoramidite nucleoside **20**.

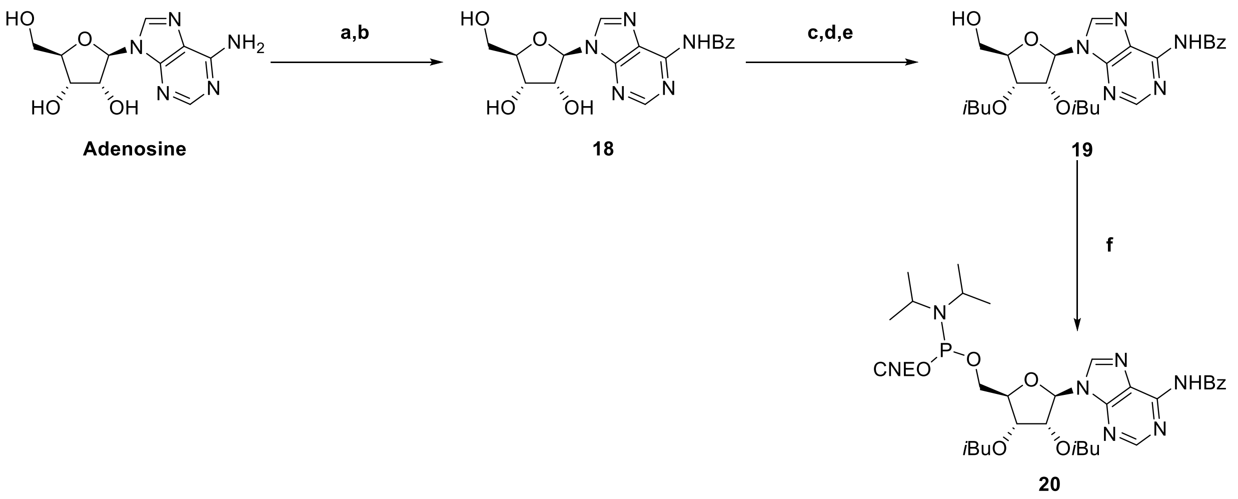

Reagents and conditions: **a)** TMSCl, BzCl, pyridine, 0 ˚C, 3 h. **b)** NH_4OH (28%), pyridine/H_2O (3:2), 0 ˚C, 45 min (quant). **c)** TBSCl, pyridine, rt, 19 h. **d)** *i*-BuO_2, pyridine, rt, 5 h. **e)** *p*-TsOH•H_2O, MeCN/H_2O (4:1), rt, 3 h (38% over 3 steps). **f)** (*i*-Pr)$_2$NPCl(OCNE), DIPEA, DCM, rt, 45min (94%).

6-*N*-benzoyladenosine (18).

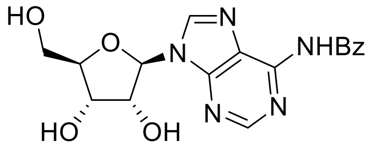

Adenosine (2.00 g, 7.50 mmol) was co-evaporated with anhydrous pyridine (3x) before adding pyridine (38 ml) and TMSCl (9.1 ml, 71 mmol). After stirring vigorously for 15 min at rt, BzCl (4.34 ml, 37.4 mmol) was added to the white suspension. The reaction was stirred for 1.5 h at 0 ˚C and quenched with H_2O (7 ml). NH_4OH (28%, 17 ml) was added and the mixture was stirred at rt for 1 h before concentrating under reduced pressure using a water aspirator. H_2O (110 ml) and EtOAc (40 ml) were added to the crude residue and the resulting suspension was cooled to 0 ˚C before filtration. The precipitate was dried in a vacuum stove at 60 ˚C for several hours to yield title compound **18** (2.8 g, 7.5 mmol, quant.) as a white solid. The obtained spectra were in full accordance with literature experimental data.[11]

6-*N*-benzoyl-2,3-bis-*O*-*iso*-butyryladenosine (19).

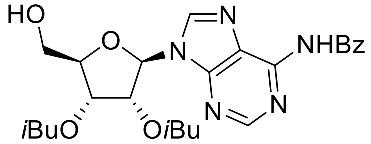

Compound **18** (2.80 g, 7.54 mmol) was co-evaporated with anhydrous pyridine (3x) before dissolving in anhydrous pyridine (3x). TBSCl (3.3 ml, 9.4 mmol) was added in one go and the resulting suspension was stirred for 16 h at rt before *i*-Bu_2O (3.10 ml, 18.9 mmol) was added. After 4.5 h, the reaction was quenched with H_2O (0.8 ml) and concentrated under

reduced pressure. The crude residue was re-dissolved in EtOAc (150 ml) and washed with $NaHCO_3$ (sat., 75 ml), citric acid (1M, 75 ml) and H_2O (75 ml). The organic fraction was dried over $MgSO_4$, filtered and concentrated under reduced pressure. Purification of the crude residue by silica gel column chromatography (DCM/MeOH = 99:1 → 97:3) provided the fully protected intermediate (2.22 g, 3.55 mmol, 47% over 2 steps) as a clear oil along with a mixture of the mono-*i*Bu protected side product (1.32 g, 2.38 mmol, 32% over 2 steps) as a clear oil. The desired intermediate was dissolved in MeCN/H_2O (4:1, 14 ml) and *p*-TsOH•H_2O (1.0 g, 5.3 mmol) was added in one go. After 2.5 h the reaction was quenched with $NaHCO_3$ (sat., 6 ml) and concentrated under reduced pressure. Purification of the crude residue by silica gel column chromatography (DCM/MeOH = 99:1 → 97:3) yielded title compound **19** (1.45 g, 3.50 mmol, 37% over 3 steps) as a white foam. **R_f** = 0.6 (DCM/MeOH = 95:5). The obtained spectra were in full accordance with literature experimental data.[12]

2-Cyanoethoxy-N,N'-diisopropylamino-(6-*N*-benzoyl-2,3-bis-*O*-*iso*-butyryladenyl-5'-yl)phosphine (20).

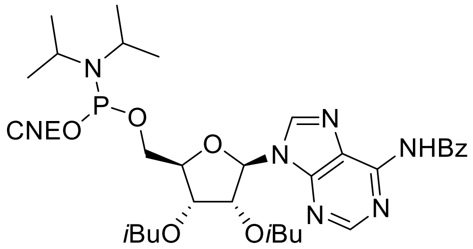

Compound **19** (512 mg, 1.00 mmol) was co-evaporated with anhydrous toluene (3x) before dissolving in anhydrous DCM (5 ml). DIPEA (0.43 ml, 2.5 mmol) and (*i*-Pr)$_2$NPCl(OCNE) (0.25 ml, 1.1 mmol) were added subsequently and the solution was stirred at rt under argon atmosphere. After 1 h, the reaction mixture was diluted with EtOAc (15 ml) and washed with $NaHCO_3$ (sat., 10 ml) and brine (10 ml). The combined H_2O fractions were extracted with EtOAc (2x 10 ml). The combined organic fractions were dried over $MgSO_4$, filtered and concentrated under reduced pressure. Purification of the crude residue by silica gel column chromatography (DCM/MeOH + 1% Et_3N = 95.5:0.5) provided title compound **20** (670 mg, 0.94 mmol, 94%) as a white foam. **R_f** = 0.5 (DCM/MeOH + 1% Et_3N = 97.5:2.5). The obtained spectra were in full accordance with literature experimental data.[12]

Figure S4. Coupling of the 5-phosphorylribofuranosides **9** and **17** with phosphoramidite **20**.

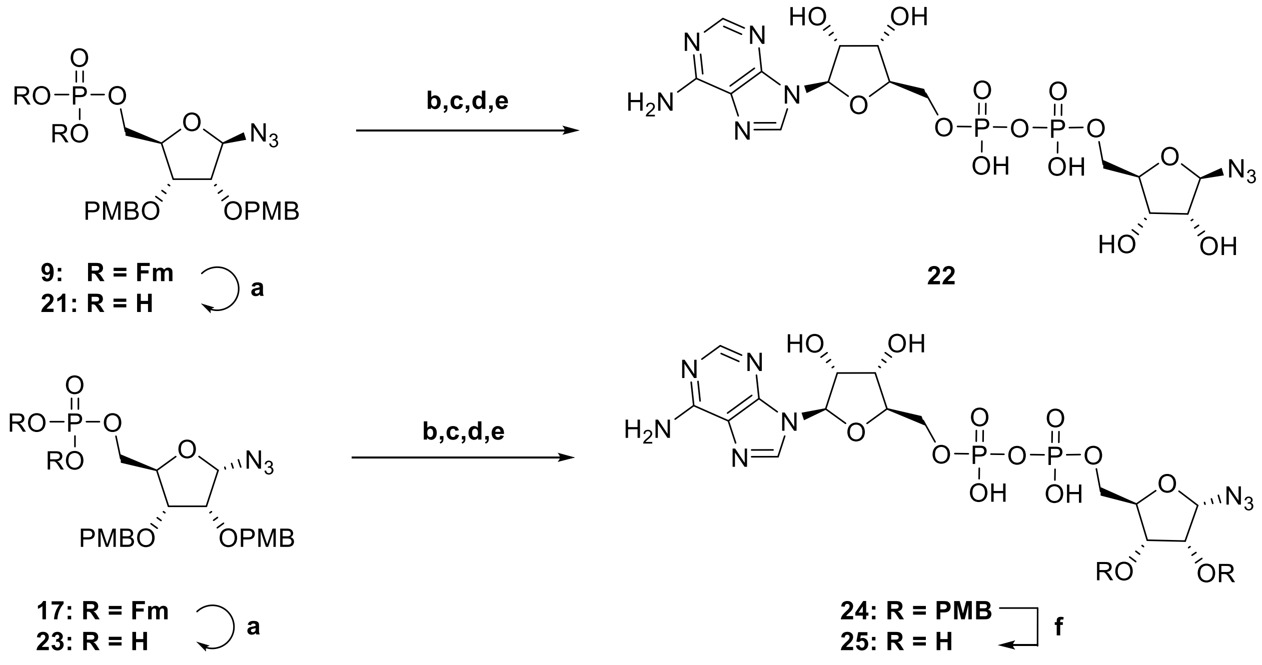

Reagents and conditions: **a)** Et_3N, MeCN, rt, 2-3 days. **b)** **20**, DCI, MeCN, rt, 0.5-1 h. **c)** *t*-BuOOH, MeCN, rt, 2-4 h. **d)** DBU, MeCN, rt, 0.5 h. **e)** NH_4OH (28%)rt, 16-19 h (47% & 24% yield over 5 steps for **22** and **25** respectively). **f)** HCl, HFIP, 0 °C, 20 min (quant).

1-β-Azido-ADPr (22).

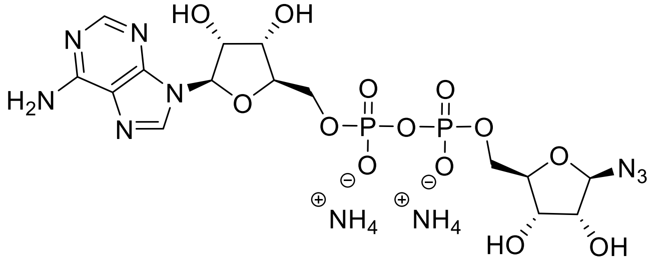

A solution of compound **9** (540 mg, 0.659 mmol) and Et_3N (1.58 ml, 11.3 mmol) in anhydrous MeCN (15 ml) was stirred for 2 days at rt. The solution was concentrated under reduced pressure and the crude residue was co-evaporated with a mixture of anhydrous MeCN/pyridine (1:1, 3x). DCI (191 mg, 1.62 mmol) was added and the mixture was co-evaporated with anhydrous MeCN (3x) before adding anhydrous MeCN (12 ml). Compound **20** (460 mg, 0.646 mmol) was co-evaporated with anhydrous toluene (2x) before adding dropwise to the white suspension using anhydrous MeCN (3 ml). The reaction mixture was vigorously stirred under argon atmosphere for 1 h before adding *t*-BuOOH (5.5 M, 0.25 ml, 1.4 mmol). After 1 h, a second portion of *t*-BuOOH (5.5 M, 0.25 ml, 1.4 mmol) was added. The reaction was monitored by ^{31}P NMR until no residual phosphotriester signals at 125-127 ppm were observed. Upon completion, DBU (0.49 ml, 3.2 mmol) was added and the mixture was stirred for 30 min before adding ammonium hydroxide (28%, 12 ml). After 19 h, the solution was diluted with H_2O (15 ml) and washed with Et_2O (15 ml). The H_2O fraction was concentrated under reduced pressure. Purification by size exclusion chromatography and subsequent lyophilization provided the NH_4 salt of title compound **22** (188 mg, 0.304 mmol, 47% over 5 steps, corrected for residual salt) as a white solid. A sample was subsequently purified by HPLC for analytical purposes. EDTA-Na_2 (1 mol%) was added to sharpen the signals. **^1H NMR** (500 MHz, D_2O): δ 8.52 (s, 1H), 8.25 (s, 1H), 6.12 (d, J = 5.6 Hz, 1H), 5.29 (d, J = 2.3 Hz, 1H), 4.75 (t, J = 5.4 Hz, 1H), 4.53 (dd, J = 5.1, 3.8 Hz, 1H), 4.42 – 4.37 (m, 1H), 4.30 – 4.19 (m, 3H), 4.17 – 4.09 (m, 2H), 4.05 –

3.99 (m, 1H), 3.95 (dd, J = 4.7, 2.3 Hz, 1H). **^{13}C NMR** (126 MHz, D_2O): δ 154.2, 150.9, 148.9, 140.5, 118.6, 94.4, 87.3, 84.0, 83.9, 83.9, 82.2, 82.1, 74.5, 74.5, 70.3, 70.2, 65.7, 65.7, 65.2, 65.2. **HRMS** (ESI) m/z: [M + H]$^+$ calculated for $C_{15}H_{22}N_8O_{13}P_2H$ 585.0854; Found 585.0856.

1-α-Azido-2,3-bis-*O*-(4-methoxybenzyl)-ADPr (24).

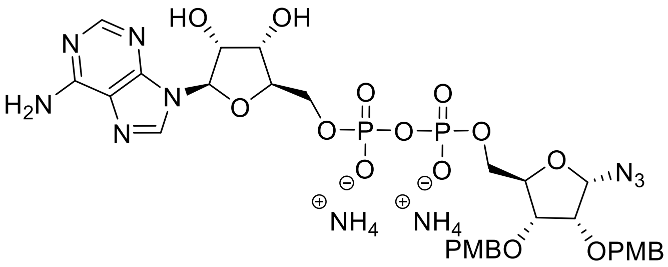

A solution of compound **17** (400 mg, 0.470 mmol) and Et_3N (3.30 ml, 23.5 mmol) in anhydrous MeCN (10 ml) was stirred for 3 days at rt. The solution was concentrated under reduced pressure and the crude residue was co-evaporated with a mixture of anhydrous pyridine (3x). DCI (139 mg, 1.17 mmol) was added and the mixture was co-evaporated with anhydrous MeCN (3x) before adding anhydrous MeCN (12 ml). Compound **20** (401 mg, 0.563 mmol) was co-evaporated with anhydrous toluene (3x) before adding dropwise to the white suspension using anhydrous MeCN (5 ml). The reaction mixture was vigorously stirred under argon atmosphere for 1 h before adding *t*-BuOOH (5.5 M, 0.43 ml, 2.4 mmol). The reaction was monitored by ^{31}P NMR until no residual phosphotriester signals at 125-127 ppm were observed. Upon completion, DBU (0.35 ml, 2.4 mmol) was added and the mixture was stirred for 30 min before adding ammonium hydroxide (28%, 17 ml). After 16 h, the reaction mixture was concentrated under reduced pressure. Purification by preparative HPLC and subsequent lyophilization provided the NH_4 salt of title compound **24** (109 mg, 0.47 mmol, 27 % over 5 steps) as a white solid. **^{1}H NMR** (500 MHz, D_2O): δ 8.38 (s, 1H), 8.04 (s, 1H), 7.12 – 7.06 (dd, J = 10.7, 8.7 Hz 4H), 6.77 (dd, J = 8.7, 3.7 Hz, 4H), 5.95 (d, J = 5.1 Hz, 1H), 5.36 (d, J = 4.9 Hz, 1H), 4.56 (t, J = 5.1 Hz, 1H), 4.46 (t, J = 4.7 Hz, 1H), 4.34 (m, 4H), 4.28 – 4.13 (m, 4H), 4.10 (s, J = 5.4 Hz, 1H), 3.99 (dd, J = 5.9, 3.0 Hz, 1H), 3.95 (t, J = 3.2 Hz, 2H), 3.74 (s, 3H), 3.73 (s, 3H). **^{13}C NMR** (126 MHz, D_2O): δ 179.4, 158.7, 158.6, 154.3, 151.3, 148.4, 139.7, 130.2, 130.1, 129.3, 129.2, 118.4, 113.7, 113.7, 90.3, 87.3, 83.5, 82.9, 77.9, 75.4, 74.6, 72.6, 71.8, 70.1, 65.5, 65.1, 55.3. **^{31}P NMR** (202 MHz, D_2O): δ -10.54. **HRMS** (ESI) m/z: [M + Na]$^+$ calculated for $C_{15}H_{22}N_8O_{13}P_2Na$ 825.2005; Found 825.2003.

PMB deprotection:

To a solution of **24** (30 mg, 0.035 mmol) in HFIP (3.5 ml) at 0 °C was added HCl (0.2 M in HFIP, 0.35 ml) in one go. The resulting deep red solution was stirred for 20 minutes before quenching with Et_3N (0.05 ml). The crude residue was participated between milli Q grade H_2O (5 ml) and Et_2O (10 ml). The water fraction was lyophilized to yield deprotected α-azido-ADPr **25** (28 mg, 0.035mmol, quantified as Et_3NH^+ salt) as a white solid that was used in the click reaction without additional purification.

General Cu(I)-catalyzed click reaction procedure:

The alkyne carrying oligopeptide (1.0 eq) and N_3-ADPr analogue (1.5-2.0 eq) were dissolved in milliQ water with a final concentration of 3 mM and bubbled with argon gas for 10 min. In the meantime, a click mixture was freshly prepared by adding a solution of NaAsc (0.61 M in H_2O, 8 eq) to a solution of $CuSO_4$ (0.16 M in H_2O, 1.0-1.3 eq) directly followed by THPTA (0.1 M in H_2O, 1.0-2.0 eq). After addition of the click mixture, the reaction was stirred at rt and monitored using LC-MS (C18-column, 10-50% MeCN/H_2O + 1% TFA, 12.5 min) until the oligopeptide was fully depleted. Upon completion, the reaction mixture was quenched with

EDTA (0.5 M in H_2O, 2 eq) and concentrated under reduced pressure. The crude residue was purified by a combination of SEC and preparative HPLC and subsequently lyophilized.

Peptide sequences:

PARP1 (529 - 553): H_2N-Gly-Gly-Ala-Ala-Val-Asp-Pro-Asp-Ser-Gly-Leu-Glu-His*-Ser-Ala -COOH (His* = propargylglycine) (26).

Peptide sequence **26** was synthesized according to the general SPPS method as described above. The crude residue was subjected to size exclusion chromatography DOWEX30 followed by HPLC purification. The product fractions were collected and lyophilized to provide title compound **26** (24 mg, 19 µmol, 19%) as a white solid. **LC-MS** R_t = 4.11 min (10-50% MeCN/H_2O, NH_4OAc). **HRMS** (ESI) m/z: $[M + H]^+$ calculated for $C_{61}H_{96}N_{14}O_{17}H$ 1341.5994; Found, 1341.6003.$[M+ 2H]^{2+}$ calculated for $C_{61}H_{96}N_{14}O_{17}H_2$ 670.8019; Found 670.8018.

HPF1 (221 – 233): H_2N-Thr-Phe-His*-Ala-Gly-Leu-Val-Val-Pro-Val-Asp-Lys-COOH (His* = propargylglycine) (27).

Peptide sequence **27** was synthesized according to the general SPPS method as described above. The crude residue was subjected to size exclusion chromatography DOWEX30 followed by HPLC purification. The product fractions were collected and lyophilized to provide title compound **27** (35 mg, 26 µmol, 26%) as a white solid. **LC-MS** R_t = 5.33 min (10-50% MeCN/H_2O, NH_4OAc). **HRMS** (ESI) m/z: $[M + H]^+$ calculated for $C_{61}H_{96}N_{14}O_{17}H$ 1298.7180; Found, 1298.7188. $[M + 2H]^{2+}$ calculated for $C_{61}H_{96}N_{14}O_{17}H_2$ 649.3612; Found 649.3618.

Figure S5. Synthesis of ADP-ribosylated histidine mimetics **24-27** via copper(I)-click chemistry where Pra is propargylglycine.

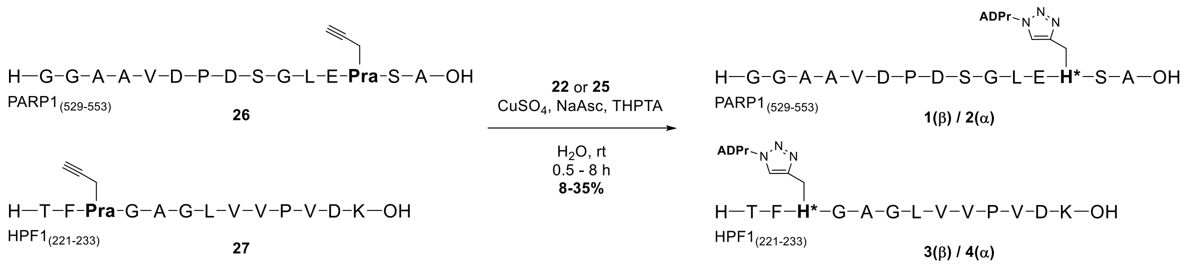

β-PARP1-ADPr: GGAAVDPDSGLE[H*]SA (H* = Triazolyl ADPr) (1).

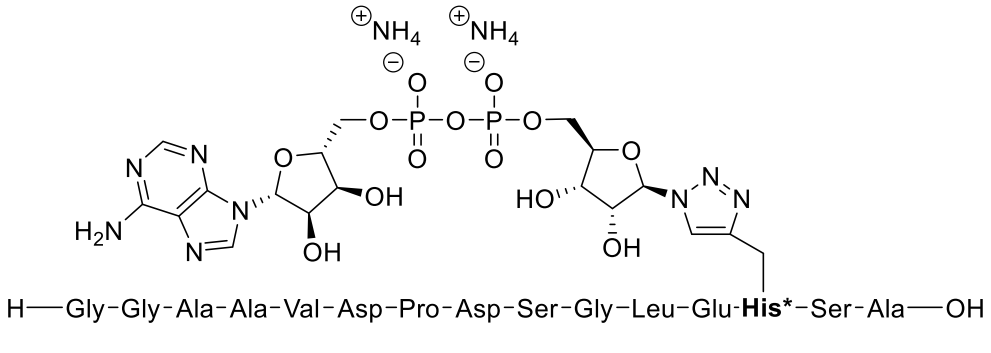

Title compound **1** was synthesized using the general click chemistry procedure. **22** (1.5 mg as NH_4^+ salt, 2.42 µmol, 1.5 eq) was reacted with **26** (2.17 mg, 1.62 µmol, 1.0 eq) for 1.5 h. The molar ratios of the click mixture components were $CuSO_4$:THPTA:NaAsc = 2.0:8.0:1.2. Purification by SEC and preparative HPLC and subsequent lyophilization yielded **1** (0.8 mg, 0.42 µmol, 26%) as a white

solid. **^{1}H NMR** (500 MHz, D_2O): δ 8.44 (s, 1H), 8.20 (s, 1H), 7.94 (s, 1H), 6.07 (d, J = 5.8 Hz, 1H), 5.98 (d, J = 4.5 Hz, 1H). **^{31}P NMR** (202 MHz, D_2O) δ -10.69. **LC-MS** R_t = 2.99 min (10-50% MeCN/H_2O, NH_4OAc). **HRMS** (ESI) m/z: $[M + 2H]^{2+}$ calculated for $C_{70}H_{107}N_{23}O_{37}P_2H_2$ 962.841; Found 962.8405.

α-PARP1-ADPr: GGAAVDPDSGLE[H*]SA (H* = Triazolyl ADPr) (2).

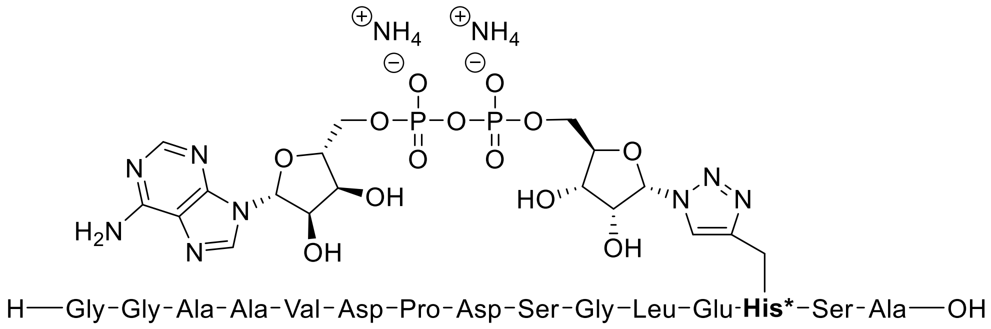

Title compound **2** was synthesized using the general Click chemistry procedure. **25** (4.69 mg as Et_3NH^+ salt, 5.96 µmol, 1.4 eq) was reacted with **26** (5.7 mg, 4.26 µmol, 1.0 eq) for 7 h. The click mixture ratio was $CuSO_4$:THPTA:NaAsc = 1.3:7.2:1.2 Purification by SEC and preparative HPLC and subsequent lyophilization yielded **2** (0.67 mg, 0.35 µmol, 8%) as a white solid. **^{1}H NMR** (500 MHz, D_2O): δ 8.55 (s, 1H), 8.29 (d, *J* = 4.1 Hz, 1H), 7.95 (s, 1H), 6.26 (d, *J* = 5.3 Hz, 1H), 6.12 (d, *J* = 5.6 Hz, 1H). **^{31}P NMR** (202 MHz, D_2O): δ -10.70. **LC-MS** R_t = 3.78 min (10-50% MeCN/H_2O, NH_4OAc). **HRMS** (ESI) m/z: $[M + 2H]^{2+}$ calculated for $C_{76}H_{118}N_{22}O_{30}P_2H_2$ 962.8410; Found 962.8410.

β-HPF1-ADPr: TF[H*]GAGLVVPVDK (H* = Triazolyl ADPr) (3).

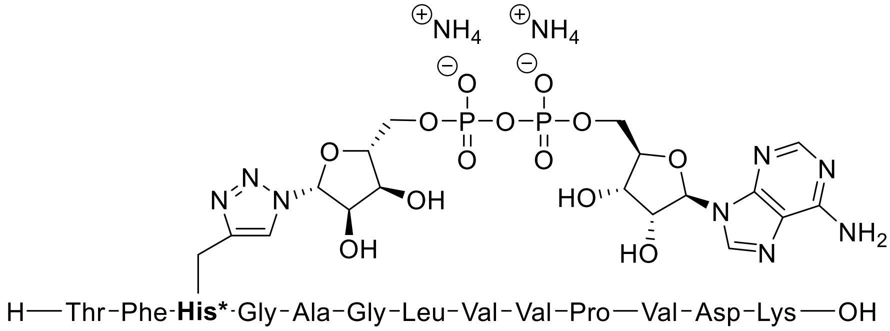

Title compound **3** was synthesized using the general click chemistry procedure. **22** (10.2 mg, as NH_4^+ salt 12.0 µmol, 1.5 eq.) was reacted with **27** (10.0 mg, 7.71 µmol, 1.0 eq.) for 1.5 h. The molar ratio of the click mixture components were $CuSO_4$:THPTA:NaAsc = 1.3:7.8:1.3. Purification by SEC and preparative HPLC and subsequent lyophilization yielded **3** (2.1 mg, 1.11 µmol, 15%) as a white solid. **^{1}H NMR** (500 MHz, D_2O): δ 8.50 (s, 1H), 8.24 (s, 1H), 8.08 (s, 1H), 7.22 (dd, J = 13.7, 7.1 Hz, 3H), 7.08 (d, J = 7.1 Hz, 2H), 6.07 (d, J = 5.7 Hz, 1H), 6.04 (d, J = 4.6 Hz, 1H). **^{31}P NMR** (202 MHz, D_2O): δ -10.67, -10.77. **LC-MS** R_t =5.23 min (10-50% MeCN/H_2O, NH_4OAc). **HRMS** (ESI) m/z: $[M + 2H]^{2+}$ calculated for $C_{76}H_{118}N_{22}O_{30}P_2H_2$ 941.4003; Found 941.4006. $[M + 3H]^{3+}$calculated for $C_{76}H_{118}N_{22}O_{30}P_2H_3$ 627.9359630; Found 627.9363.

α-HPF1-ADPr: TF[H*]GAGLVVPVDK (H* = Triazolyl ADPr) (4).

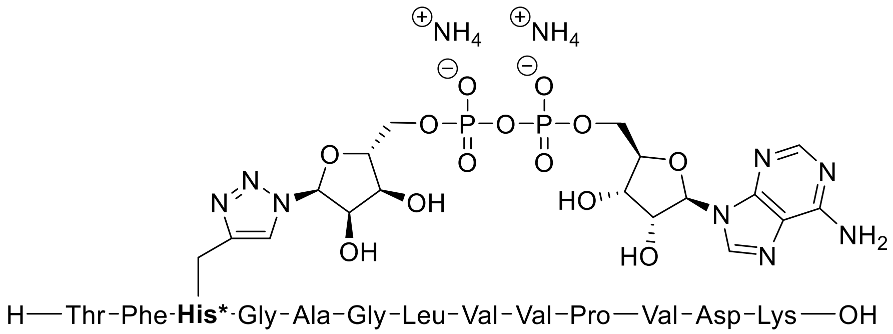

Title compound **4** was synthesized using the general click chemistry procedure. **25** (13.1 mg, as Et_3NH^+ salt 17.0 µmol, 1.4 eq.) was reacted with **27** (15.0 mg, 12.0 µmol, 1.0 eq.) for 3.0 h. The molar ratios of the click mixture components were $CuSO_4$:THPTA:NaAsc = 1.3:8.0:2.0. Purification by preparative HPLC and subsequent lyophlization yielded **4** (7.22 mg, 3.84 µmol, 33%) as a white solid. **^{1}H NMR** (500 MHz, D_2O): δ 8.50 (s, 1H), 8.21 (s, 1H), 7.98 (s, 1H), 7.31 – 7.21 (m, 2H), 7.14 (m, 2H), 6.33 (d, J = 5.3 Hz, 1H), 6.11 (d, J = 5.9 Hz, 1H). **^{31}P NMR** (202 MHz, D_2O): δ -10.39, -10.49, -10.74, -10.85. **LC-MS** R_t = 5.25 min (10-50% MeCN/H_2O, NH_4OAc). **HRMS** (ESI) m/z: $[M + 2H]^{2+}$calculated for $C_{76}H_{118}N_{22}O_{30}P_2H_2$ 941.4003; Found 941.4004. $[M + 3H]^{3+}$ calculated for $C_{76}H_{118}N_{22}O_{30}P_2H_3$ 627.9359; Found 627.9364.

1. Fontana, P. *et al.* Serine ADP-ribosylation reversal by the hydrolase ARH3. *Elife* **6**, 1–20 (2017).
2. Palazzo, L. *et al.* Processing of protein ADP-ribosylation by Nudix hydrolases. *Biochem. J.* **468**, 293–301 (2015).
3. Slade, D. *et al.* The structure and catalytic mechanism of a poly(ADP-ribose) glycohydrolase. *Nature* **477**, 616–620 (2011).
4. Rack, J. G. M. *et al.* (ADP-ribosyl)hydrolases: Structural Basis for Differential Substrate Recognition and Inhibition. *Cell Chem. Biol.* **25**, 1533-1546.e12 (2018).
5. Voorneveld, J. *et al.* Synthetic α- and β-Ser-ADP-ribosylated Peptides Reveal α-Ser-ADPr as the Native Epimer. *Org. Lett.* **20**, 4140–4143 (2018).
6. Voorneveld, J. *et al.* Molecular Tools for the Study of ADP-Ribosylation: A Unified and Versatile Method to Synthesise Native Mono-ADP-Ribosylated Peptides. *Chem. Weinh. Bergstr. Ger.* **27**, 10621–10627 (2021).
7. Štimac, A. & Kobe, J. An improved preparation of 2,3,5-tri-O-acyl-β-d-ribofuranosyl azides by the Lewis acid-catalysed reaction of β-d-ribofuranosyl acetates and trimethylsilyl azide: an example of concomitant formation of the α anomer by trimethylsilyl triflate catalysis. *Carbohydr. Res.* **232**, 359–365 (1992).
8. Zheng, X.-A. *et al.* An efficient and practical synthesis of formylglycinamide ribonucleotide (FGAR). *Nucleosides Nucleotides Nucleic Acids* **37**, 79–88 (2018).
9. Pfaffe, M. & Mahrwald, R. Direct Glycosylation of Unprotected and Unactivated Carbohydrates under Mild Conditions. *Org. Lett.* **14**, 792–795 (2012).
10. Kistemaker, H. A. V. *et al.* Synthesis and Macrodomain Binding of Mono-ADP-Ribosylated Peptides. *Angew. Chem. Int. Ed.* **55**, 10634–10638 (2016).
11. Kicsák, M. *et al.* Tricyclanos: conformationally constrained nucleoside analogues with a new heterotricycle obtained from a D-ribofuranose unit. *Org. Biomol. Chem.* **16**, 393–401 (2018).
12. Kistemaker, H. A. V., Meeuwenoord, N. J., Overkleeft, H. S., Marel, G. A. van der & Filippov, D. V. Solid-Phase Synthesis of Oligo-ADP-Ribose. *Curr. Protoc. Nucleic Acid Chem.* **64**, 4.68.1-4.68.27 (2016).

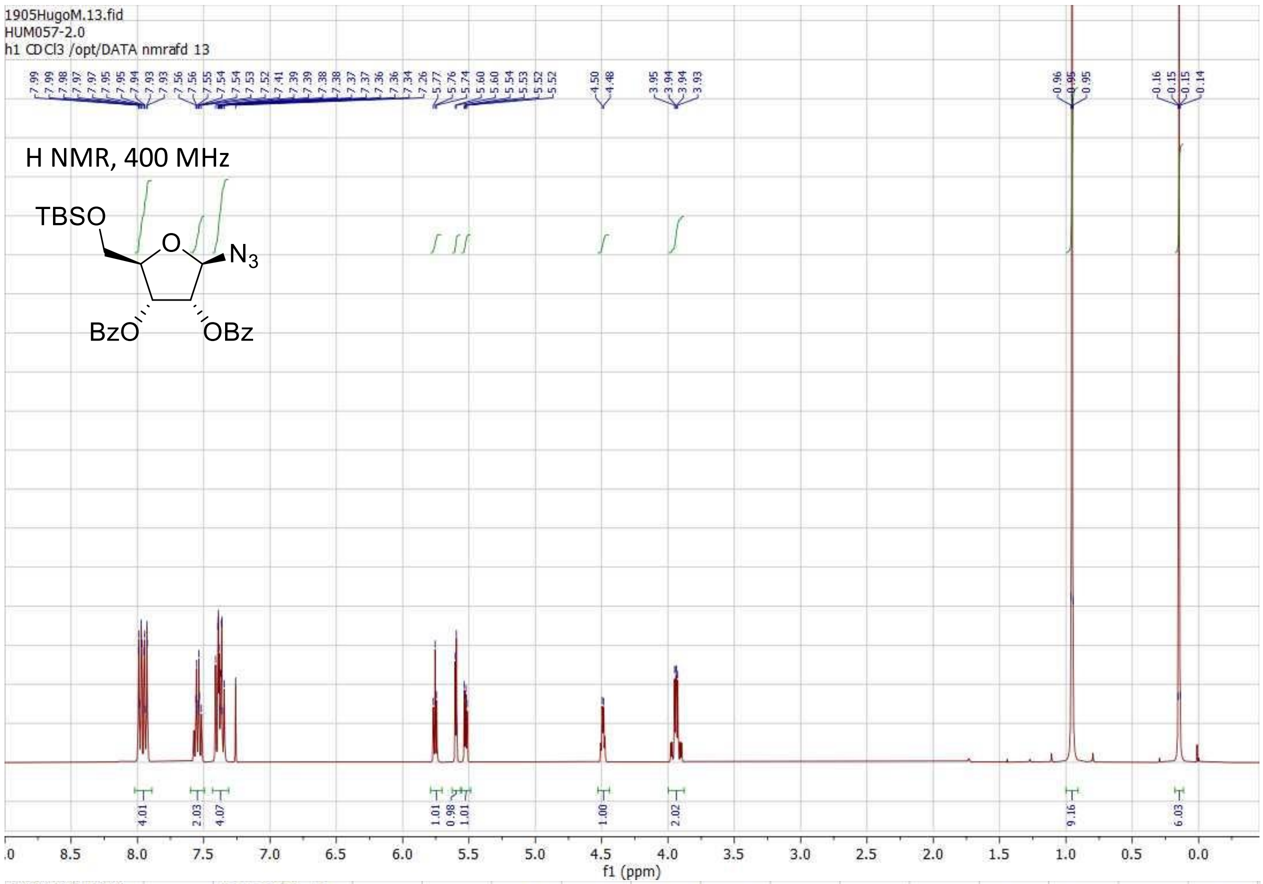
1905HugoM.13.fid
HUM057-2.0
h1 CDCl3 /opt/DATA nmrafd 13
H NMR, 400 MHz
TBSO
O
N3
BzO
OBz
f1 (ppm)

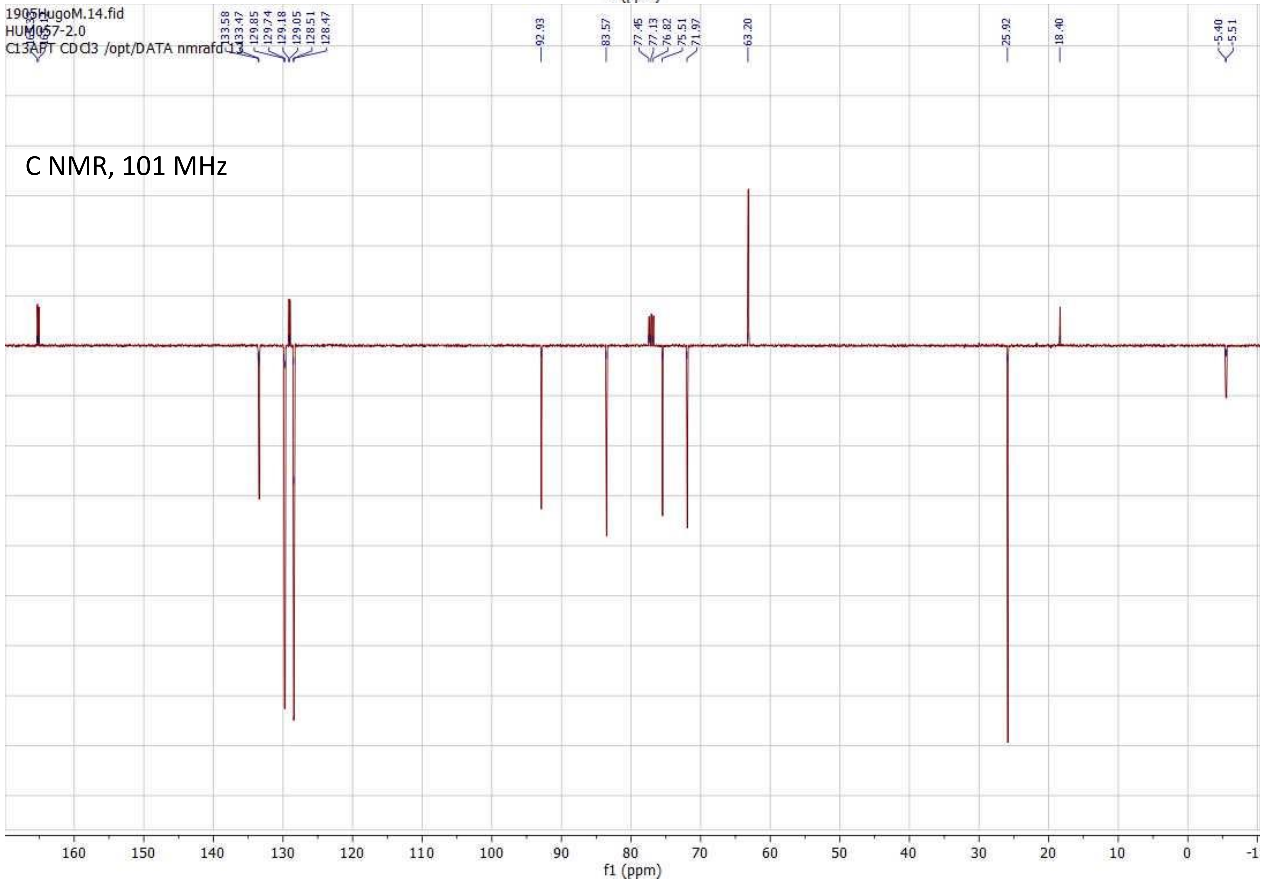
1905HugoM.14.fid
HUM057-2.0
C13APT CDCl3 /opt/DATA nmrafd 13
C NMR, 101 MHz
f1 (ppm)

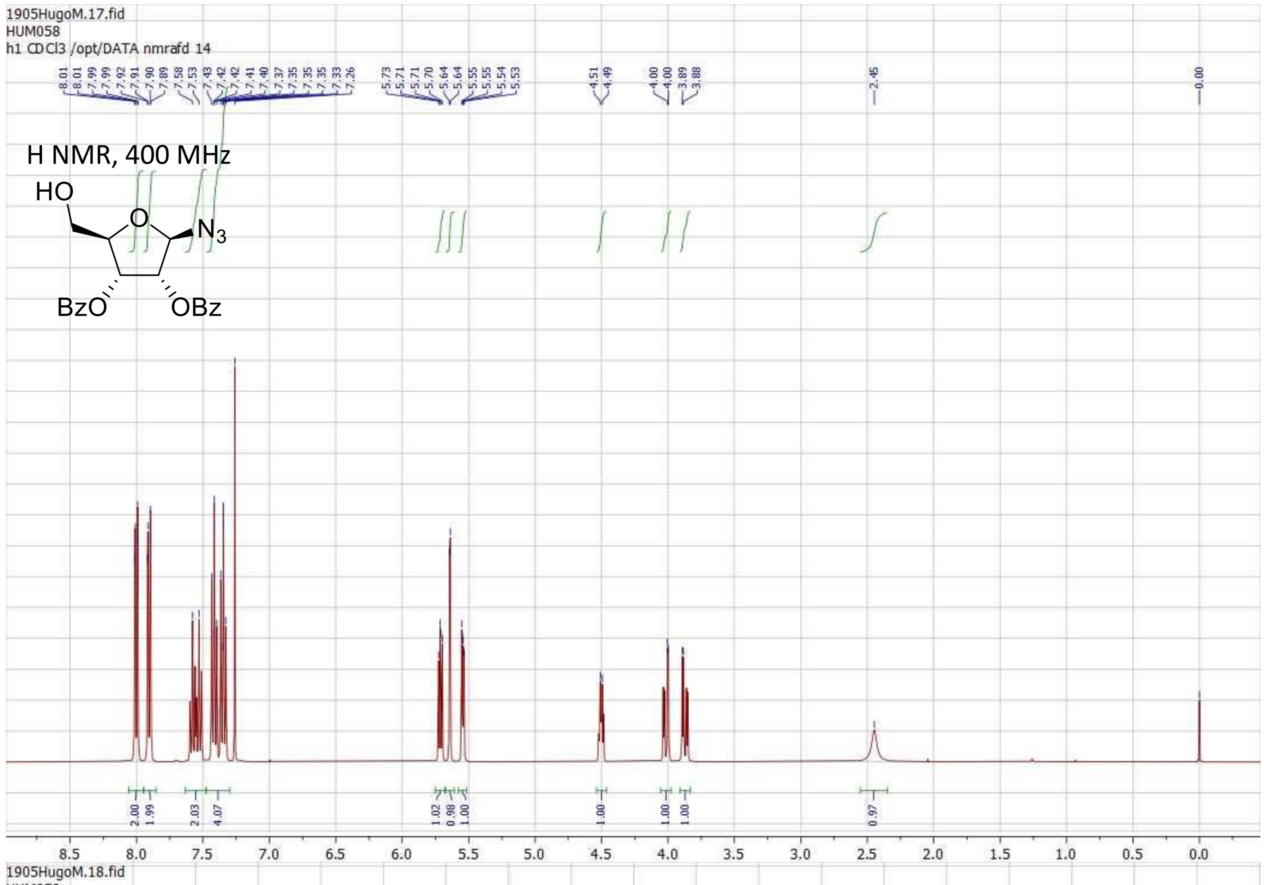
1905HugoM.17.fid
HUM058
h1 CDCl3 /opt/DATA nmrafd 14
H NMR, 400 MHz
HO
O
N3
BzO
OBz

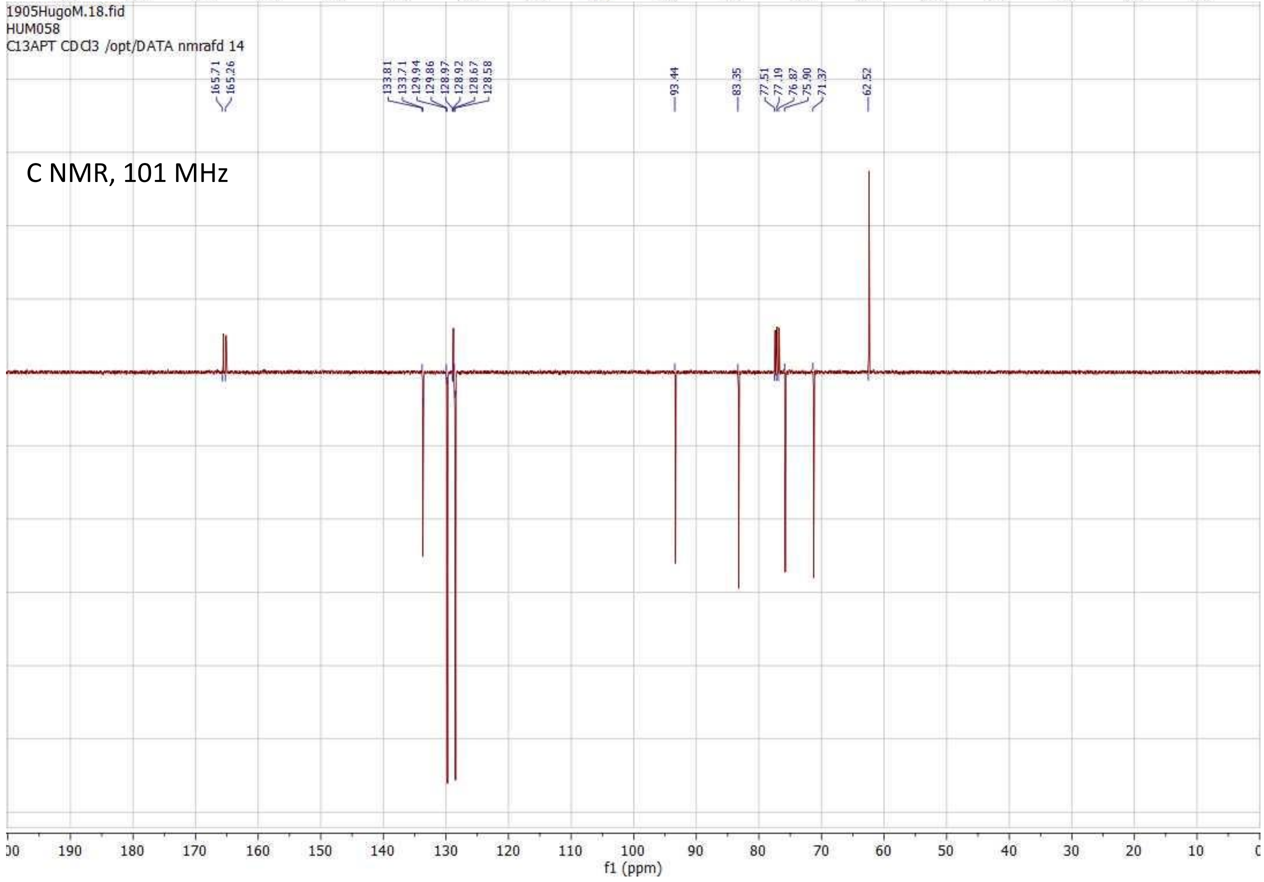
1905HugoM.18.fid
HUM058
C13APT CDCl3 /opt/DATA nmrafd 14
C NMR, 101 MHz
f1 (ppm)

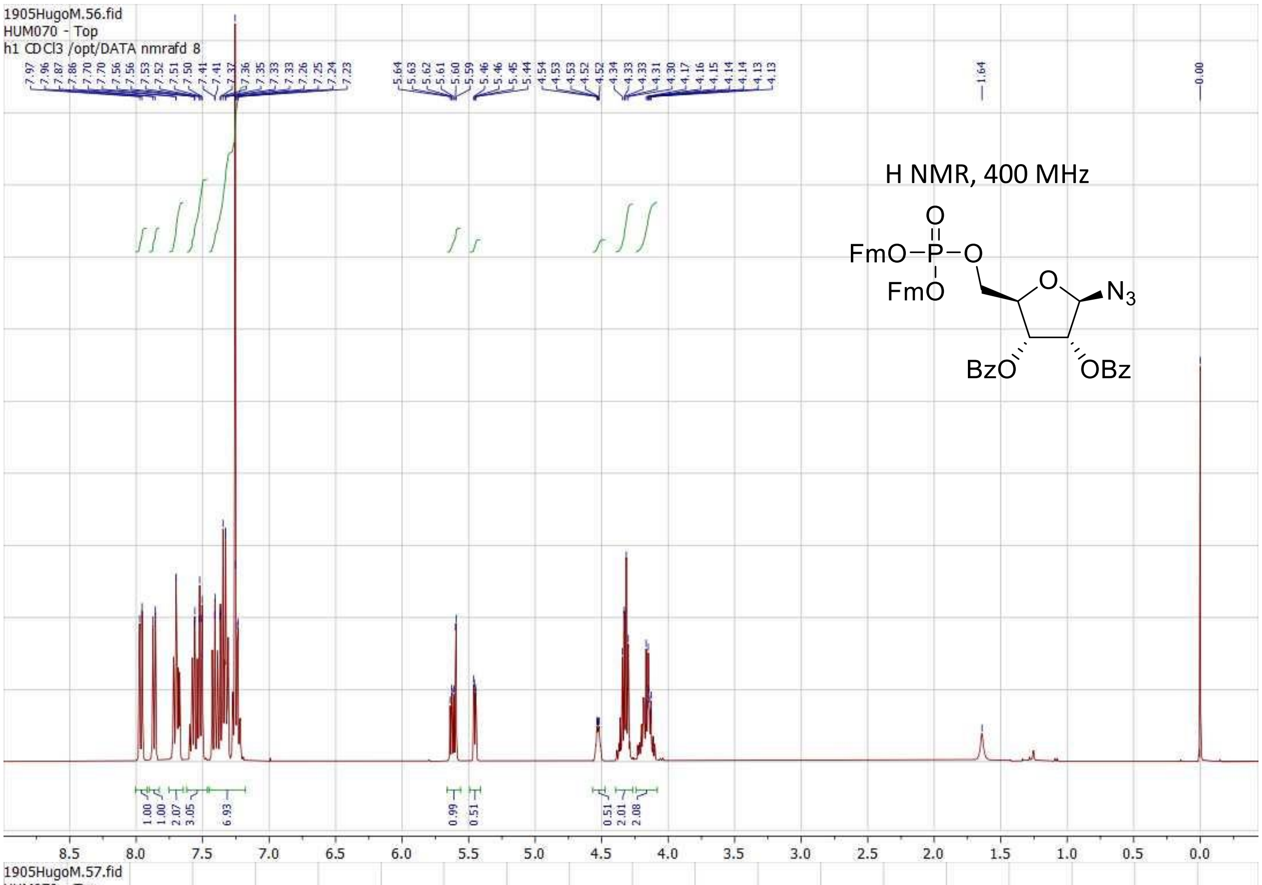
1905HugoM.56.fid
HUM070 - Top
h1 CDCl3 /opt/DATA nmrafd 8
H NMR, 400 MHz
FmO
FmO
BzO
OBz
N3
f1 (ppm)

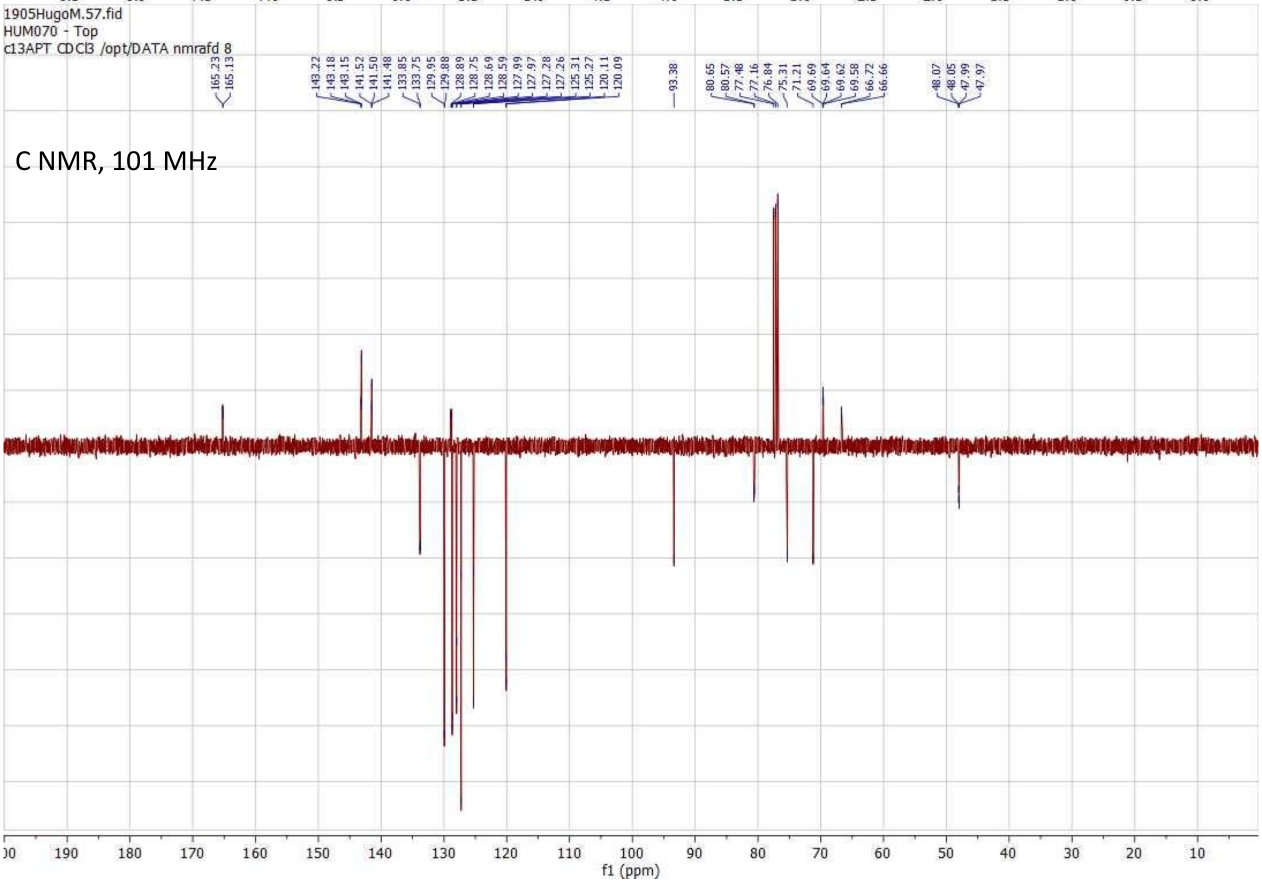
1905HugoM.57.fid
HUM070 - Top
c13APT CDCl3 /opt/DATA nmrafd 8
C NMR, 101 MHz
f1 (ppm)

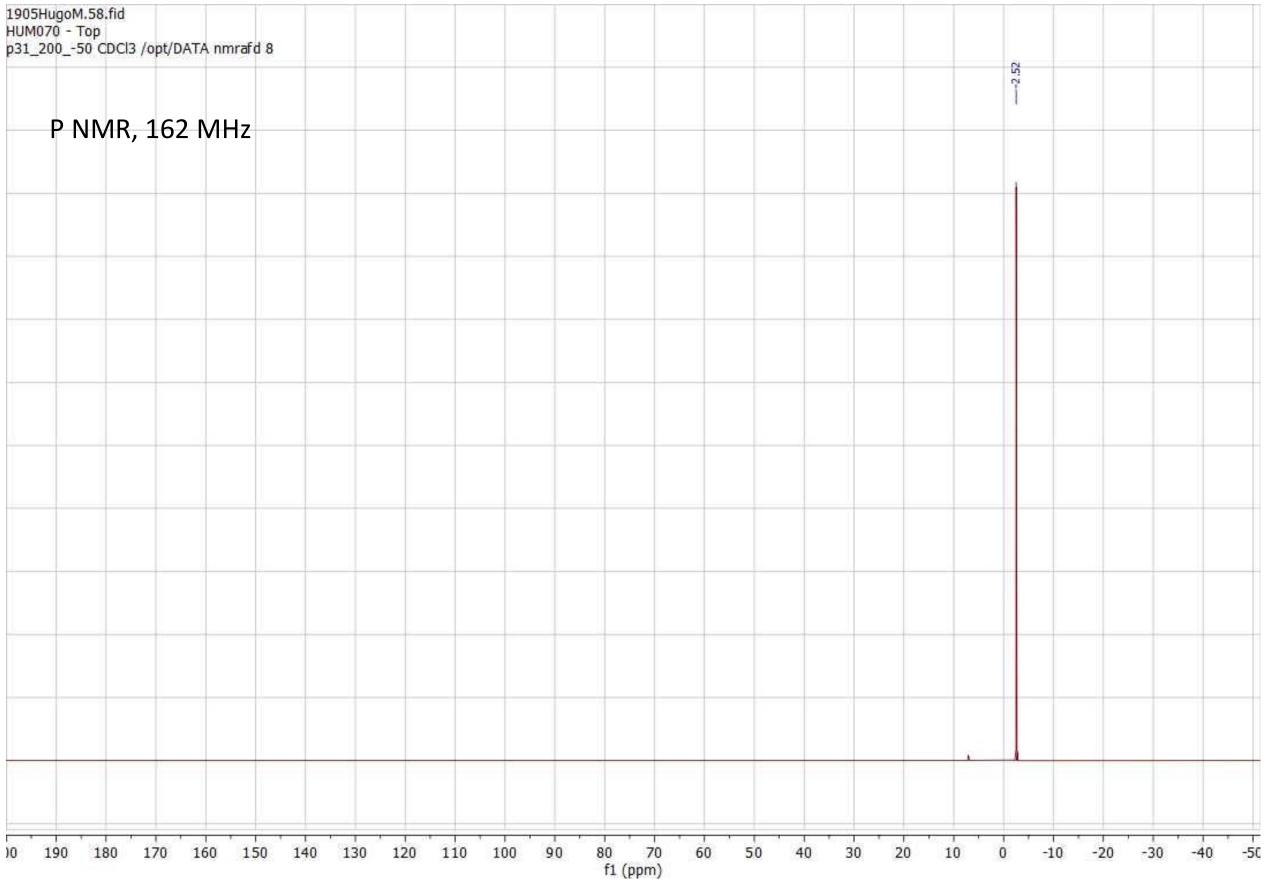
1905HugoM.58.fid
HUM070 - Top
p31_200_-50 CDCl3 /opt/DATA nmrafd 8
P NMR, 162 MHz
-2.52
190 180 170 160 150 140 130 120 110 100 90 80 70 60 50 40 30 20 10 0 -10 -20 -30 -40
f1 (ppm)

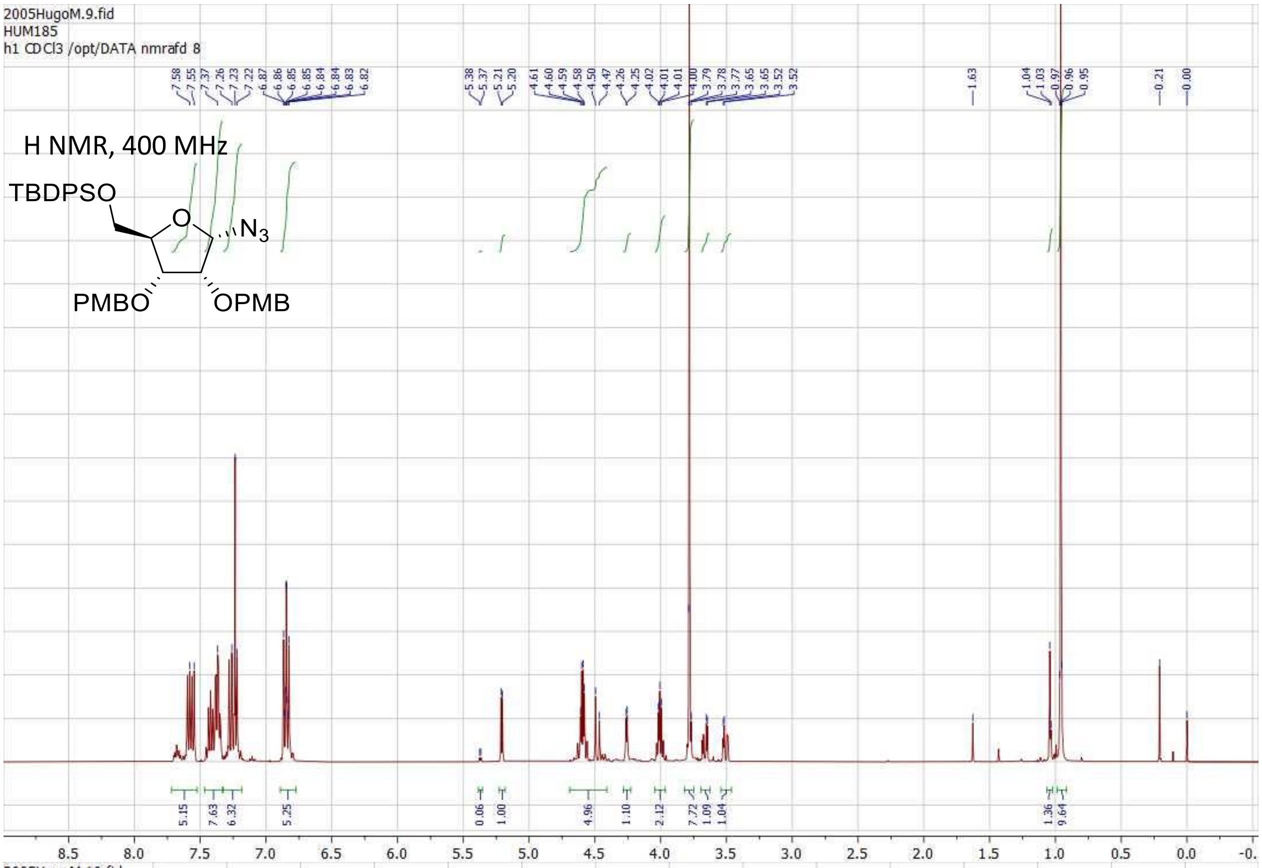
2005HugoM.9.fid
HUM185
h1 CDCl3 /opt/DATA nmrafd 8
H NMR, 400 MHz
TBDPSO
O
N3
PMBO
OPMB
7.58 7.55 7.37 7.26 7.23 7.22 6.87 6.86 6.85 6.85 6.84 6.84 6.83 6.82
5.38 5.37 5.21 5.20
4.61 4.60 4.59 4.58 4.50 4.47 4.26 4.25 4.02 4.01 4.01 4.00 3.79 3.78 3.77 3.65 3.52
1.63
1.04 1.03 0.97 0.96 0.95
0.21
0.00
5.15 7.63 6.32 5.25 0.06 1.00 4.96 1.10 2.12 7.72 1.09 1.04 1.36 9.64
8.5 8.0 7.5 7.0 6.5 6.0 5.5 5.0 4.5 4.0 3.5 3.0 2.5 2.0 1.5 1.0 0.5 0.0

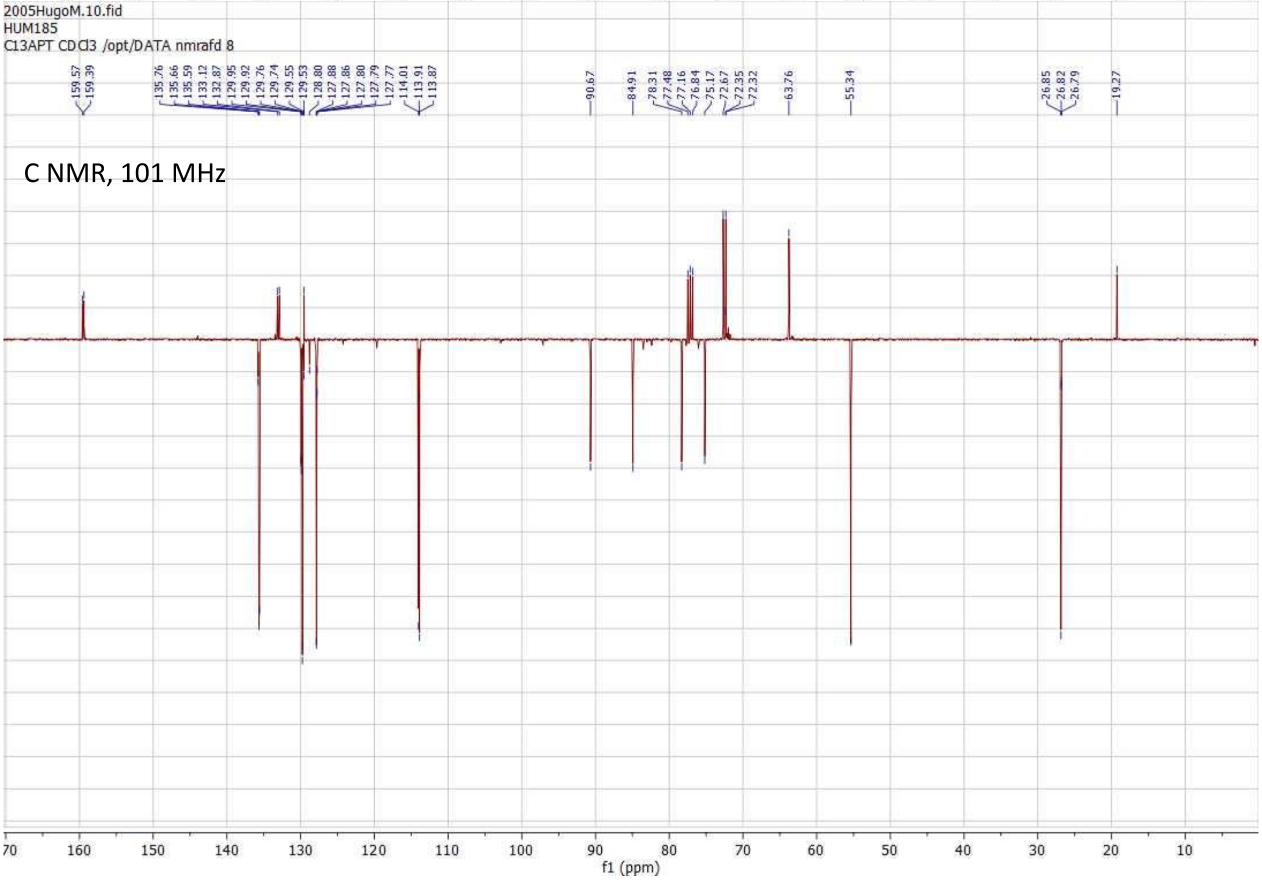
2005HugoM.10.fid
HUM185
C13APT CDCl3 /opt/DATA nmrafd 8
C NMR, 101 MHz
159.57 159.39
135.76 135.66 135.59 133.12 132.87 129.95 129.92 129.76 129.74 129.55 129.53 128.80 127.88 127.86 127.80 127.79 127.77 114.01 113.91 113.87
90.67
84.91
78.31 77.48 77.16 76.84 75.17 72.67 72.35 72.32
63.76
55.34
26.85 26.82 26.79
19.27
70 160 150 140 130 120 110 100 90 80 70 60 50 40 30 20 10
f1 (ppm)

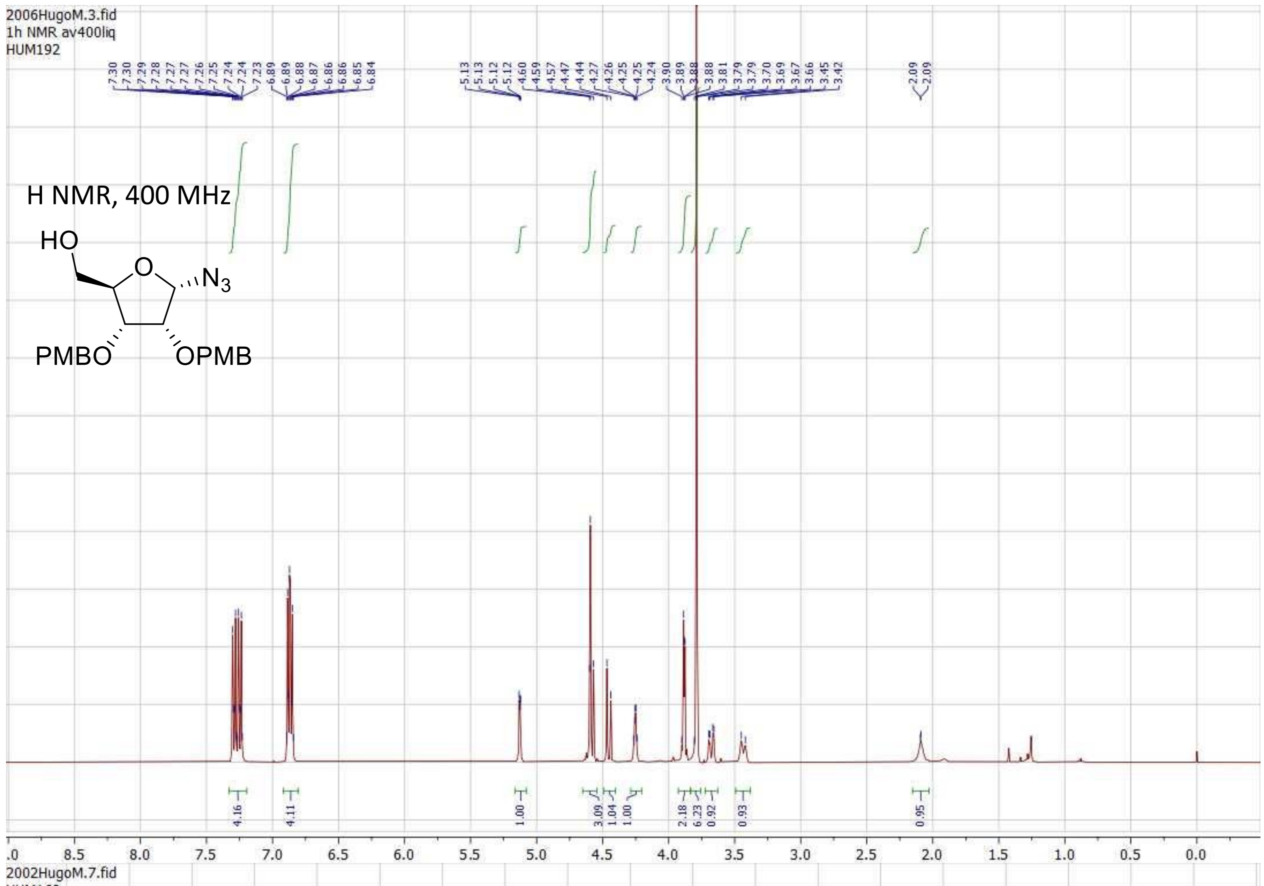
2006HugoM.3.fid
1h NMR av400liq
HUM192
7.30 7.30 7.29 7.28 7.27 7.27 7.26 7.25 7.24 7.24 7.23 6.89 6.89 6.88 6.87 6.86 6.86 6.85 6.84
5.13 5.13 5.12 5.12 4.60 4.59 4.57 4.47 4.44 4.27 4.26 4.25 4.25 4.24 3.90 3.89 3.88 3.88 3.81 3.79 3.79 3.70 3.69 3.67 3.66 3.45 3.42
2.09 2.09
H NMR, 400 MHz
HO
O
N3
PMBO
OPMB
4.16
4.11
1.00
3.09
1.04
1.00
2.18
6.23
0.92
0.93
0.95

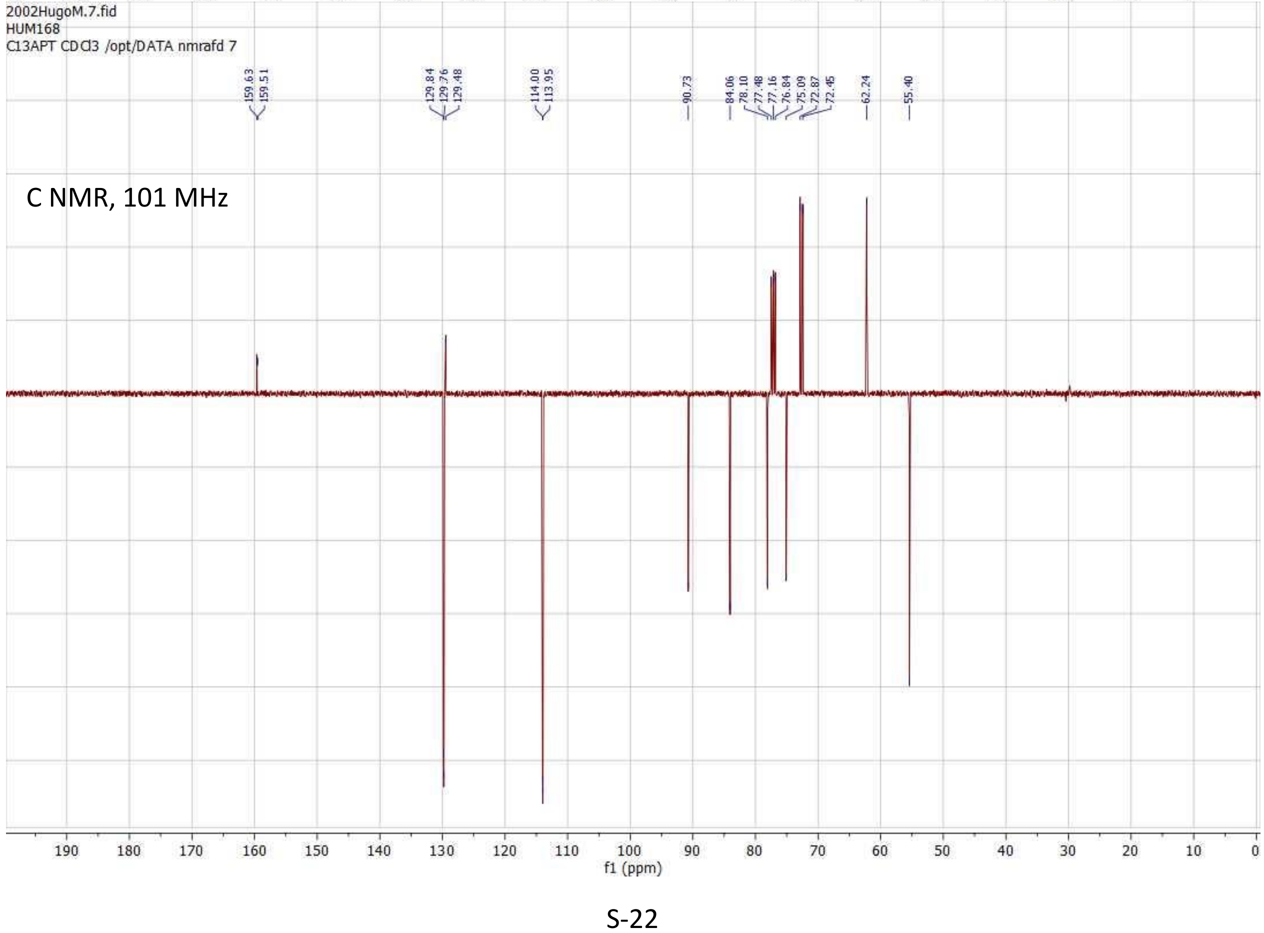
2002HugoM.7.fid
HUM168
C13APT CDCl3 /opt/DATA nmrafd 7
159.63 159.51
129.84 129.76 129.48
114.00 113.95
90.73
84.06 78.10 77.48 77.16 76.84 75.09 72.87 72.45
62.24
55.40
C NMR, 101 MHz
f1 (ppm)

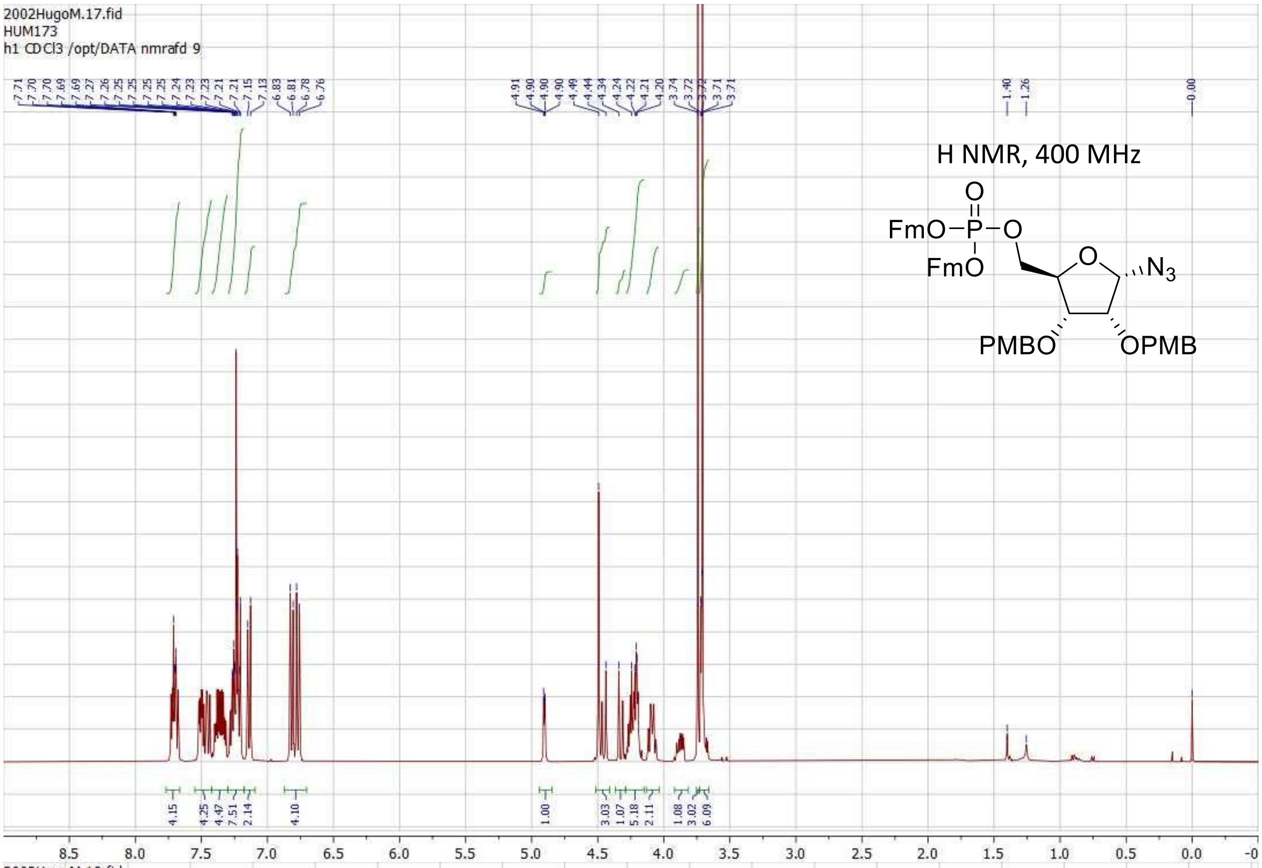
2002HugoM.17.fid
HUM173
h1 CDCl3 /opt/DATA nmrafd 9
H NMR, 400 MHz
FmO
FmO
PMBO
OPMB
N3

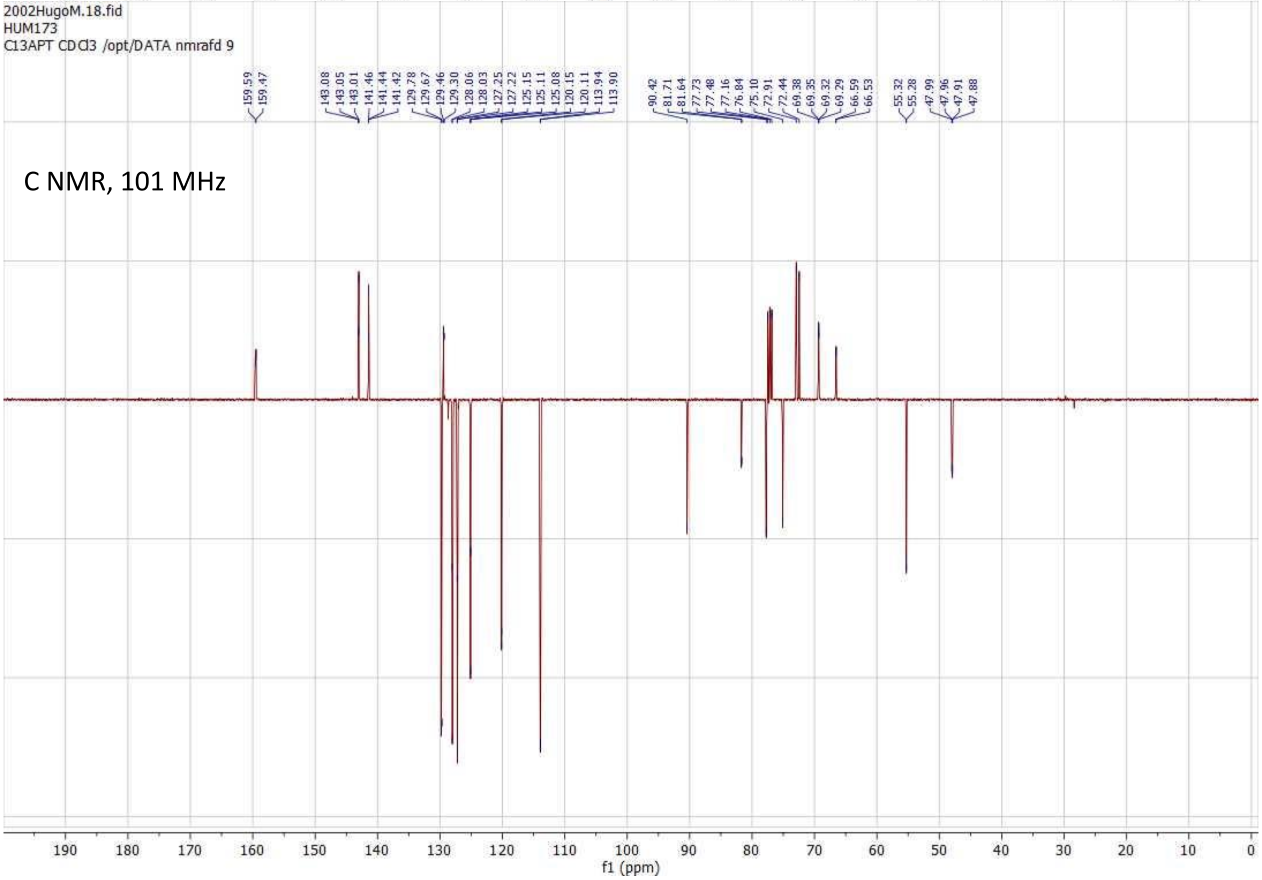
2002HugoM.18.fid
HUM173
C13APT CDCl3 /opt/DATA nmrafd 9
C NMR, 101 MHz
f1 (ppm)

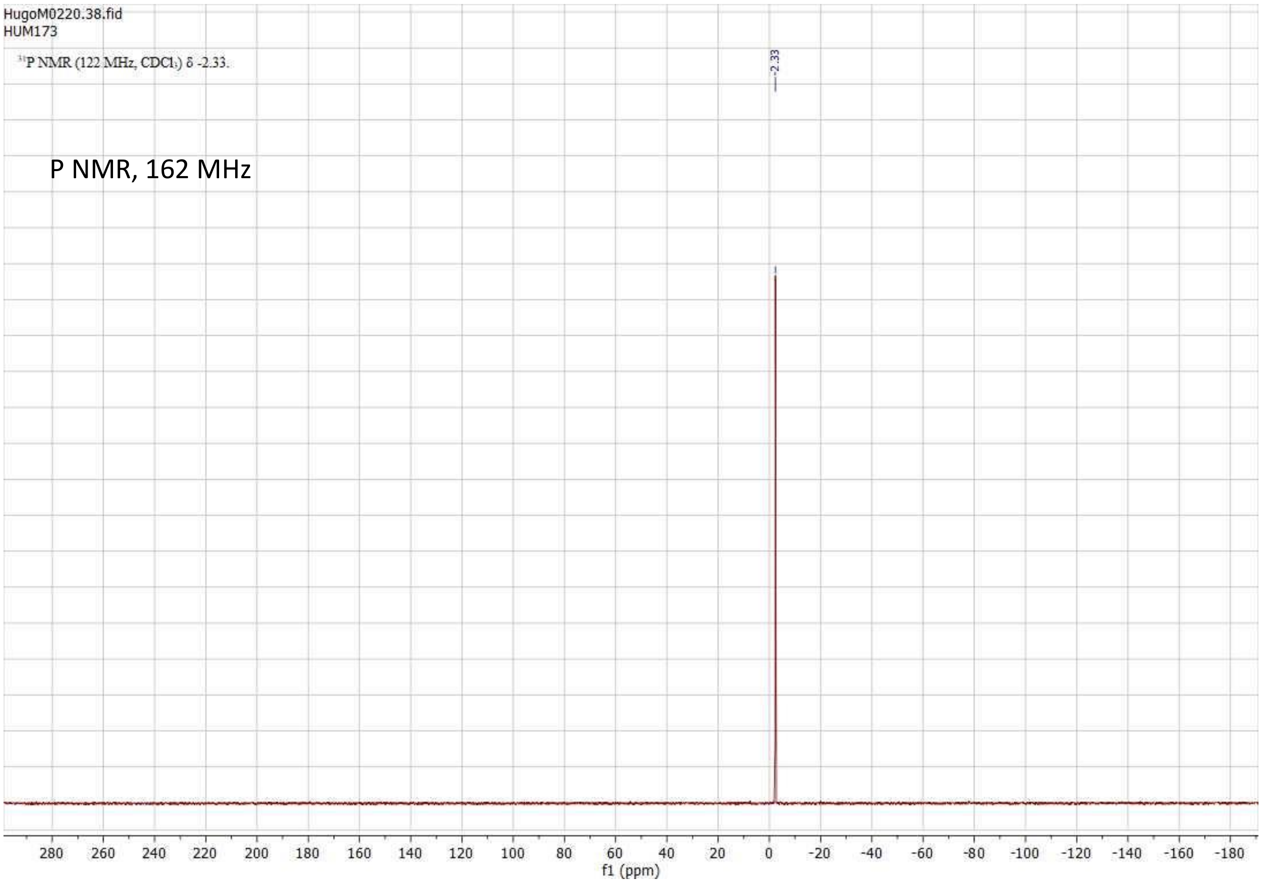

HugoM0220.38.fid
HUM173
31P NMR (122 MHz, CDCl3) δ -2.33.
-2.33
P NMR, 162 MHz
280 260 240 220 200 180 160 140 120 100 80 60 40 20 0 -20 -40 -60 -80 -100 -120 -140 -160 -180
f1 (ppm)

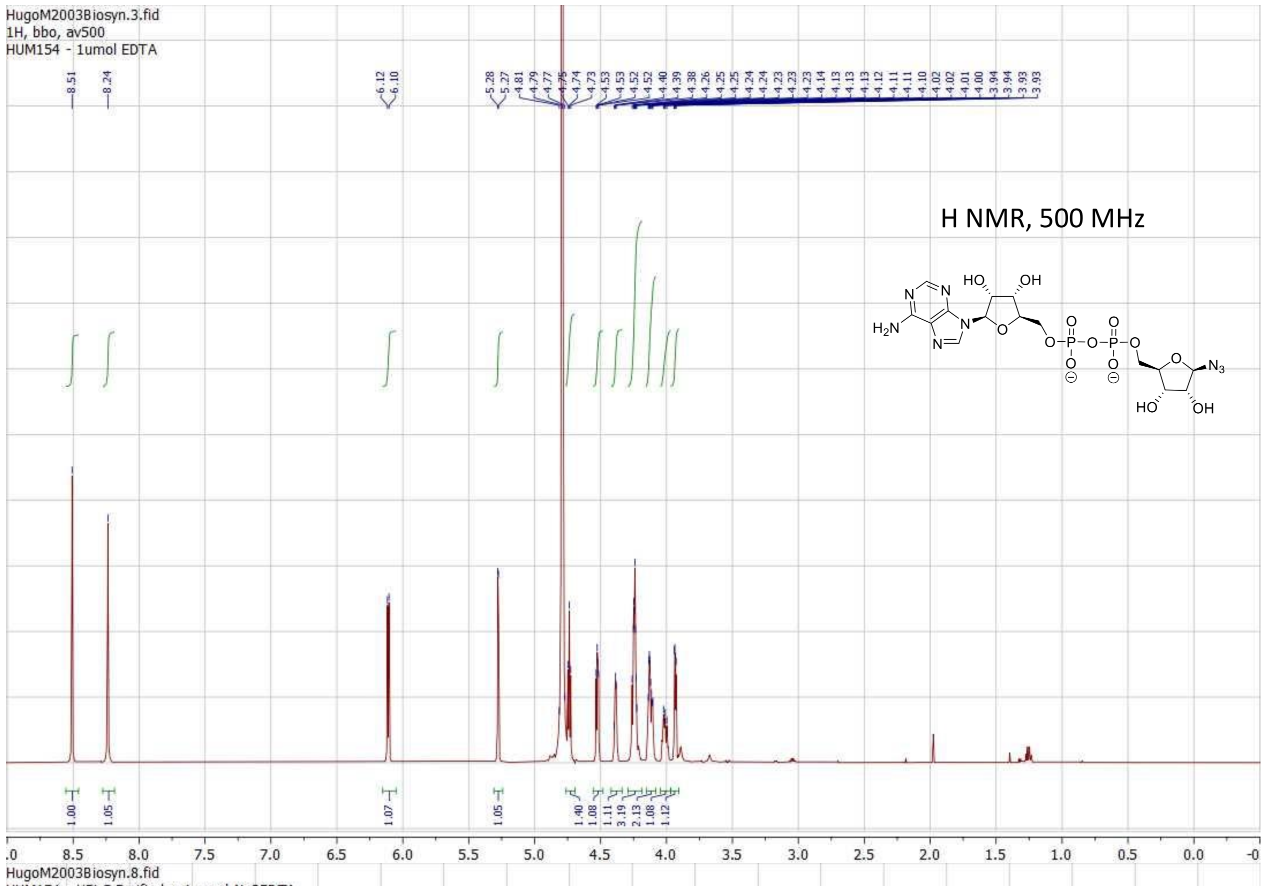
HugoM2003Biosyn.3.fid
1H, bbo, av500
HUM154 - 1umol EDTA
8.51
8.24
6.12
6.10
5.28
5.27
H NMR, 500 MHz
HO
OH
N
H2N
O
N3
HO
OH
1.00
1.05
1.07
1.05
1.40
1.08
1.11
3.19
2.13
1.08
1.12

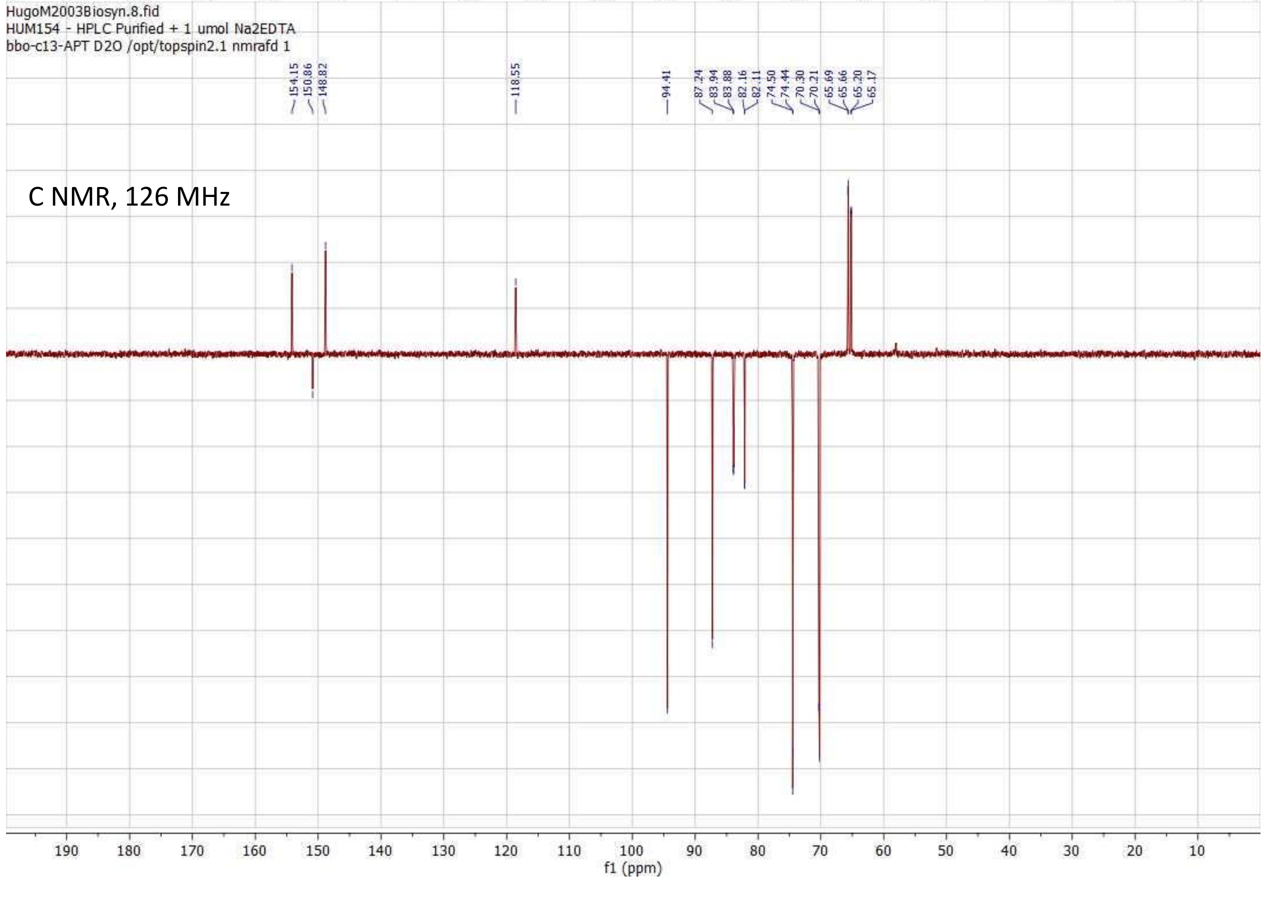
HugoM2003Biosyn.8.fid
HUM154 - HPLC Purified + 1 umol Na2EDTA
bbo-c13-APT D2O /opt/topspin2.1 nmrafd 1
154.15
150.86
148.82
118.55
94.41
87.24
83.94
83.88
82.16
82.11
74.50
74.44
70.30
70.21
65.69
65.66
65.20
65.17
C NMR, 126 MHz
f1 (ppm)

HugoM2003Biosyn.9.fid
HUM154 - HPLC Purified + 1 umol Na2EDTA
bbo-p31-200_-50ppm D2O /opt/topspin2.1 nmrafd 1

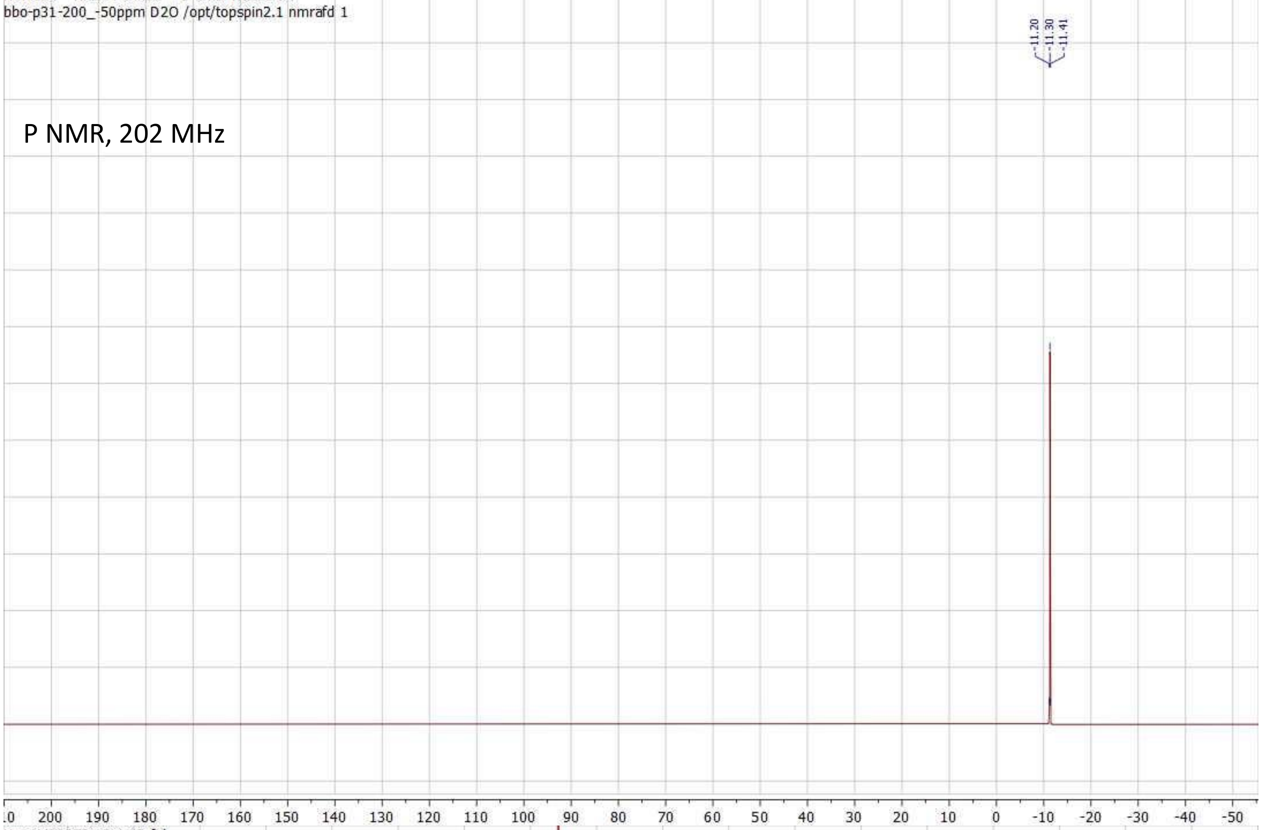

HugoM2007BioSyn.22.fid
HUM181 - HPLC + Freeze Dryer
bbo-h1 D2O /opt/topspin2.1 nmrafd 1

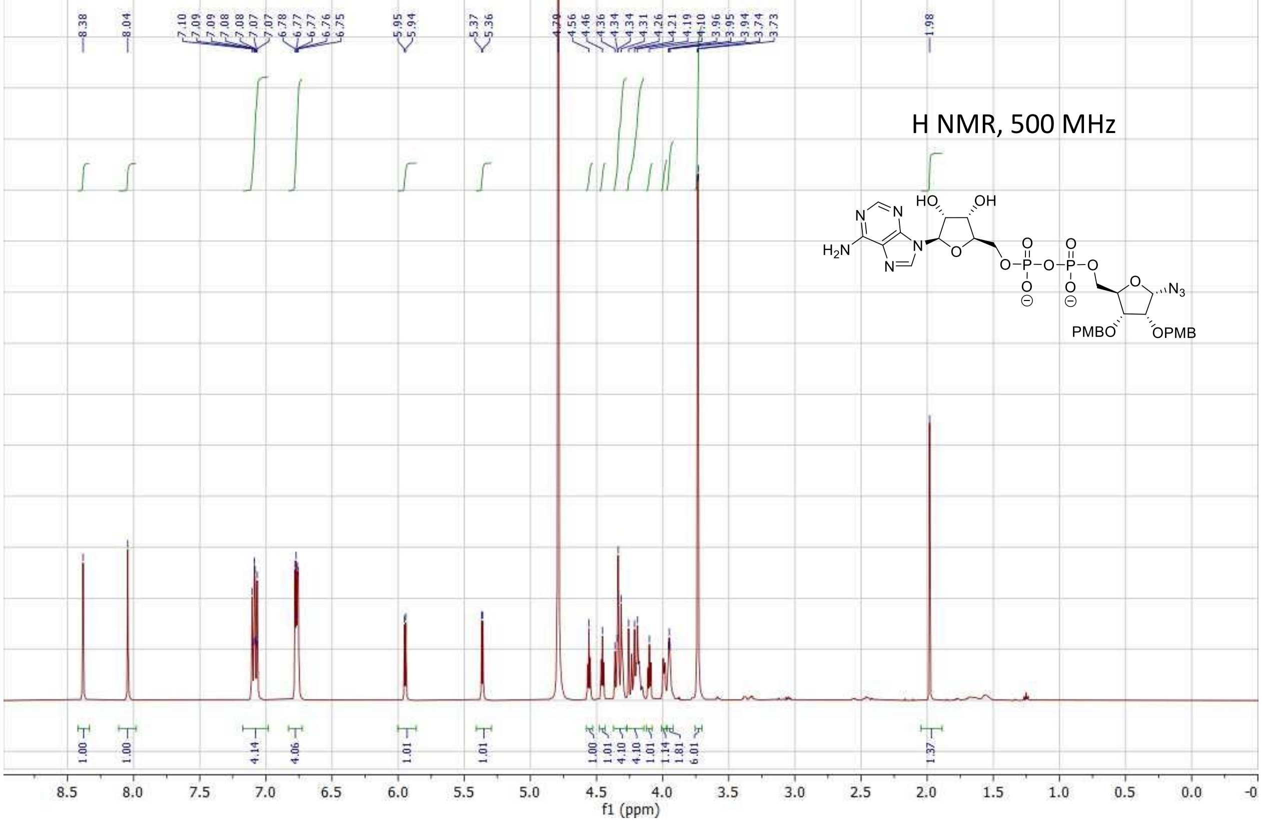

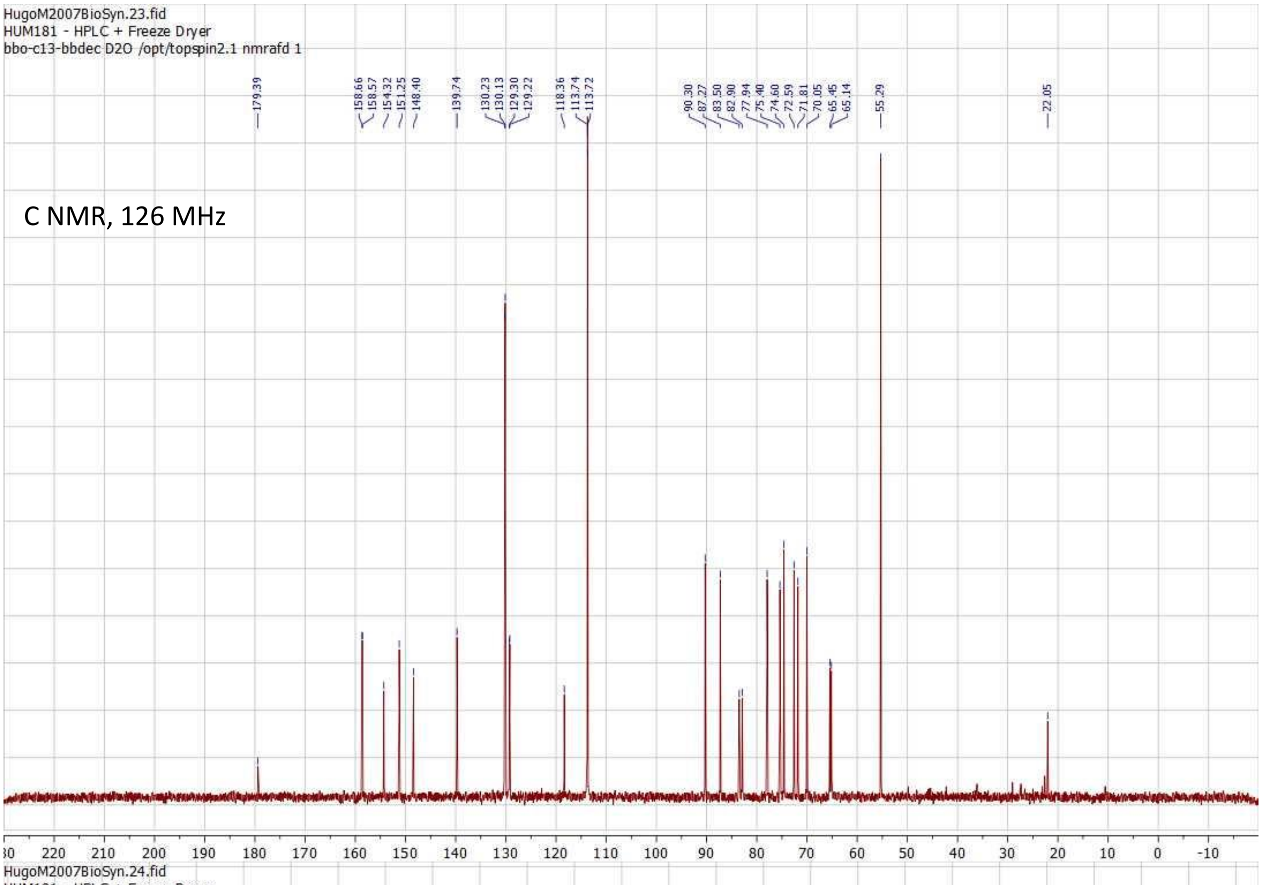
HugoM2007BioSyn.23.fid
HUM181 - HPLC + Freeze Dryer
bbo-c13-bbdec D2O /opt/topspin2.1 nmrafd 1
C NMR, 126 MHz
179.39
158.66
158.57
154.32
151.25
148.40
139.74
130.23
130.13
129.30
129.22
118.36
113.74
113.72
90.30
87.27
83.50
82.90
77.94
75.40
74.60
72.59
71.81
70.05
65.45
65.14
55.29
22.05
220 210 200 190 180 170 160 150 140 130 120 110 100 90 80 70 60 50 40 30 20 10 0 -10

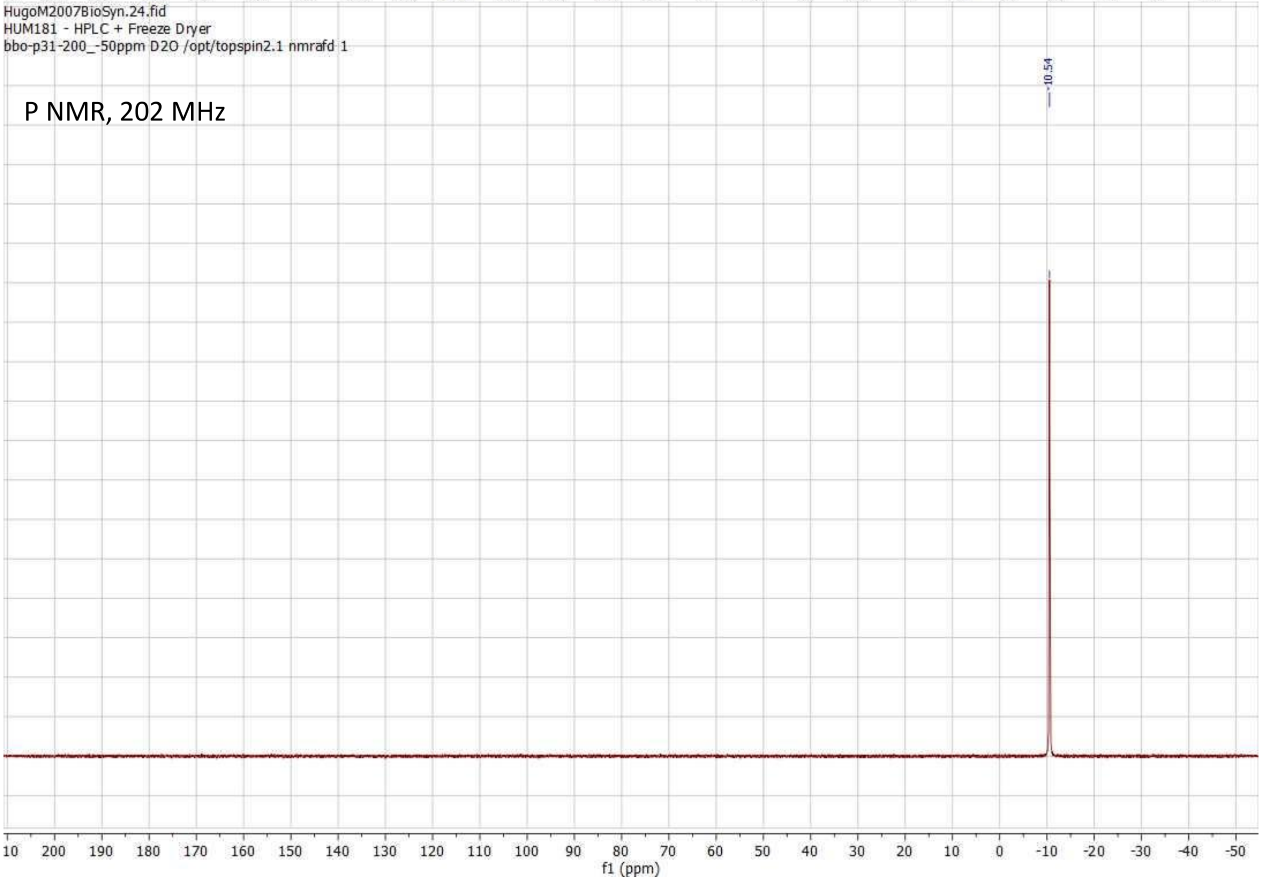
HugoM2007BioSyn.24.fid
HUM181 - HPLC + Freeze Dryer
bbo-p31-200_-50ppm D2O /opt/topspin2.1 nmrafd 1
P NMR, 202 MHz
-10.54
200 190 180 170 160 150 140 130 120 110 100 90 80 70 60 50 40 30 20 10 0 -10 -20 -30 -40 -50
f1 (ppm)

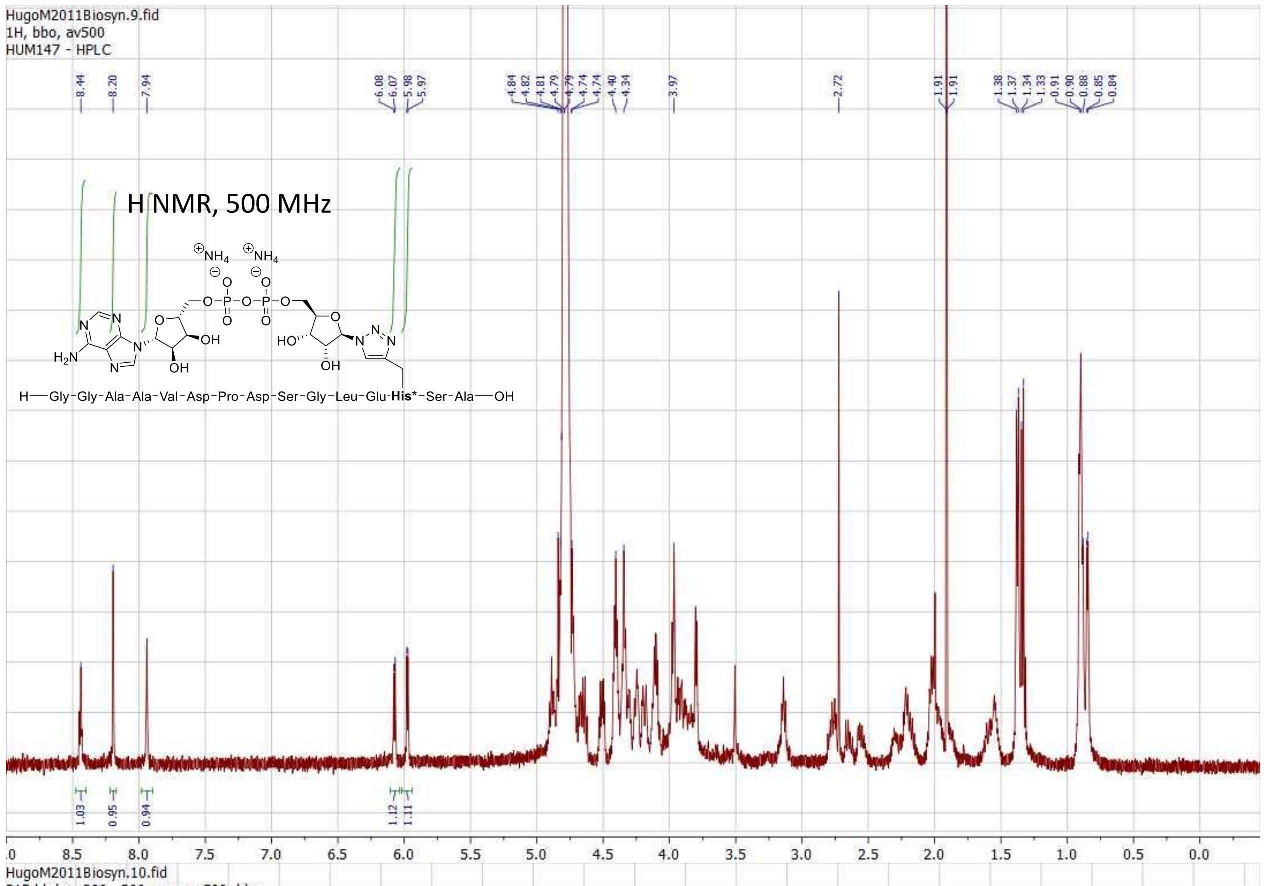
HugoM2011Biosyn.9.fid
1H, bbo, av500
HUM147 - HPLC
H NMR, 500 MHz
H—Gly-Gly-Ala-Ala-Val-Asp-Pro-Asp-Ser-Gly-Leu-Glu-His*-Ser-Ala—OH

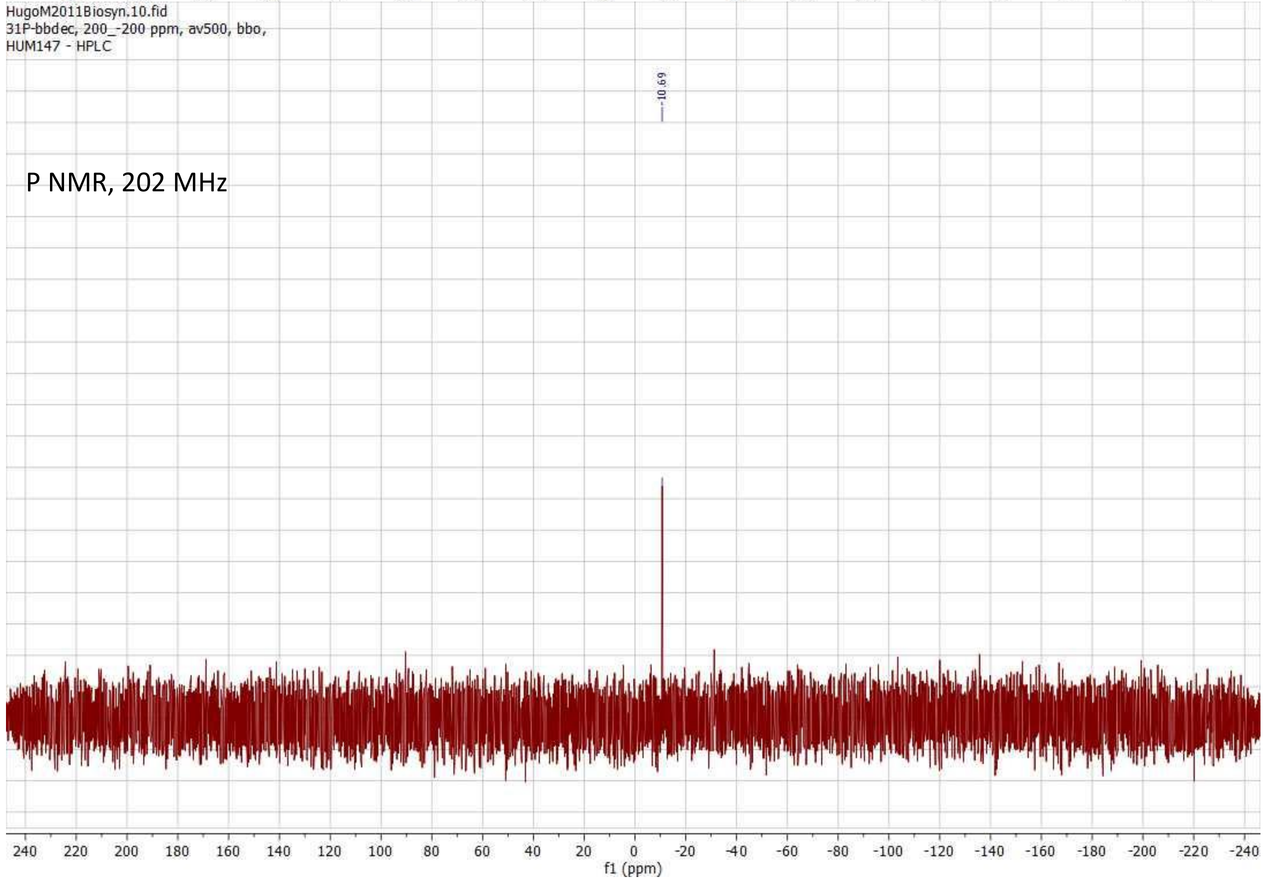
HugoM2011Biosyn.10.fid
31P-bbdec, 200_-200 ppm, av500, bbo,
HUM147 - HPLC
-10.69
P NMR, 202 MHz
f1 (ppm)

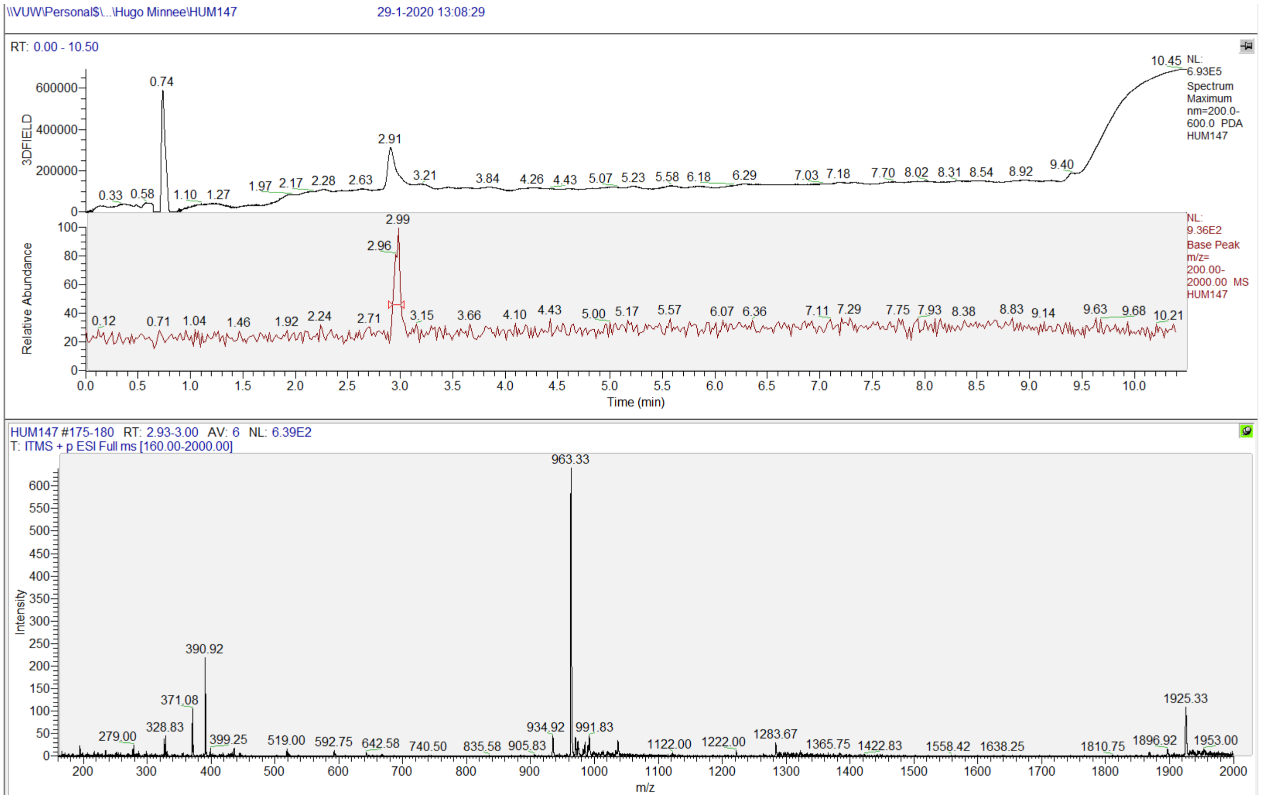

\\VUW\Personal$\...\Hugo Minnee\HUM147
29-1-2020 13:08:29
RT: 0.00 - 10.50
NL: 6.93E5 Spectrum Maximum nm=200.0-600.0 PDA HUM147
NL: 9.36E2 Base Peak m/z= 200.00-2000.00 MS HUM147
3DFIELD
Relative Abundance
Time (min)
HUM147 #175-180 RT: 2.93-3.00 AV: 6 NL: 6.39E2
T: ITMS + p ESI Full ms [160.00-2000.00]
963.33
390.92
371.08
1925.33
Intensity
m/z

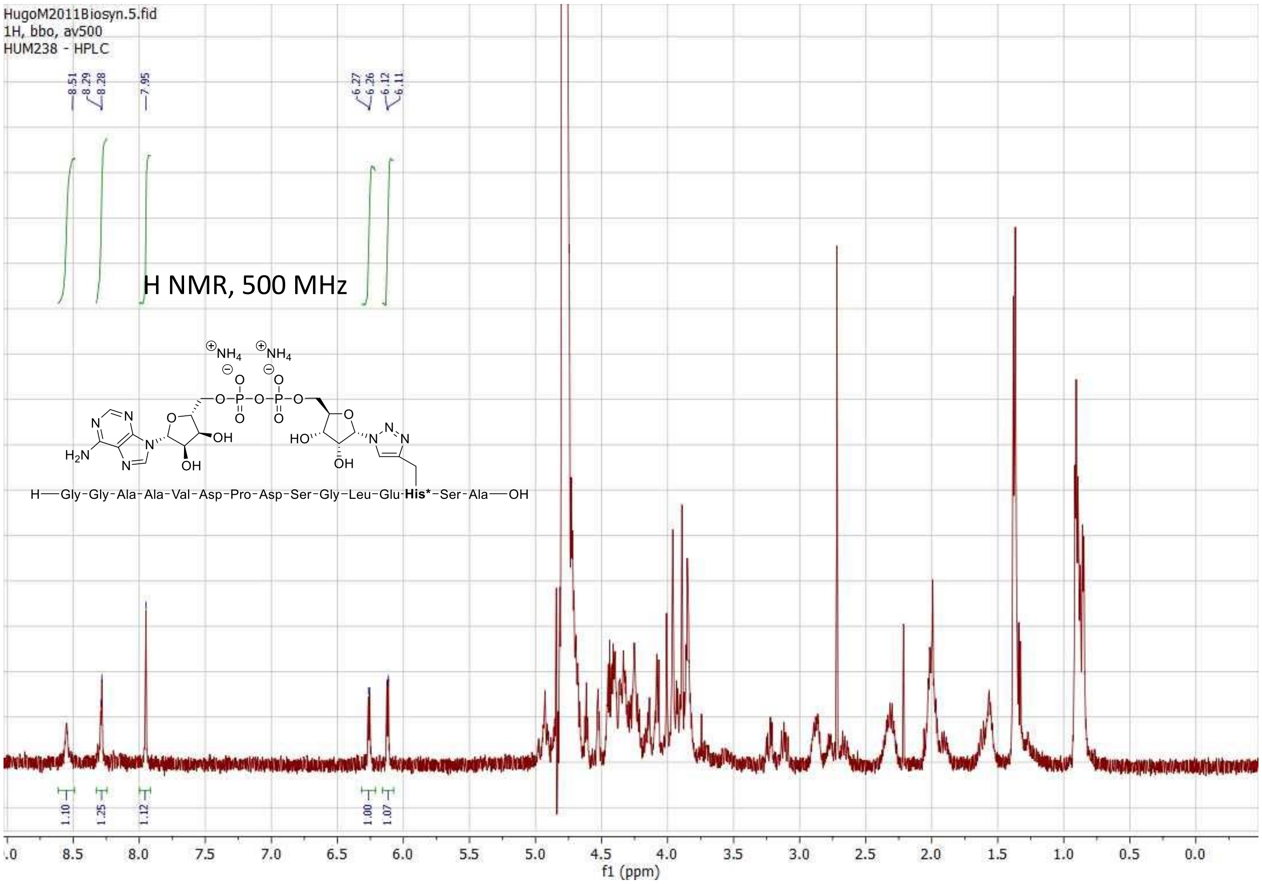

HugoM2011Biosyn.5.fid
1H, bbo, av500
HUM238 - HPLC
H NMR, 500 MHz
H—Gly-Gly-Ala-Ala-Val-Asp-Pro-Asp-Ser-Gly-Leu-Glu-His*-Ser-Ala—OH
f1 (ppm)

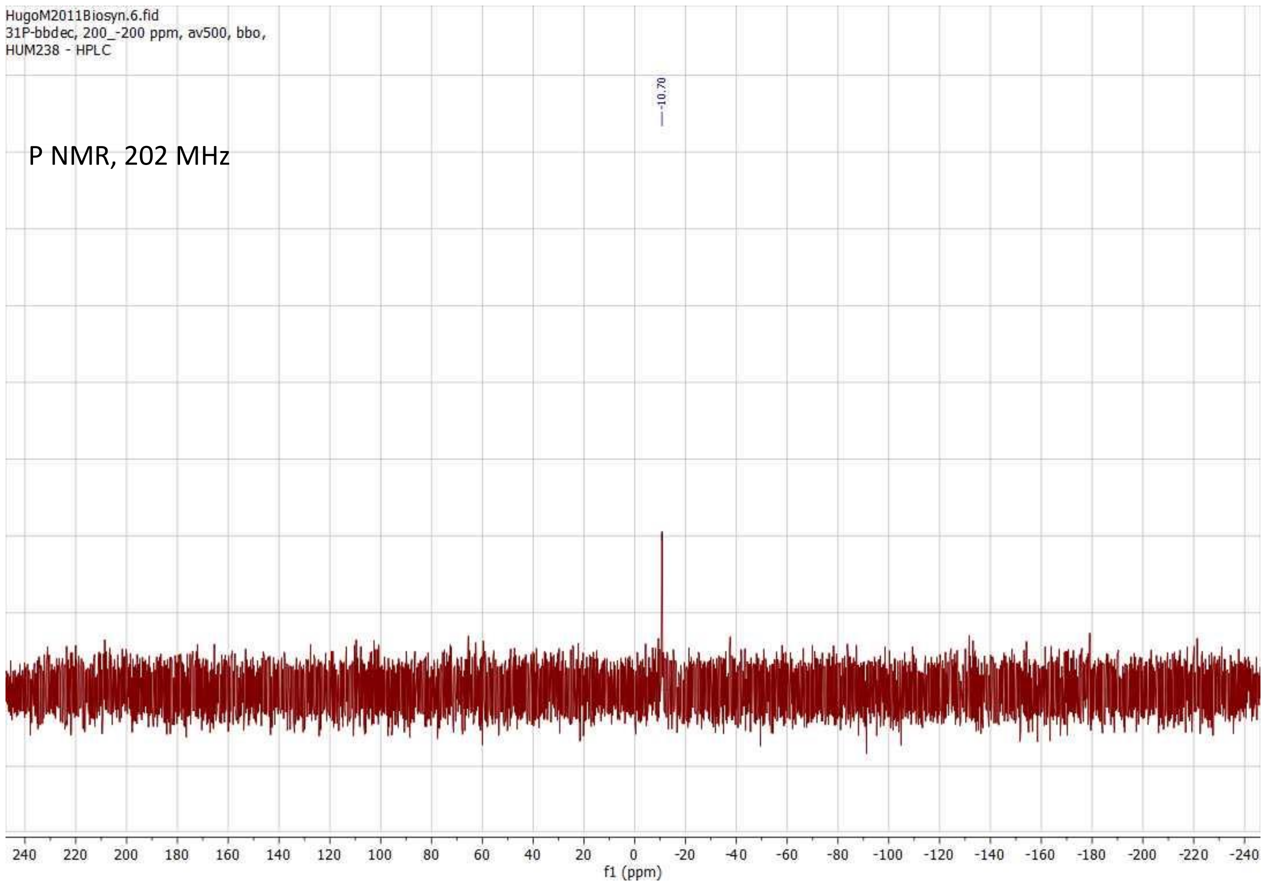
HugoM2011Biosyn.6.fid
31P-bbdec, 200_-200 ppm, av500, bbo,
HUM238 - HPLC
P NMR, 202 MHz
-10.70
f1 (ppm)

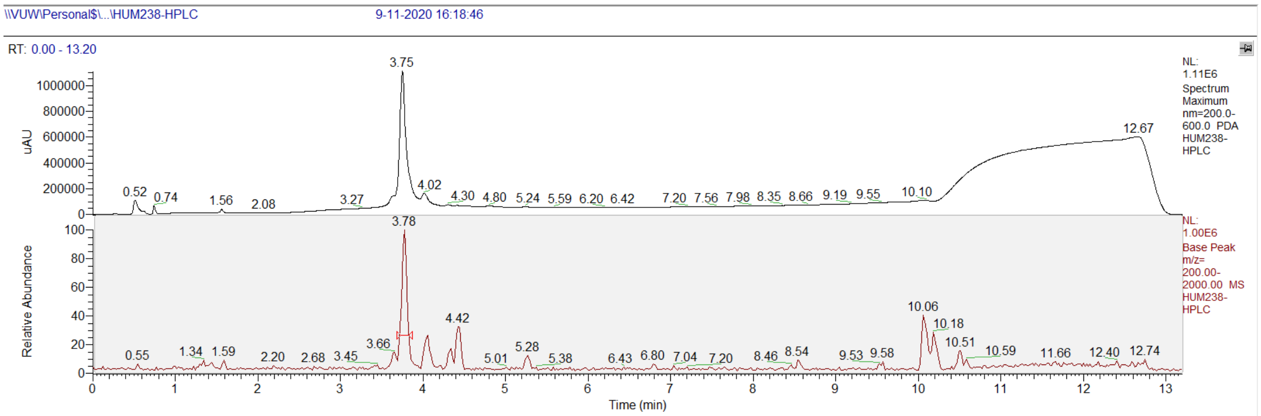
\\VUW\Personal$\...\HUM238-HPLC
9-11-2020 16:18:46
RT: 0.00 - 13.20
NL: 1.11E6 Spectrum Maximum nm=200.0-600.0 PDA HUM238-HPLC
NL: 1.00E6 Base Peak m/z= 200.00-2000.00 MS HUM238-HPLC
uAU
Relative Abundance
Time (min)

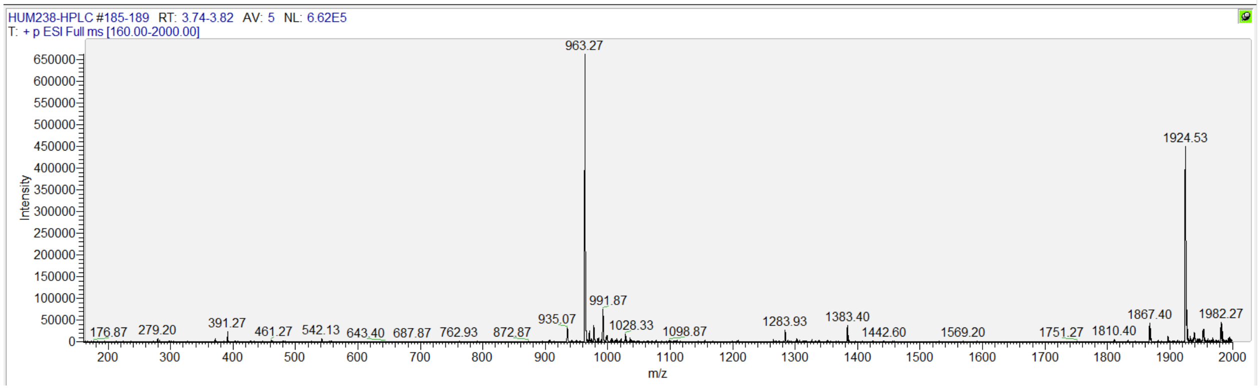
HUM238-HPLC #185-189 RT: 3.74-3.82 AV: 5 NL: 6.62E5
T: + p ESI Full ms [160.00-2000.00]
963.27
1924.53
Intensity
m/z

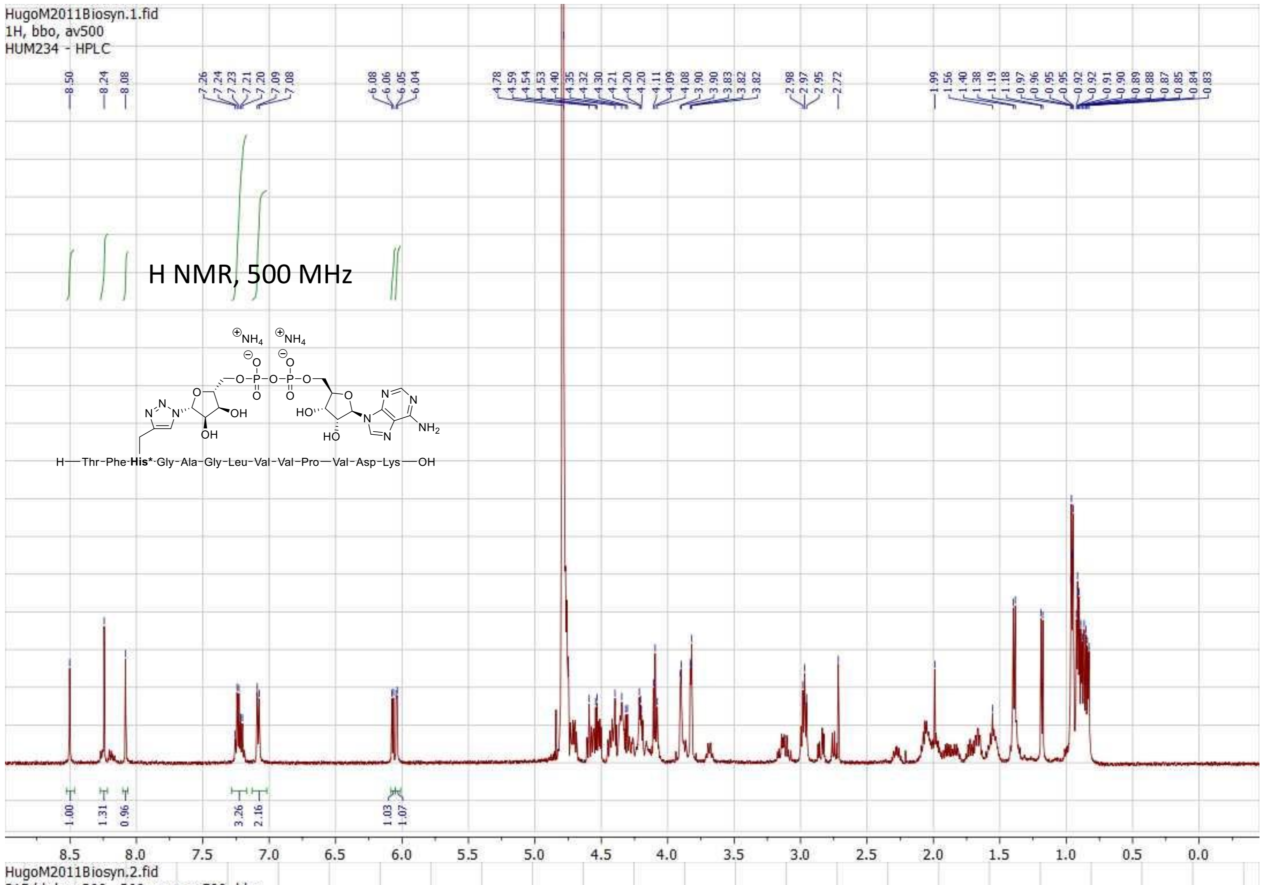

HugoM2011Biosyn.1.fid
1H, bbo, av500
HUM234 - HPLC
H NMR, 500 MHz
H—Thr-Phe-His*-Gly-Ala-Gly-Leu-Val-Val-Pro—Val-Asp-Lys—OH

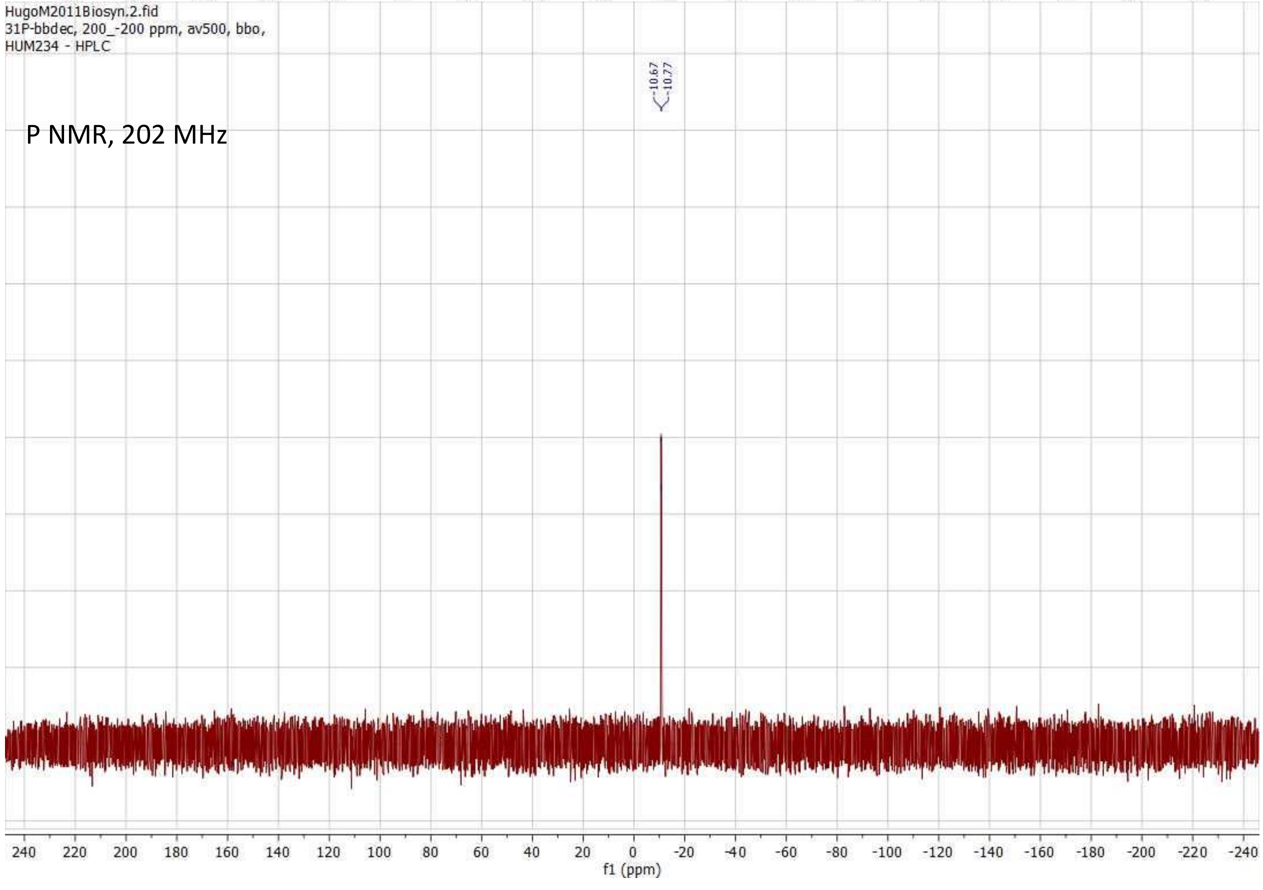

HugoM2011Biosyn.2.fid
31P-bbdec, 200_-200 ppm, av500, bbo,
HUM234 - HPLC
P NMR, 202 MHz
-10.67
-10.77
f1 (ppm)

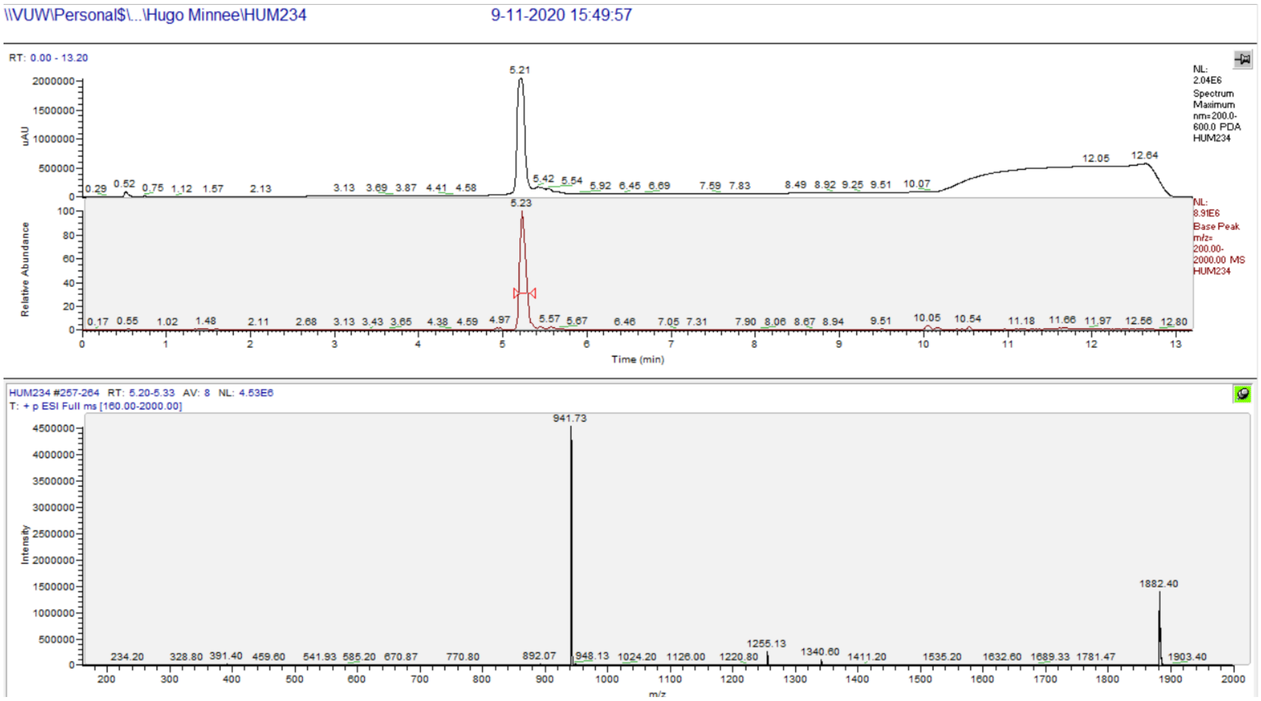
\\VUW\Personal$\...\Hugo Minnee\HUM234
9-11-2020 15:49:57
RT: 0.00 - 13.20
5.21
5.23
Time (min)
HUM234 #257-264 RT: 5.20-5.33 AV: 8 NL: 4.53E6
T: + p ESI Full ms [160.00-2000.00]
941.73
1882.40
1255.13
1340.60
Relative Abundance
Intensity
uAU
m/z

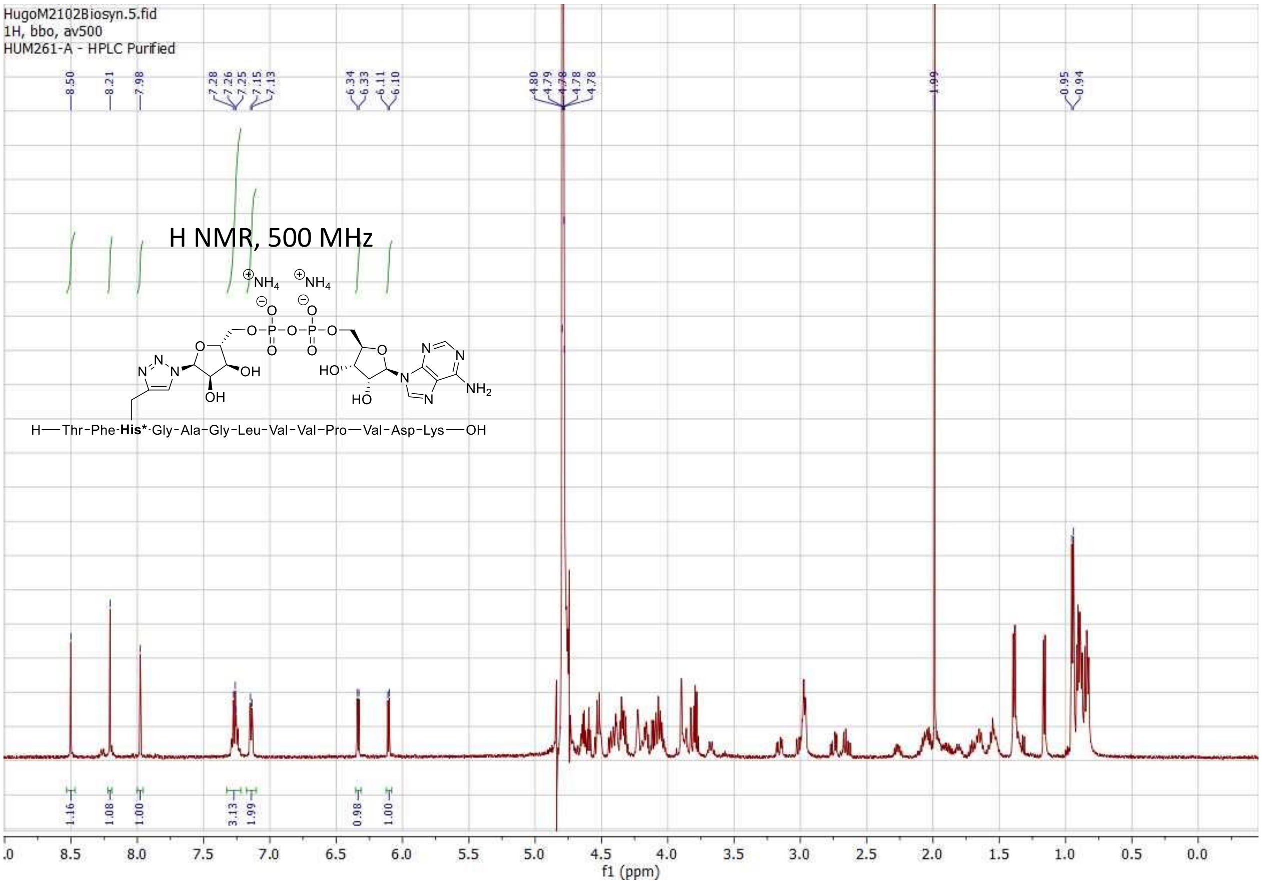
HugoM2102Biosyn.5.fid
1H, bbo, av500
HUM261-A - HPLC Purified
H NMR, 500 MHz
H—Thr-Phe-His*-Gly-Ala-Gly-Leu-Val-Val-Pro—Val-Asp-Lys—OH
f1 (ppm)

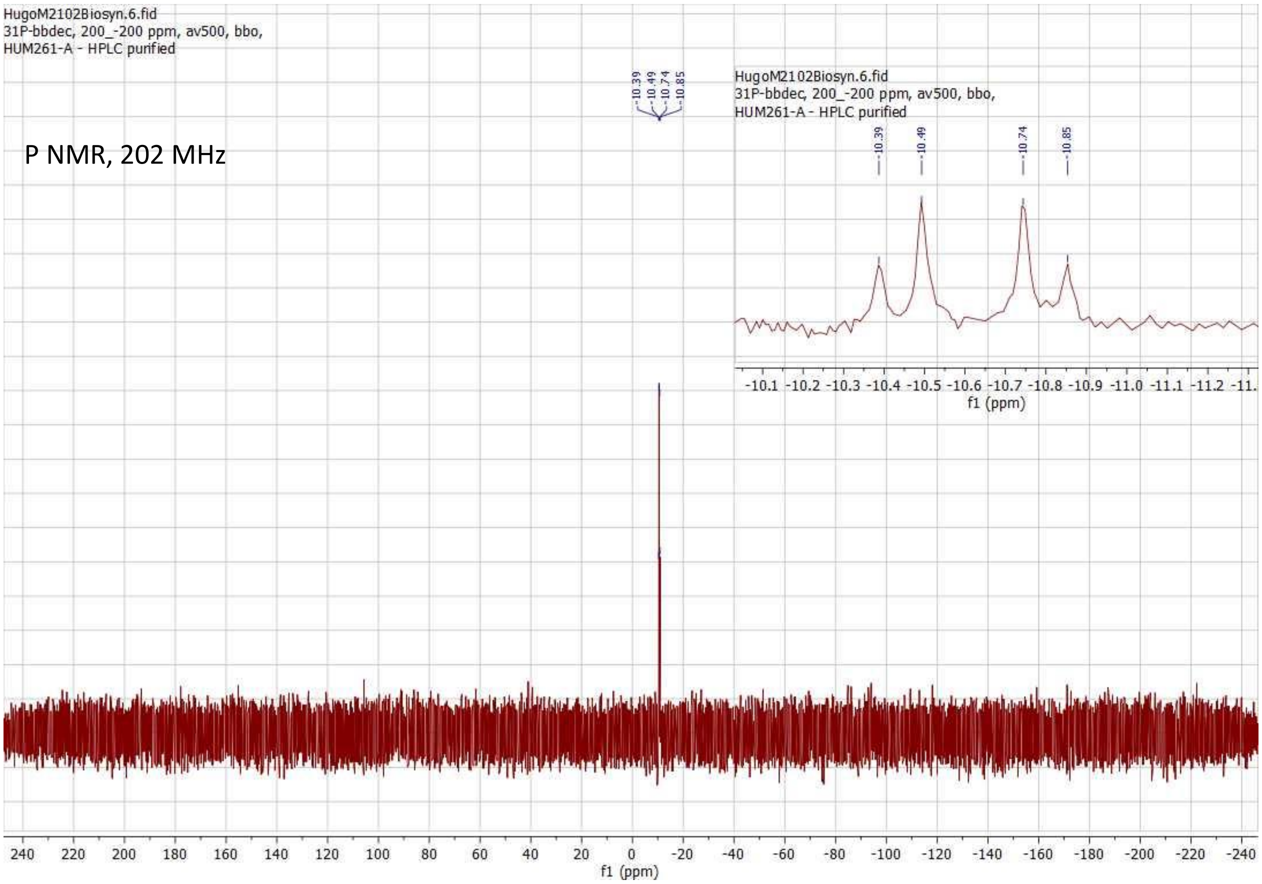
HugoM2102Biosyn.6.fid
31P-bbdec, 200_-200 ppm, av500, bbo,
HUM261-A - HPLC purified
P NMR, 202 MHz
-10.39
-10.49
-10.74
-10.85
f1 (ppm)

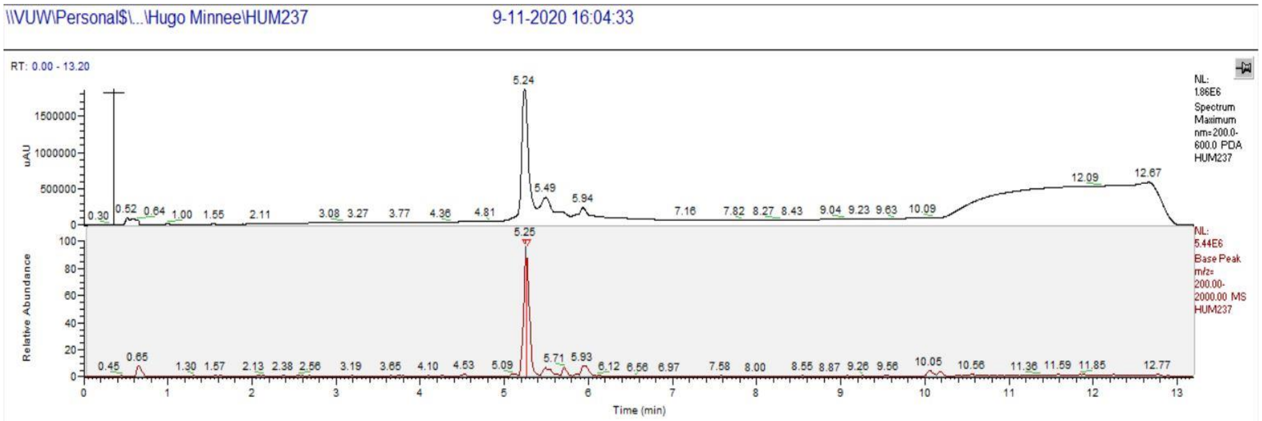
\\VUW\Personal$\...\Hugo Minnee\HUM237
9-11-2020 16:04:33
RT: 0.00 - 13.20
uAU
5.24
NL: 1.86E6 Spectrum Maximum nm=200.0-600.0 PDA HUM237
Relative Abundance
5.25
NL: 5.44E6 Base Peak m/z= 200.00-2000.00 MS HUM237
Time (min)

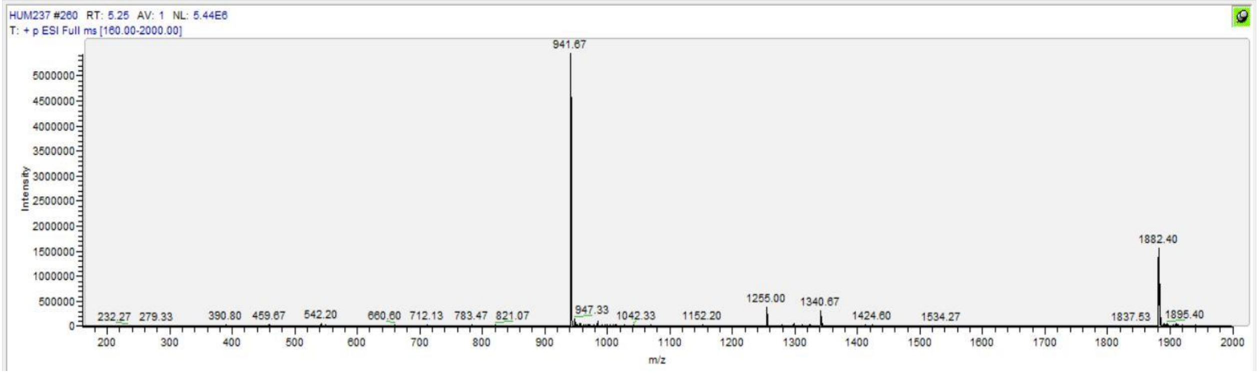
HUM237 #260 RT: 5.25 AV: 1 NL: 5.44E6
T: + p ESI Full ms [160.00-2000.00]
941.67
1882.40
1255.00
1340.67
Intensity
m/z

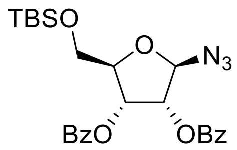

SHIMADZU

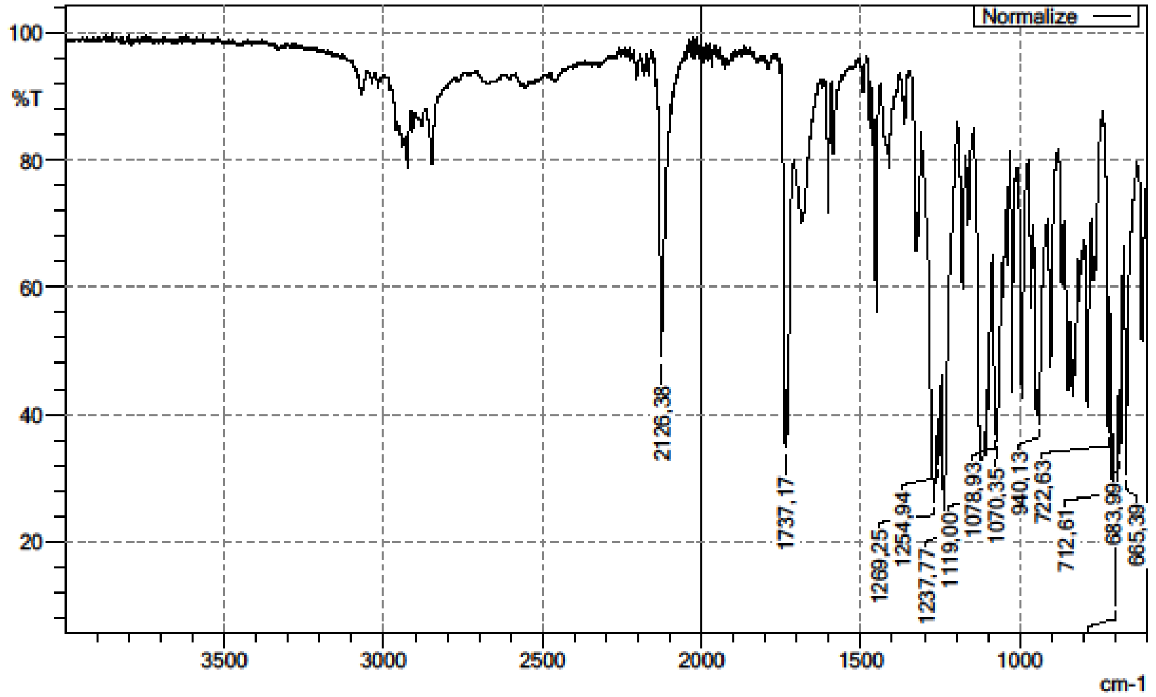

C:\LabSolutions\LabSolutionsIR\Data\HUM2021.ispd

	Item	Value
1	Comment	
2	Sample name	
5	Intensity Mode	%Transmittance
6	Apodization	None
7	Min	600
8	Max	4000
9	No. of Scans	15
10	Resolution	4 cm-1
11	FTIR Model	IRSpirit TOAPC0027956-Instrument1
13	Gain	1

	Peak	Intensity	Corr. Intensity	Base (H)	Base (L)	Area	Corr. Area	Comment
1	665,39	30,52	37,26	672,54	632,48	1494,672	373,997	
2	683,99	33,81	16,97	689,72	676,84	746,330	125,137	
3	699,73	10,00	28,14	706,89	689,72	1283,144	232,602	
4	712,61	29,79	14,65	716,90	709,75	422,003	40,056	
5	722,63	37,24	27,98	738,37	716,90	765,161	163,877	
6	940,13	38,59	8,03	944,42	914,37	1312,818	-1,817	
7	1070,35	35,95	10,89	1076,07	1054,61	1097,789	72,434	
8	1078,93	36,93	10,40	1086,09	1076,07	514,017	36,368	
9	1119,00	28,96	16,73	1147,62	1111,84	1645,756	216,574	
10	1237,77	23,29	28,48	1246,35	1194,84	2191,903	392,626	
11	1254,94	32,29	7,61	1257,80	1246,35	706,520	38,345	
12	1269,25	26,35	13,58	1309,31	1264,96	1914,326	105,372	
13	1737,17	34,72	16,44	1751,48	1734,30	609,914	65,283	
14	2126,38	49,03	39,59	2137,83	2097,76	1091,380	623,518	

8-1-2021 14:58:11

System Administrator

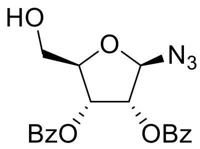

SHIMADZU

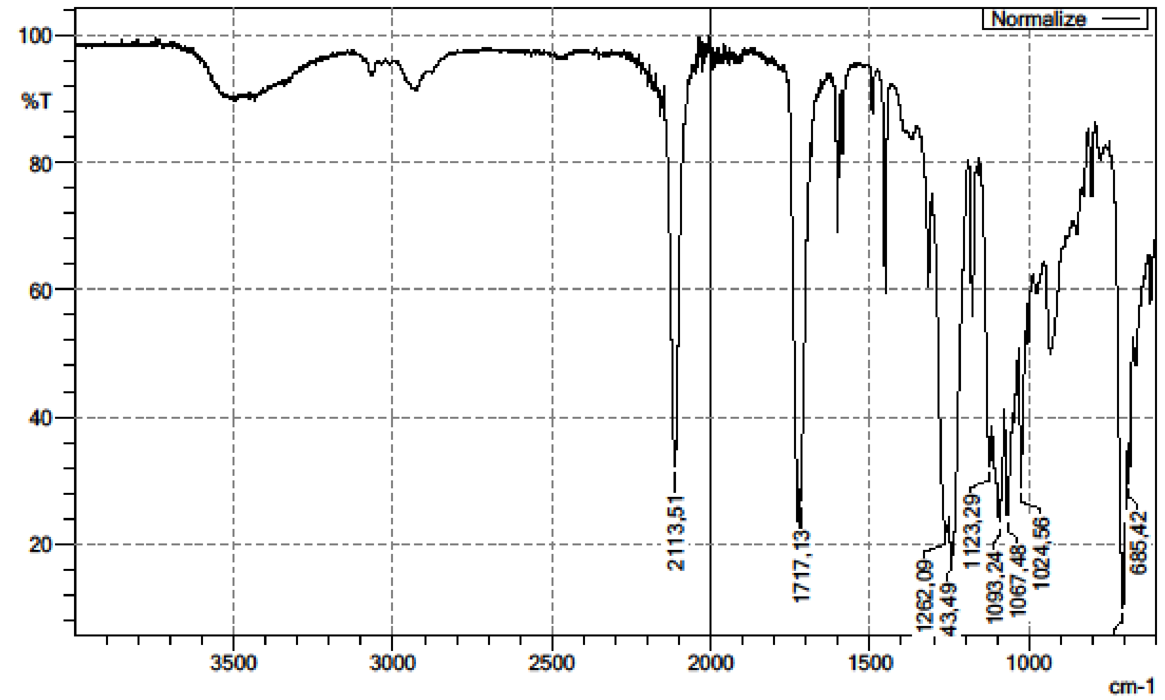

C:\LabSolutions\LabSolutionsIR\Data\HUM2023.ispd

	Item	Value
1	Comment	
2	Sample name	
5	Intensity Mode	%Transmittance
6	Apodization	None
7	Min	600
8	Max	4000
9	No. of Scans	15
10	Resolution	4 cm-1
11	FTIR Model	IRSpirit TOAPC0027956-Instrument1
13	Gain	1

	Peak	Intensity	Corr. Intensity	Base (H)	Base (L)	Area	Corr. Area	Comment
1	685,42	29,73	9,04	689,72	672,54	1037,340	57,007	
2	704,02	10,00	36,25	748,36	689,72	3019,925	583,108	
3	1024,56	28,88	22,89	1033,14	1004,52	1604,970	264,723	
4	1067,48	24,39	15,34	1076,07	1048,88	1828,517	186,404	
5	1093,24	23,50	10,70	1103,26	1076,07	1918,531	162,207	
6	1123,29	32,23	14,34	1154,77	1114,71	1801,648	143,856	
7	1243,49	18,31	0,77	1253,51	1242,06	909,523	2,751	
8	1262,09	22,13	1,88	1306,45	1260,66	2390,267	17,533	
9	1717,13	26,75	3,13	1718,56	1687,08	1389,861	-34,807	
10	2113,51	32,31	56,76	2147,85	2082,02	2346,662	1632,446	

8-1-2021 15:05:31

System Administrator

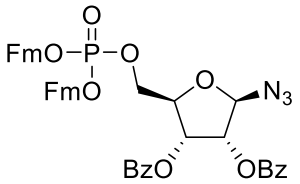

SHIMADZU

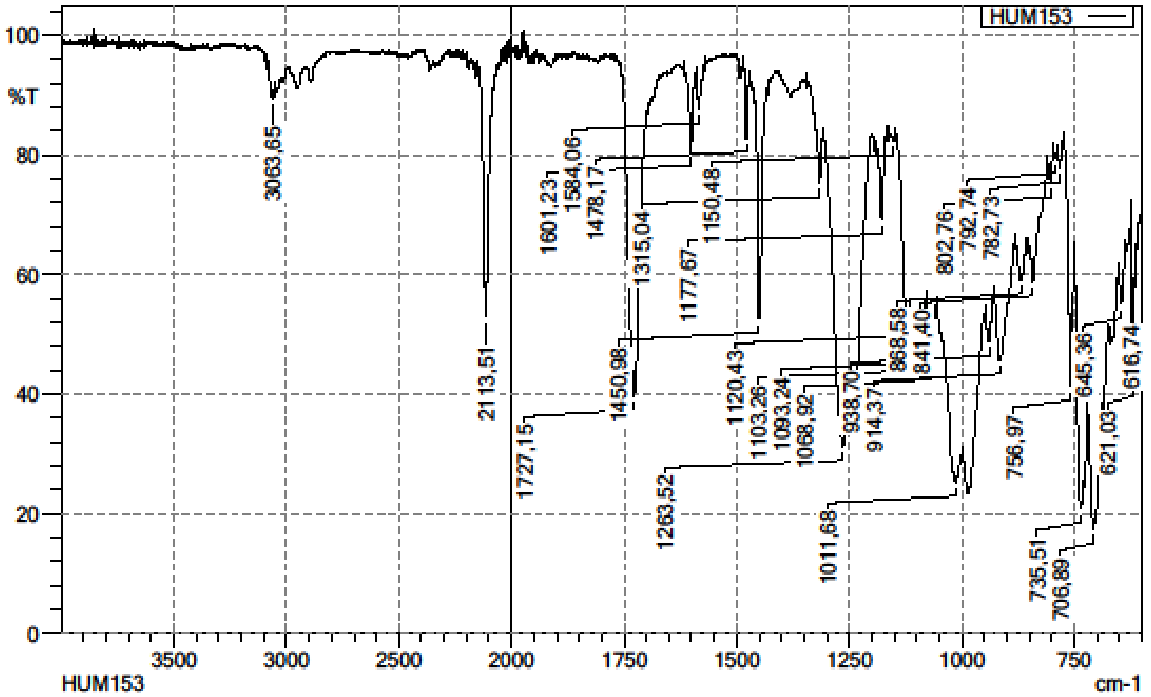

S:\Research data\IR\Hugo M\HUM153.ispd

	Item	Value
1	Comment	HUM153
2	Sample name	HUM153
5	Intensity Mode	%Transmittance
6	Apodization	None
7	Min	600
8	Max	4000
9	No. of Scans	15
10	Resolution	4 cm-1
11	FTIR Model	IRSpirit TOAPC0027956-Instrument1
13	Gain	1

	Peak	Intensity	Corr. Intensity	Base (H)	Base (L)	Area	Corr. Area	Comment
1	616,74	56,34	40,02 Div	618,17	613,88	174,925	159,301 Div	
2	621,03	41,98	54,38 Div	623,89	618,17	266,328	245,513 Div	
3	645,36	55,01	41,37 Div	651,08	635,34	643,956	586,922 Div	
4	706,89	17,40	79,01 Div	719,77	686,85	2413,443	2295,364 Div	
5	735,51	20,85	75,58 Div	749,81	728,35	1425,991	1349,438 Div	
6	756,97	41,34	55,11 Div	774,14	749,81	918,742	832,314 Div	
7	782,73	77,85	18,61 Div	787,02	774,14	258,127	212,513 Div	
8	792,74	79,37	17,10 Div	795,61	787,02	172,180	141,825 Div	
9	802,76	75,36	21,12 Div	808,48	795,61	285,676	240,226 Div	
10	841,40	58,83	37,67 Div	851,41	808,48	1374,260	1223,471 Div	
11	868,58	59,18	37,33 Div	881,46	855,70	976,071	886,189 Div	
12	914,37	45,79	50,75 Div	928,68	881,46	2176,488	2012,726 Div	
13	938,70	48,78	47,77 Div	948,72	928,68	963,864	894,789 Div	
14	1011,68	25,31	71,28 Div	1056,04	998,80	3569,387	3375,045 Div	
15	1068,92	44,81	51,82 Div	1078,93	1056,04	1124,039	1046,847 Div	
16	1093,24	47,75	48,90 Div	1097,53	1078,93	919,200	856,710 Div	
17	1103,26	46,32	50,33 Div	1114,71	1097,53	860,937	803,437 Div	

16-1-2020 15:47:36

System Administrator

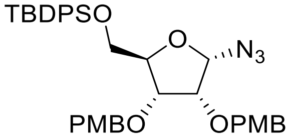

SHIMADZU

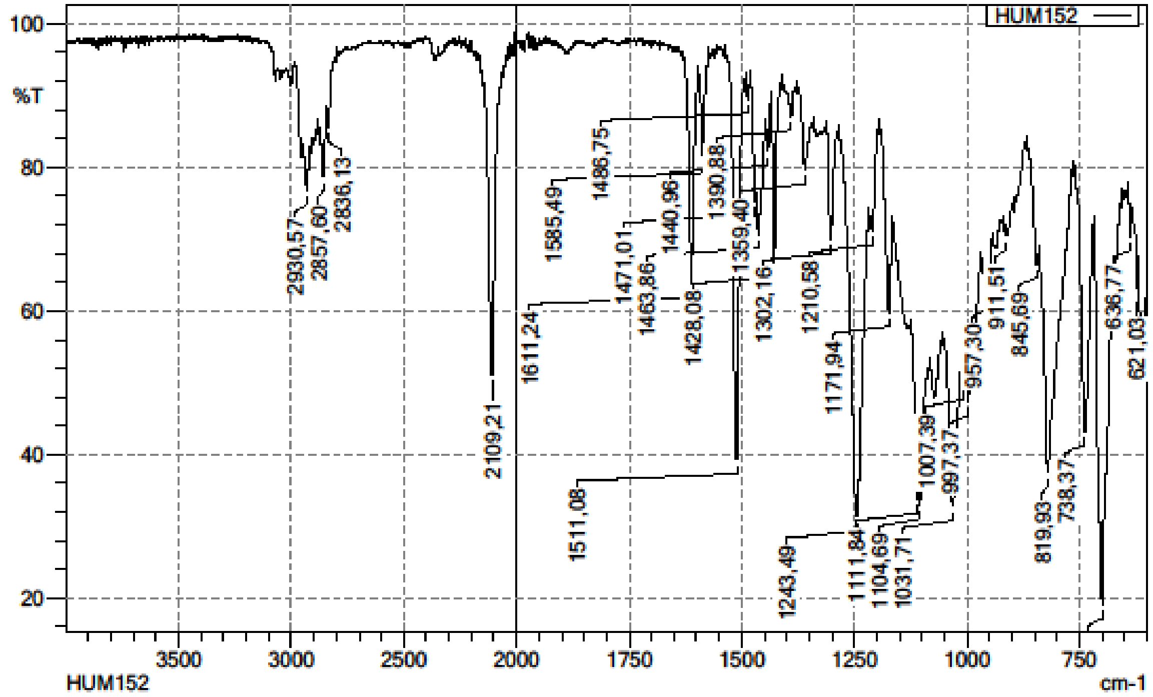

S:\Research data\IR\Hugo M\HUM152.ispd

	Item	Value
1	Comment	HUM152
2	Sample name	HUM152
5	Intensity Mode	%Transmittance
6	Apodization	None
7	Min	600
8	Max	4000
9	No. of Scans	15
10	Resolution	4 cm-1
11	FTIR Model	IRSpirit TOAPC0027956-Instrument1
13	Gain	1

	Peak	Intensity	Corr. Intensity	Base (H)	Base (L)	Area	Corr. Area	Comment
1	621,03	63,16	34,80 Div	632,48	619,60	396,083	369,688 Div	
2	636,77	70,36	27,59 Div	643,93	632,48	301,149	277,637 Div	
3	699,73	19,13	78,79 Div	718,33	669,68	2623,006	2522,123 Div	
4	738,37	43,23	54,68 Div	761,26	718,33	1644,919	1555,209 Div	
5	819,93	37,57	60,31 Div	839,96	765,56	3101,559	2944,405 Div	
6	845,69	66,54	31,34 Div	868,58	839,96	707,401	646,436 Div	
7	911,51	70,40	27,45 Div	917,24	897,20	554,025	510,974 Div	
8	957,30	62,11	35,72 Div	968,75	942,99	892,068	836,274 Div	
9	997,37	47,35	50,47 Div	1001,66	968,75	1455,797	1384,163 Div	
10	1007,39	49,62	48,20 Div	1013,11	1001,66	567,915	542,909 Div	
11	1031,71	32,75	65,06 Div	1053,18	1013,11	2223,311	2135,425 Div	
12	1104,69	32,98	64,80 Div	1107,55	1083,23	1352,136	1298,242 Div	
13	1111,84	33,76	64,02 Div	1126,15	1107,55	1001,498	960,144 Div	
14	1171,94	59,79	37,97 Div	1194,84	1164,79	811,881	744,409 Div	
15	1210,58	71,12	26,62 Div	1216,30	1194,84	483,531	435,141 Div	
16	1243,49	31,30	66,43 Div	1284,99	1216,30	2766,097	2610,154 Div	
17	1302,16	69,99	27,72 Div	1312,18	1284,99	553,609	491,421 Div	

16-1-2020 15:51:37

System Administrator

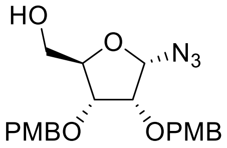

SHIMADZU

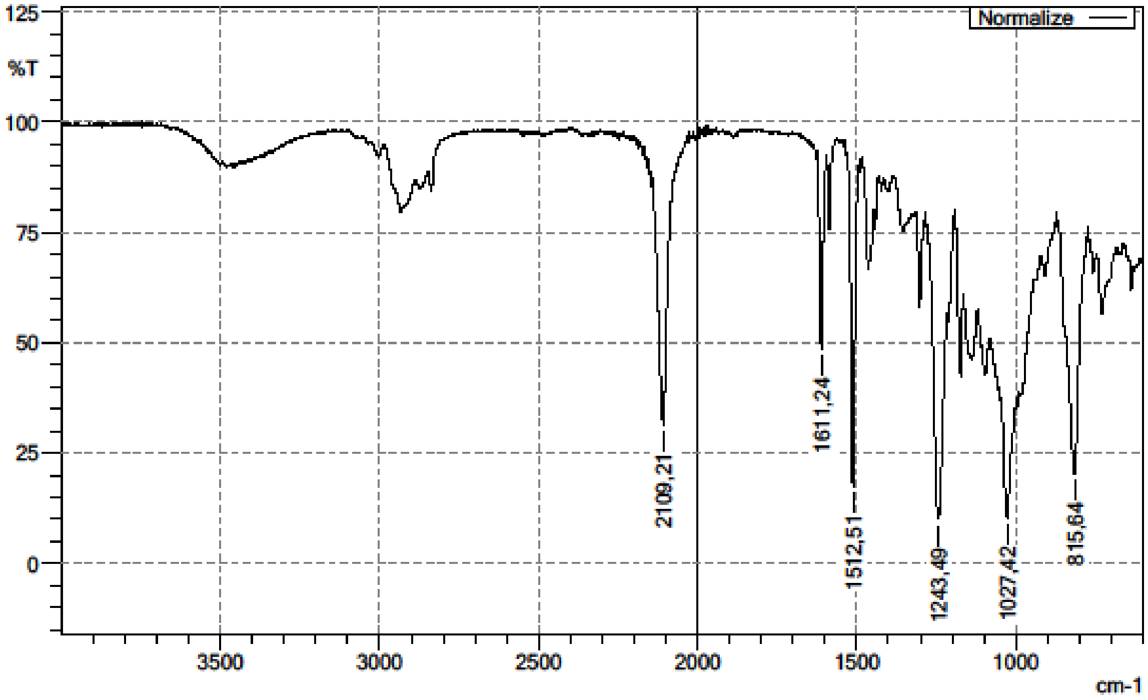

C:\LabSolutions\LabSolutionsIR\Data\HUM2025.ispd

	Item	Value
1	Comment	
2	Sample name	
5	Intensity Mode	%Transmittance
6	Apodization	None
7	Min	600
8	Max	4000
9	No. of Scans	15
10	Resolution	4 cm-1
11	FTIR Model	IRSpirit TOAPC0027956-Instrument1
13	Gain	1

	Peak	Intensity	Corr. Intensity	Base (H)	Base (L)	Area	Corr. Area	Comment
1	815,64	20,43	55,71	870,01	774,14	4600,709	2334,260	
2	1027,42	10,44	27,52	1048,68	994,51	4043,183	663,959	
3	1243,49	10,00	54,37	1284,99	1214,87	3947,963	1597,704	
4	1512,51	17,47	74,72	1533,97	1492,48	1384,524	1063,489	
5	1611,24	48,50	42,93	1625,55	1595,50	771,445	513,483	
6	2109,21	31,65	3,37	2143,56	2107,78	1431,371	-24,962	

8-1-2021 15:12:19

System Administrator

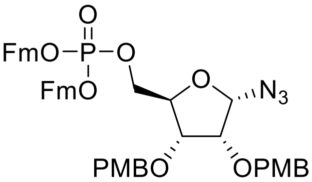

SHIMADZU

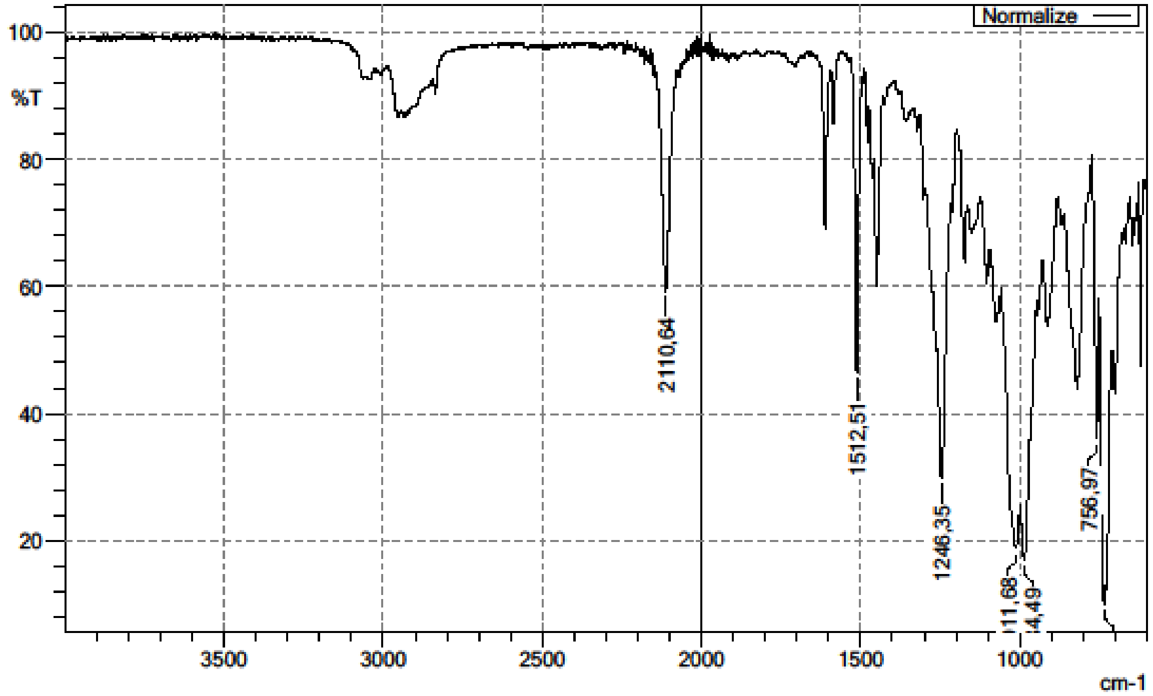

C:\LabSolutions\LabSolutionsIR\Data\HUM2024.ispd

	Item	Value
1	Comment	
2	Sample name	
5	Intensity Mode	%Transmittance
6	Apodization	None
7	Min	600
8	Max	4000
9	No. of Scans	15
10	Resolution	4 cm-1
11	FTIR Model	IRSpirit TOAPC0027956-Instrument1
13	Gain	1

	Peak	Intensity	Corr. Intensity	Base (H)	Base (L)	Area	Corr. Area	Comment
1	734,07	10,00	15,00	749,81	728,35	1649,070	258,441	
2	756,97	36,11	27,60	774,14	749,81	1047,953	277,924	
3	984,49	16,97	16,27	998,80	945,85	3540,041	395,182	
4	1011,68	18,82	12,87	1060,33	998,80	4104,525	510,936	
5	1246,35	30,00	28,07	1262,09	1214,87	2391,884	577,781	
6	1512,51	46,47	47,74	1529,68	1492,48	805,104	587,399	
7	2110,64	59,76	1,93	2112,07	2079,16	689,718	-82,312	

8-1-2021 15:08:57

System Administrator
